# Psilocybin elicits a conserved glucocorticoid-responsive gene signature across five 5-HT2A receptor-rich brain regions in rat

**DOI:** 10.1017/neu.2026.10075

**Published:** 2026-04-10

**Authors:** Ashkan Veysi, Daniela Atanasovski, Maryam Ardalan, Nasrin Motamed, Elias Eriksson

**Affiliations:** 1 Department of Cell and Molecular Biology, University of Tehran, Iran; 2 Department of Pharmacology, Institute of Neuroscience and Physiology, University of Gothenburg, Sweden; 3 Department of Physiology, Institute of Neuroscience and Physiology, University of Gothenburg, Sweden

**Keywords:** hallucinogens, psychedelics, psilocybin, gene expression, 5-HT2A receptors

## Abstract

**Objective:**

Psychedelics such as psilocybin are known for their hallucinogenic properties and have also been reported to produce long-lasting therapeutic effects in depression and possibly also other psychiatric disorders. Several lines of evidence suggest that psilocybin exerts its effects through activation of 5-HT2A receptors located postsynaptically to serotonergic neurons, for example, in the frontal cortex, parts of the limbic system, including the amygdala and hippocampus, and striatum. The present study was conducted to shed further light on psilocybin-induced changes in gene expression.

**Method:**

Samples from the medial prefrontal cortex, cingulate cortex, hippocampus, amygdala, and striatum were collected from 24 male Wistar rats 90 min after they had been injected with either saline or psilocybin (2 mg/kg) and subjected to multi-region transcriptional profiling using 3prime-RNASeq technology.

**Results:**

*Nfkbia* and *Sgk1* were upregulated in all the studied regions, *Ddit4* was upregulated in four regions, and *Gpd1*, *Apold1*, *Sox9*, *Tsc22d3*, and *Slc2a1* were differentially expressed in two regions. Other cases of differentially expressed genes were region-specific.

**Conclusion:**

Whereas psilocybin was not found to alter the expression of genes encoding enzymes, transporters, or receptors implicated in the serotonergic signalling, or those specifically involved in the regulation of the synaptic activity of other neurotransmitters, a common denominator for many of the genes impacted by psilocybin is that they have previously been found to be activated by glucocorticoids.

## Graphical abstract

**Figure:**
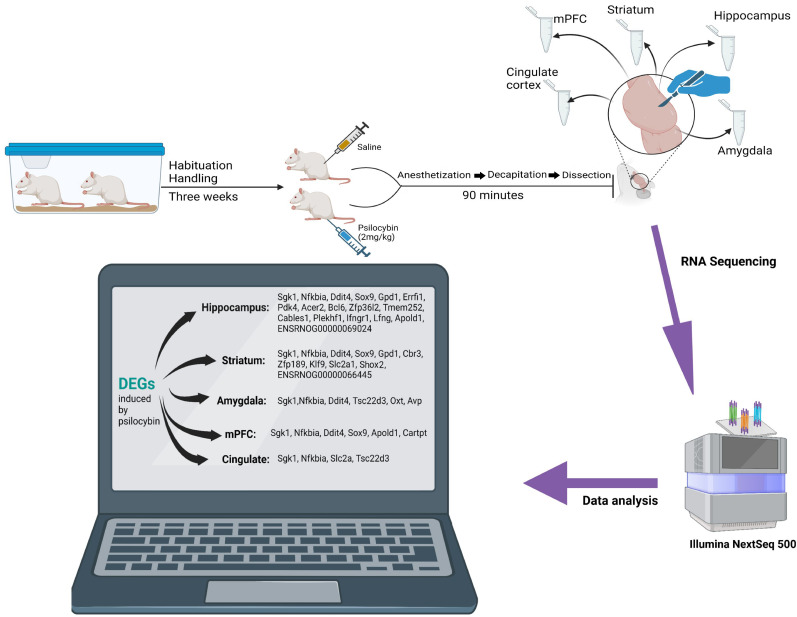

Significant outcomes

- A single dose of psilocybin impacts gene expression similarly in several different 5-HT2A-rich brain regions.
- Several of the genes influenced by psilocybin in more than one region are reportedly involved in nerve cell plasticity and/or regulated by glucocorticoids.
- Except for *Cartpt*, which was upregulated in the median prefrontal cortex, psilocybin did not alter the expression of genes specifically involved in the synaptic machinery of monoaminergic neurotransmittors, glutamate, or GABA, respectively.

Limitations

- Changes in gene expression do not necessarily translate into significant changes in the level of the corresponding protein.
- To what extent the observed changes are sufficiently long-lasting to contribute to the possible long-lasting clinical effects of psilocybin is unclear.
- Only male animals were studied.

## Introduction

Psychedelics are a class of powerful psychoactive substances which substantially alter sensory and cognitive processes, mood, and sense of self (Nichols, 2016). In recent years, psychedelics have gained renewed interest as a possible treatment for a wide range of mental disorders. A single dose of one of these compounds, some of which are naturally occurring, thus has been claimed to cause an immediate improvement in depressed patients which may persist for several months and they have also been suggested useful for the treatment of anxiety, addiction, obsessive compulsive disorder, and cluster headaches (Carhart-Harris *et al*., 2016; Carhart-Harris *et al.*, 2021; Davis *et al.*, 2021; Goodwin *et al.*, 2022; von Rotz *et al*., 2022). Psilocybin, the major psychoactive ingredient of hallucinogenic mushrooms, has been one of the most commonly used psychedelic compounds in human studies owing to its relative physical safety, good absorption following oral administration, and moderate duration of acute action (Hasler *et al.*, 2004; Johnson *et al.*, 2008). A substantial body of evidence from preclinical experiments, brain imaging, and clinical studies using serotonin receptor antagonists supports the notion that the impact of psilocybin is due to signalling cascades downstream of its targeted serotonergic receptors, predominantly 5-HT2A receptors, which are activated by an active metabolite of psilocybin – psilocin (Quednow *et al.*, 1974; González-Maeso *et al.*, 2007; Kometer *et al.*, 2012; Vollenweider *et al.*, 1998; Kwan *et al.*, 2022). Also for other psychedelics, such as lysergic acid (LSD), 5-HT2A receptor activation appears to be the primary mechanism of action (Preller *et al.*, 2017; Holze *et al.*, 2021).

It is noteworthy that the alleged antidepressant impact of psilocybin, unlike that of conventional antidepressants (Duman and Aghajanian, 2012), is manifested within minutes after administration and persisting for months. To explain these persisting effects, various theories have been proposed (Preller & Volenweider, 2018) including a suggested impact of psilocybin on gene expression (Alberini, 2009). One group of genes of particular interest in this context could be those related to the synaptic regulation of neurotransmitters such as glutamate, GABA, dopamine, and serotonin; another group might be those impacting neuronal plasticity. Also suggested as a possible target of psilocybin is the hypopthalamus-adrenal-axis. Whereas glucocorticoids bind to receptors in the brain and regulate the transcription of numerous genes involved in neuronal survival, metabolism, synaptic function, and plasticity (Juszczak & Stankiewicz, 2018), previous studies have thus shown that administration of psilocybin and other psychedelics acutely activates the hypothalamic-pituitary-adrenal axis, leading to an increase in glucocorticoid levels in both humans (Hasler *et al.*, 2004) and rodents (Jones *et al.*, 2023). This raises the possibility that the transcriptional effects of psilocybin may be mediated, at least in part, through engagement of glucocorticoid signalling pathways alongside other potential mechanisms.

While previous studies regarding the impact of psilocybin on gene expression have focused on specific regions, or examined only a restricted set of candidate genes (Martin & Nichols, 2018; Jefsen *et al.*, 2021; Wulff *et al.*, 2023; Sumner & Lukasiewicz, 2023; Davouidian *et al.*, 2023; Rijsketic *et al.*, 2023), the present study used 3prime-RNA sequencing technology in order to obtain a multi-regional transcriptional profile following a single dose of psilocybin in five 5-HT2A receptor-rich areas of the adult male rat brain – hippocampus, amygdala, striatum, medial prefrontal cortex, and cingulate cortex (Pazos *et al.*, 1987; Sadzot *et al.*, 1995; Smith *et al.*, 1998; Hensler, 2012).

## Material and methods

### Animals

A total of 24 male Wistar rats obtained from Envigo, Netherlands, were group-housed (2 per cage) and kept under standard laboratory conditions (22°C ± 1°C, 60% relative humidity, 12–12 h light–dark cycle with light on at 07:00 A.M., food and water ad libitum). One week after arrival, animals were left undisturbed for the sake of habituation to the facility and to allow daily assessments of their welfare by animal caretakers. In the subsequent week, the rats were handled for 15 min every other day, with daily handling during the week leading up to their treatment. The animals were 10–12 weeks upon arrival and weighed 450 ± 50 g on the day of the experiment. All experiments were conducted in accordance with the legislative directive 2010/63/EU of the European Union on the care and use of laboratory animals for research purpose and approved under permit number 3703/21 by the regional animal ethics committee at University of Gothenburg under the guidelines of the Swedish Board of Agriculture.

### Treatment, sample size, and collection

After the final handling, animals were injected subcutaneously with either saline or psilocybin (2 mg/kg) (LAB Sweden, Stockholm, Sweden) dissolved in 0.9% saline – a dose selected based on previous studies in rat (Davis and Walters, 1977; Jefsen *et al.*, 2021; Shahar *et al.*, 2022). No formal blinding was used. Investigators were aware of the treatment group allocation during all experimental procedures. Treatment assignment was arbitrary, with one animal in each cage receiving psilocybin and the other receiving saline.

The sample size (*n* = 12 per group) was determined to be sufficient using an RNA-seq power calculation tool (https://cqs-vumc.shinyapps.io/rnaseqsamplesizeweb/) (Zhao *et al.*, 2018). The calculation was set to control the false discovery rate (FDR) at 0.05 with a target power of 80%. Input parameters, informed by similar rodent brain transcriptomic studies, were defined as follows: a total of 15 000 tested genes (m), among which 150 were expected to be prognostic (m1), with a minimum fold change (rho) of 2. The average read count (lambda0) for these genes was set to 10 and the dispersion (phi0) to 0.07. The ratio of normalisation factors (w) was set to 1. Based on these parameters, the estimated sample size required was 12 subjects per group.

Ninety minutes after administration of psilocybin or saline, animals were administered a lethal dose of sodium pentobarbital (150 mg/kg) (Kronans Apotek, Gothenburg, Sweden) for tissue collection, as outlined in the experimental design (Supplement I, Figure S1). Cages, each housing one saline- and one psilocybin-treated rat, were processed sequentially. For the first cage, the hemisphere to be sampled (left or right) was selected arbitrarily. For each subsequent cage, the sampled hemisphere was alternated from the previous one. This alternating protocol ensured a balanced distribution, yielding, for each brain region, 6 samples per treatment from the left hemisphere and 6 from the right hemisphere. Consequently, the total number of samples for each treatment group was 60 (5 regions × 12 rats). To evaluate whether lateralisation influenced the transcriptional response to psilocybin, we performed differential expression analyses both with pooled hemispheres and separately for left and right hemispheres, respectively (Supplement I, Table S1).

All samples from the five regions of interest (hippocampus, amygdala, striatum, medial prefrontal cortex, and cingulate cortex) were collected from each animal during a single session on the same day. All samples were promptly stored at −80°C under identical conditions to ensure consistent biological integrity across regions.

### Sample processing and batch effect analysis

Next, samples were processed for RNA extraction, library preparation, and RNA-sequencing. All laboratory work was conducted at TATAA Biocenter (Gothenburg, Sweden). For logistical reasons, the processing of the hippocampal samples was performed in a separate batch one year after the other four regions (amygdala, striatum, medial prefrontal cortex, and cingulate cortex). This resulted in minor differences in the specific kits and reagents used for the hippocampus from the RNA extraction step onwards; these differences were purely logistical and did not reflect any difference in experimental treatment, sampling, or storage conditions, which were identical for all regions.

To objectively assess whether this technical variation introduced a significant batch effect that could confound the biological interpretation of our data, we performed principal component analysis (PCA) on the variance-stabilised transformed count data of the entire dataset during quality control. The resulting PCA plot is presented in Supplement I (Figure S2).

Due to the separate processing batches, the methodological details for the hippocampus are described separately from those for the other four regions in the sections below.

### RNA extraction

#### Striatum, amygdala, cingulate cortex, and medial prefrontal cortex

Ninety-five samples were extracted with the QiaSymphony RNA kit (Cat# 931636; Qiagen, Hilden, Germany) using the extraction robot QiaSymphony (Qiagen). The samples were randomly divided into four batches for the extraction using one extraction no template control (NTC) in each run. Due to a technical issue, one sample was not eluted by the extraction robot. The 94 samples successfully extracted were quantified with spectrophotometer.

#### Hippocampus

RNA extraction was carried out using the Fatty Tissue RNA Purification Kit (Cat# 36200; Norgen Biotek, Ontario, Canada) according to the manufacturer’s instructions manual (Fatty Tissue RNA Purification Kit, Doc. PI36200-8; Norgen Biotek). The tissues were disrupted using Bead Tubes (Cat# 26533; Norgen Biotek) in a TissueLyser II (Qiagen) for 2 × 5 min (25 Hz). One extraction NTC was included in the extraction. Each sample was eluted in a total volume of 50 μl. Two samples were lost during the extraction process.

### RNA quality control, quantification, and normalisation

#### Striatum, amygdala, cingulate cortex, and medial prefrontal cortex

For all RNA samples, the RNA integrity was assessed using capillary gel electrophoresis DNF-471-Standard Sensitivity RNA 15 nt kit (Fragment Analyzer, Agilent, Santa Clara, California, United States) and their concentrations determined by spectrophotometry (Lunatic, Unchained Labs, Pleasanton, California, United States). RNA concentrations of the samples ranged between 3.6 and 142 ng/μl. The capillary gel electrophoresis measurements resulted in RNA integrity number (RIN) values above 8 for all the samples. Negative controls showed no contamination. Based on the measured concentrations, all samples were normalised to a final concentration of 5 ng/μl. A summary of the results of RNA quality control measurements of all samples is provided in Supplement IV. Chromatograms of all samples are also available in Supplement II. Fragment analysis data are available upon request from the authors.

#### Hippocampus

The concentration and purity of eluted RNA were measured with a spectrophotometer (Lunatic, Unchained Labs) and RNA integrity was determined by capillary gel electrophoresis (Fragment Analyzer, Agilent) using the RNA Standard Sensitivity Kit (Cat# DNF-471; Agilent Technologies Inc., Santa Clara, California, United States). RNA concentrations varied between 51 and 429 ng/μl and RQNs were between 7.9 and 10.0. Negative controls showed no contamination. All samples passed TATAA’s internal quality criteria for RNA quality (Supplement IV, Extraction QC sheet). The majority of samples (77.1%) passed TATAA’s internal quality criteria for RNA purity (Supplement IV, Extraction QC sheet). Based on the measured concentrations, all samples were normalised to a final concentration of 50 ng/μl. Samples with values below the target normalisation concentration were not diluted prior to library preparation.

### Library preparation

#### Striatum, amygdala, cingulate cortex, and medial prefrontal cortex

The 94 normalised RNA samples were transferred into a 96-well PCR plate with one library NTC included. A Universal Human Reference (UHR) sample was included in the preparation as a positive control. The sample input volume was 4.5 μl sample combined with 0.5 μl of the External RNA Controls Consortium spike control (1:10 000 dilution according to the kit recommendation; Cat# 445670; Thermo Fisher Scientific, Waltham, Massachusetts, United States). Libraries were generated and purified using the QuantSeq 3’ mRNA-Seq Library Prep kit FWD with Unique Dual Indices (Cat# 191.96; Lexogen, Wien, Austria), including unique molecular identifiers (UMI) using Second Strand Synthesis Module for QuantSeq FWD (Cat# 081.96; Lexogen), and Lexogen UDI 12 nt Unique Dual Indexing Add-on Kits: A2 (UDI 12A 0097-0192, Cat# 119.384; Lexogen). Of the 94 samples taken through library preparation, 42 yielded libraries that were qualified to proceed to sequencing. For five samples, no viable libraries could be observed in the Fragment Analyzer electropherograms (<1.5 nM). Thirty-two under-cycled libraries were successfully reamplified so that they reached a yield sufficient for pooling and sequencing (>1.5 nM). Library preparation starting from the extracted total RNA was performed in 14 samples of which one still did not pass the yield sufficient for pooling (>1.5 nM). Library reamplification and reruns thus resulted in 51 libraries gaining acceptable quality and quantity so that in total ninety-three samples could be included in subsequent pooling and NovaSeq sequencing runs.

#### Hippocampus

Sequencing libraries from the isolated total RNA were generated using the QuantSeq 3′ mRNA-Seq Library Prep Kit (Cat# 015.24; Lexogen) in combination with unique dual indexes (UDI) (Cat# 198.94; Lexogen) and UMI (Cat# 081.96; Lexogen) according to the manufacturer’s protocol. A UHR (Cat# QS0639; Thermo Fisher Scientific) was included in the preparation as a positive control and a negative NTC consisting of RNase-free water was included on each plate.

### Library quality control

#### Striatum, amygdala, cingulate cortex, and medial prefrontal cortex

A PCR Add-on Kit (Cat# 020.96; Lexogen) together with SYBR Green I (Cat# S7563; Thermo Fisher Scientific) was used to establish a qPCR assay with the purpose of determining the optimal number of cycles for the endpoint PCR of the libraries and revealed this to be 18 (CFX96, Bio-Rad, Hercules, California, United States). The quality of all amplified libraries was controlled by capillary gel electrophoresis (Fragment Analyzer, Agilent) using High Sensitivity NGS Fragment Analysis Kit (Cat# DNF-474; Fragment Analyzer, Agilent). The library quantity was determined with qPCR on the QuantStudio 12K Flex platform (Thermo Fisher Scientific) using TATAA’s NGS Library Quantification Kit (Cat# TA20-NGSQ; TATAA Biocenter, Gothenburg, Sweden). The libraries were diluted 10 000X prior to quantification and all samples were run in triplicate reactions in the qPCR analysis. A standard curve consisting of 6 points run in triplicate and NTCs consisting of RNase-free water were included in all qPCR runs. However, the data from the qPCR did not correlate with the fragment analysis data, suggesting a possible problem of inhibition; therefore, only the fragment analysis data were used for the following steps. The qPCR cycling programme is outlined in Supplement I (Table S2). A Reamplification Add-on Kit (Cat# 080.96; Lexogen) was used to further amplify libraries with insufficient yields for pooling and sequencing (<1.9 nM). 49 libraries were under-cycled and subjected to 3 or 5 cycles of reamplification PCR. The quality and quantity of the reamplified libraries were checked with capillary gel electrophoresis (Fragment Analyzer, Agilent). For 14 samples where no viable libraries were produced due to either low yield, low quality, or both, samples were once again taken through library preparation, starting from the total RNA; for 10 of these, this resulted in improved library quality that passed the internal quality threshold (>1.5 nM). All samples were included in the sequencing.

#### Hippocampus

The optimal number of cycles for amplification, that is 14, was set by qPCR I (CFX Opus 96, Bio-Rad, Hercules, California, United States) using PCR Add-on Kit (Cat# 020.24; Lexogen) and SYBR Green I (Cat# S7563; Thermo Fisher Scientific). The qPCR cycling programme is outlined in Supplement V (PCR sheet). Generated libraries were quality-controlled using capillary gel electrophoresis with the High Sensitivity NGS Fragment Analysis Kit (Cat# KitDNF-474; Fragment Analyzer, Agilent). Library concentrations were also determined by qPCR II (QuantStudio 7, Thermo Fisher Scientific) using the TATAA NGS Library Quantification Kit (Cat# TA20-NGSQ; TATAA Biocenter) and TATAA SYBR GrandMaster Mix with low Rox (Cat# MixTA01-625; TATAA Biocenter). Libraries were diluted 10 000X prior to quantification and run in triplicate reactions. A qPCR NTC consisting of RNase-free water was included in triplicate reactions. The library quantification qPCR master mix protocol and cycling programme are detailed in Supplement V (PCR sheet). Library quality control is detailed in Supplement V (Library QC sheet). Electropherograms are presented in Supplement II. The concentration of the libraries was calculated from the qPCR according to the formula: c(pM) = c’(pM) × 415 / (avg. fragment length) (bp) × 10000, where c’(pM) is the uncompensated concentration detected by the qPCR analysis and standard curve-based unit conversion. Generated libraries were normalised to 2 nM and pooled together for loading on the sequencer.

### Pooling and sequencing

#### Striatum, amygdala, cingulate cortex, and medial prefrontal cortex

The sequence-ready libraries were normalised to a concentration of 1.5 nM and pooled. For loading uniformity, the concentration of the library pools was measured using the Quant-it PicoGreen dsDNA Assay Kit (Cat# P7589; Thermo Fisher Scientific) on a fluorescence spectrophotometer (NanoDrop ND3300; Thermo Fisher Scientific). The quantified pools were diluted and denatured to a loading concentration of 300 pM with a 20% PhiX spike-in control (Cat# 15017666; Illumina, San Diego, California, United States) according to the NovaSeq 6000 Denature and Dilute Libraries Guide (Document# 1000000106351 v03; Illumina,). Each pool was single-end sequenced (100 cycles) in one lane on the S1 flow cell using S1 Reagent Kit v1.5 cycles, 100 cycles (Cat# 20028312; Illumina) according to the NovaSeq XP workflow in the NovaSeq 6000 Sequencing System Guide (Document# 1000000019358 v14; Illumina).

#### Hippocampus

For loading uniformity, the concentration of the final library pool was measured using the Quant-it PicoGreen dsDNA Assay Kit (Cat# P7589; Thermo Fisher Scientific) on a fluorescence spectrophotometer (NanoDrop ND3300, Thermo Fisher Scientific). The quantified pool was then diluted to a loading concentration of 1.8 pM, denatured, and spiked-in with PhiX (Cat# FC-110-3001; Illumina) in accordance with the NextSeq System Denature and Dilute Libraries Guide (Document# 15048776 v18; Illumina). The pool was sequenced on the NextSeq500 sequencing platform (Illumina) using NextSeq 500/550 Mid Output Kit v2.5 (75 Cycles) (Cat# 20024904; Illumina) and a read mode setting of 1 × 75 bp. Reads quality score yielded a ≥Q30 score of 89.7% and an average of 3.1 *M* single-end reads per sample (Supplement V, Sequencing QC sheet). A summary of sequencing quality control is presented in Supplement I (Table S3).

### Quality control and pre-processing of sequencing data

#### Striatum, amygdala, cingulate cortex, and medial prefrontal cortex

Raw data fastq files were created using bcl2fastq v2.20.0.422 and then further processed following the recommended workflow on the Lexogen BlueBee QuantSeq platform. UMIs were identified using UMI-tools v1.1.2, and sequencing adapters and low-quality bases were trimmed with BBMap/BBDuk v38.96. Quality parameters of raw sequencing data were then visually investigated from the results of FastQC v0.11.9 and summarised in a series of html reports from MultiQC v1.12 (Supplements VI-VIII). The NovaSeq run fulfilled Illumina’s specification with respect to data output and quality. The cluster count (passing filter) on the flowcell lane was 0.920 million and the percentage of bases with a quality score ≥Q30 was 89.71%. The data yield (total number of reads) and quality per sample are outlined in Supplement IV.

#### Hippocampus

Fastq files were created using bcl2fastq. Read quality parameters of raw sequencing data were then visually investigated from the results of FastQC and summarised in a series of html reports from MultiQC (Supplements IX-XII).

### Alignment and gene counts

#### Striatum, amygdala, cingulate cortex, and medial prefrontal cortex

Trimmed reads were aligned to the rat reference genome (mRatBN7.2) with Star v2.7.10. To remove true PCR duplicates, the UMIs were used to deduplicate aligned reads with UMI-tools v1.1.2, and the remaining reads per gene were counted by means of HTSeq v2.0.1. The following quality parameters were calculated in QoRTs v1.3.6 and visualised in MultiQC reports (Supplements VI–VIII) or figures (Supplement II, QC1 section): (i) percent genes with counts, (ii) cumulative gene assignment, (iii) percent reads in genes coding region (CDS) and untranslated region (UTR), (iv) gene body coverage, and (v) strandedness.

#### Hippocampus

Fastq files were further processed following the recommended workflow by Lexogen – QuantSeq data analysis guide. UMIs were identified using UMI-tools and sequencing adapters and low-quality bases were trimmed using BBMap/BBDuk. Quality parameters of trimmed sequencing data were then again visually assessed based on the results of FastQC and MultiQC. Trimmed reads were aligned to the rat reference genome with Star. To remove true PCR duplicates, the UMIs were used to deduplicate aligned reads with UMI-tools and the remaining reads per gene were counted implementing HTSeq. The following quality parameters were calculated in QoRTs and visualised in MultiQC reports (Supplements IX–XII) or figures (Supplement III, QC2 section): (i) percent genes with counts, (ii) cumulative gene assignment, (iii) percent reads in genes CDS and UTR, (iv) gene body coverage and (v) strandedness).

### Kits, reagents, equipment and software

Detailed information of the equipment and materials used is available in Supplement III.

### Data analysis

#### Principal component analysis plot

With the aim of identifying and excluding outlier samples, a pre-processing quality check was performed on sequencing raw data of all samples by visualising samples dispersion in a PCA plot (Supplement I, Figure S2). The PCA Plot was generated in R program version 4.3.0 (2023-04-21 ucrt). The DESeq function from the DESeq2 package was used to normalise the raw read counts after which VST and finally plotPCA functions were applied to produce the PCA plot.

#### Differential gene expression analysis

Differential gene expression analysis was performed using the RNAseqChef web-based application (v1.0.9; accessed September 6, 2023; available at: https://imeg-ku.shinyapps.io/RNAseqChef/). To ensure a robust and high-confidence identification of differentially expressed genes (DEGs), we employed a multi-step filtering strategy using three independent methods, namely DESeq2, EBseq, and edgeR.

**Figure 1.**
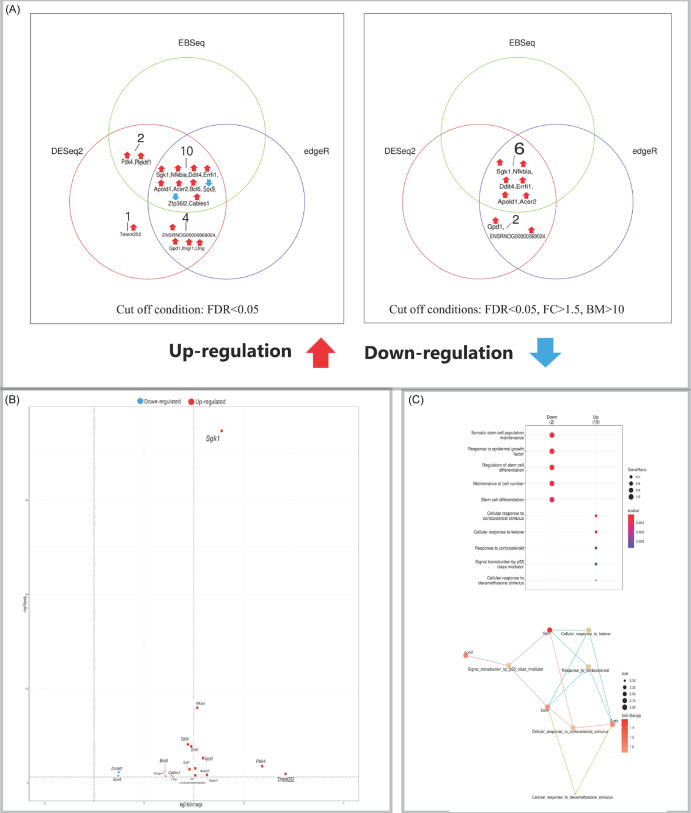
(A) Venn diagrams showing the number of DEGs in the hippocampus identified by three independent methods (DESeq2, edgeR, EBSeq) using an FDR < 0.05 (left panel), and the number of high-confidence DEGs remaining after applying additional filters (|FC| > 1.5, base mean > 10) to the results from each method (right panel). (B) Volcano plot visualising differential expression results from DESeq2 for the hippocampus. Significantly upregulated (red) and downregulated (blue) genes are labelled. (C) Gene Ontology (GO) enrichment analysis of DEGs. Top: dot plot of significantly enriched biological processes. Bottom: cnet plot visualising the relationships between the top enriched GO terms and the leading core genes associated with these. Input genes were the significant DEGs identified by DESeq2.

For each method, an initial list of candidate DEGs was generated based on an adjusted *p*-value (FDR) threshold of <0.05. To improve the reliability and possible biological relevance, two additional stringent cut-off conditions were then applied to the candidate lists from each method: 1. Fold Change > 1.5 (|log2(Fold Change)| > 0.585) to ensure a minimum magnitude of change. 2. Base Mean > 10 to filter out lowly expressed genes, which are more prone to technical noise and unreliable estimation. This stringent filtering process, as visualised in the A panels of Figure 1 through Figure 5, yielded a final set of high-confidence DEGs from each tool. The specific statistical framework for each method was as follows.

DESeq2: The analysis was performed using the standard DESeq2 workflow (Love *et al.*, 2014) which models raw count data using a negative binomial (NB) distribution and estimated dispersion for each gene. The Wald test was applied to test the significance of the comparison between experimental groups.

EBSeq: The analysis was conducted using the EBSeq framework (Leng *et al.*, 2013) which employs an empirical Bayesian approach under a NB model to calculate the posterior probability of a gene being differentially expressed.

edgeR: This analysis was performed using the edgeR package (Robinson *et al.*, 2010) which models count data using a negative binomial distribution. Gene-wise dispersions were estimated and squeezed towards a common value using an empirical Bayes method. Significance was tested using a Fisher’s exact test-like approach. The consensus final list of high-confidence DEGs was defined as the intersection of genes identified as significant by all three methods after applying the described filters (FDR < 0.05, |FC| > 1.5, Base Mean >10).

#### Volcano plots

The results of the differential expression analysis from the DESeq2 method were visualised using volcano plots generated within the RNAseqChef application. These plots provide a global overview of the expression changes by plotting the statistical significance – −log10(adjusted *p*-value) – against the magnitude of change (log2(fold change)) for every gene tested. This visualisation allowed for the immediate identification of genes with large fold changes and high statistical significance, effectively illustrating the impact of our applied statistical filters. The final high-confidence DEGs from the DESeq2 analysis meeting all criteria (FDR < 0.05, |FC| > 1.5, and Base Mean > 10) are highlighted in these plots.

#### Gene ontology (GO) enrichment analysis

GO enrichment analysis was performed based on an input DEG list generated by DESeq2 to identify significantly over-represented biological processes. The analysis was conducted using the clusterProfiler tool within the RNAseqChef application (Kan-E, 2022) which performs a statistical over-representation analysis based on a hypergeometric test. The background gene set for the enrichment test consisted of all genes that passed the initial expression filters in the RNA-seq dataset. *p*-values were adjusted for multiple testing using the Benjamini–Hochberg method and GO terms with an adjusted *p*-value (FDR) < 0.05 were considered significantly enriched.

## Results

The PCA plot obtained when assessing the transcriptional effects of a single dose of psilocybin (2 mg/kg) versus saline in the male rat brain by means of RNA sequencing on five regions displayed a clear separation between samples of distinct brain regions (Supplement I, Figure S2). Despite the lack of intra-regional clustering by treatment in the PCA, subsequent region-wise differential expression analysis identified a psilocybin-induced transcriptional response, the details of which are presented for each region below. Control analyses confirmed that differences between hemispheres did not significantly confound the results (Supplement I, Table S1).

### Hippocampus

Comparison of psilocybin- versus placebo-treated animals revealed 17 DEGs in the hippocampus (Figure 1A). Fifteen genes were upregulated, including several with established roles in synaptic plasticity and stress response, such as *Sgk1* (a glucocorticoid-responsive serine/threonine kinase), *Nfkbia* (an inhibitor of NF-κB signalling), and *Ddit4* (a regulator of mTOR activity). The upregulated gene *Apold1* is involved in vascular plasticity. Conversely, the downregulated genes included *Sox9*, a transcriptional regulator involved in cell fate, and *Zfp36l2* (also known as *Tis11d*), an RNA-binding protein that regulates mRNA stability. The expression changes for these DEGs are visualised in a volcano plot (Figure 1B). Pathway enrichment analysis further identified significant biological pathways associated with the psilocybin-induced expression profile (Figure 1C).

### Striatum

**Figure 2.**
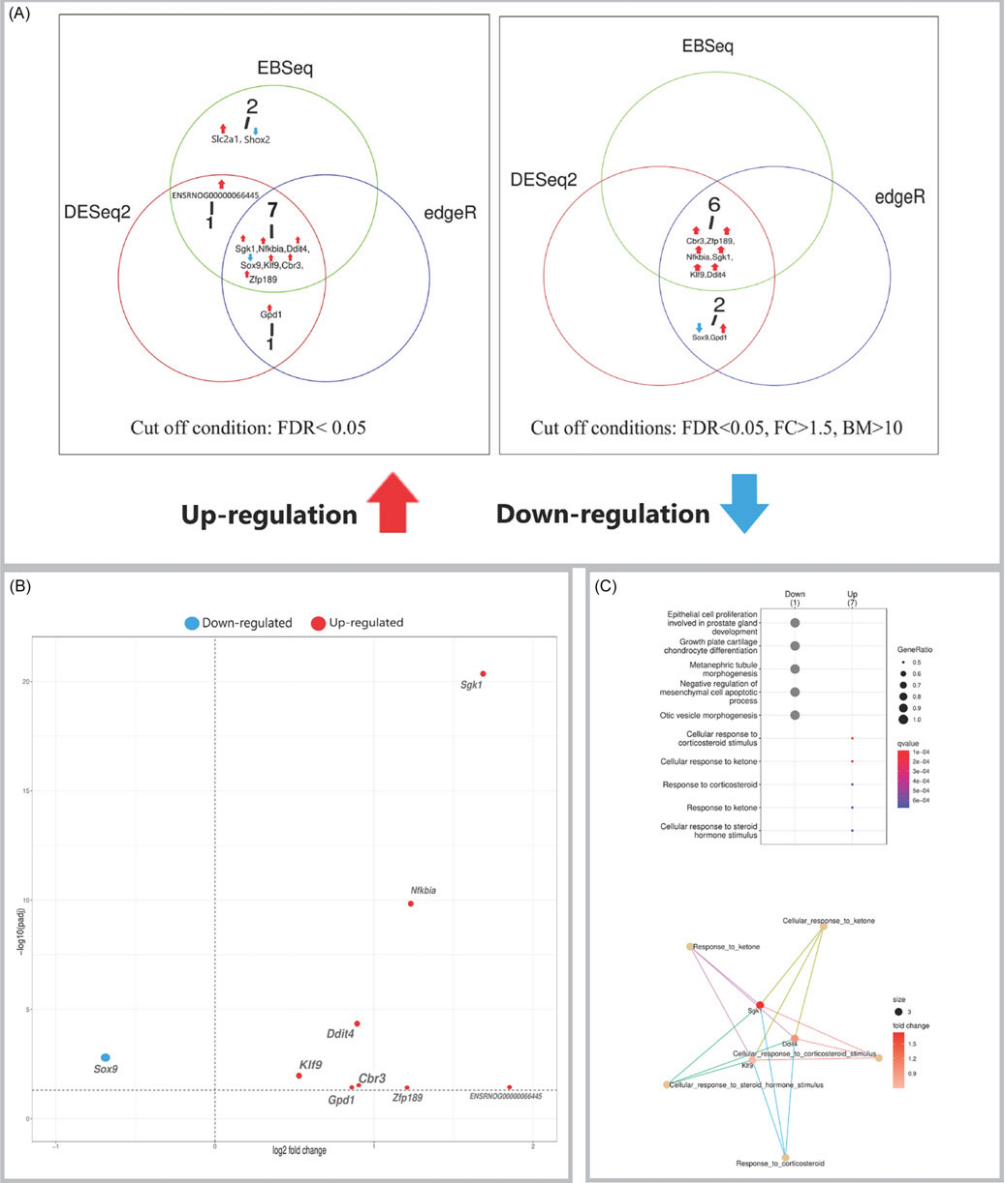
(A) Venn diagrams showing the number of DEGs identified in the striatum by three independent methods (DESeq2, edgeR, EBSeq) using an FDR < 0.05 (left panel), and the number of high-confidence DEGs remaining after applying additional filters (|FC| > 1.5, base mean > 10) to the results from each method (right panel). (B) Volcano plot visualising differential expression results from DESeq2 for the striatum. Significantly upregulated (red) and downregulated (blue) genes are labelled. (C) Gene Ontology (GO) enrichment analysis of DEGs. Top: dot plot of significantly enriched biological processes. Bottom: cnet plot visualising the relationships between the top enriched GO terms and the leading core genes associated with them. Input genes were the significant DEGs identified by DESeq2.

In the striatum, eleven DEGs were identified (Figure 2A). Nine genes showed upregulation, including the consistent plasticity- and stress-related markers *Sgk1*, *Nfkbia*, and *Ddit4*. This was contrasted with the downregulation of *Sox9*, a transcriptional suppressor of neurogenesis, and *Shox2*, a transcriptional controller of neuronal development. The corresponding volcano plot depicts these changes (Figure 2B), and pathway analysis revealed significantly enriched biological pathways (Figure 2C).

### Amygdala

**Figure 3.**
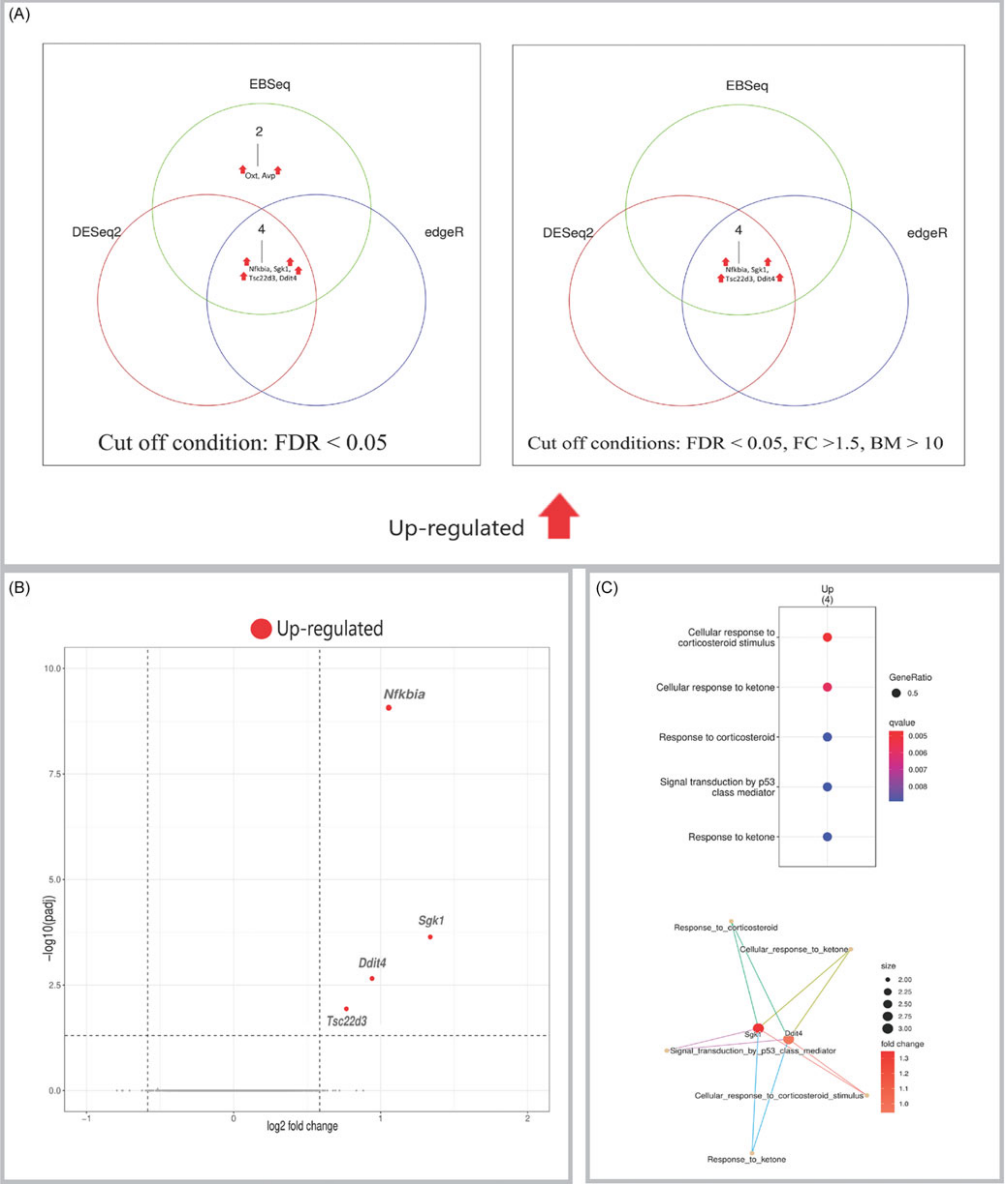
(A) Venn diagrams showing the number of DEGs identified in the amygdala by three independent methods (DESeq2, edgeR, EBSeq) using an FDR < 0.05 (left panel), and the number of high-confidence DEGs remaining after applying additional filters (|FC| > 1.5, base mean > 10) to the results from each method (right panel). (B) Volcano plot visualising differential expression results from DESeq2 for the amygdala. Significantly upregulated genes are shown in red and labelled. (C) Gene Ontology (GO) enrichment analysis of DEGs. Top: dot plot of significantly enriched biological processes. Bottom: cnet plot visualising the relationships between the top enriched GO terms and the leading core genes associated with them. Input genes were the significant DEGs identified by DESeq2.

Data analysis of the amygdala samples detected six genes that were upregulated by psilocybin (Figure 3A). These included core set of genes *Sgk1*, *Nfkbia*, and *Ddit4*, hence reinforcing their role as a common transcriptional response. Also upregulated were *Tsc22d3* (also known as *Gilz*, a glucocorticoid-responsive transcript), *Oxt* (which encodes the neuropeptide oxytocin), and *Avp* (which encodes arginine vasopressin (AVP)). The volcano plot illustrates the scale and significance of these expression differences (Figure 3B). Subsequent pathway enrichment analysis highlighted significantly enriched biological pathways (Figure 3C).

### Medial prefrontal cortex

**Figure 4.**
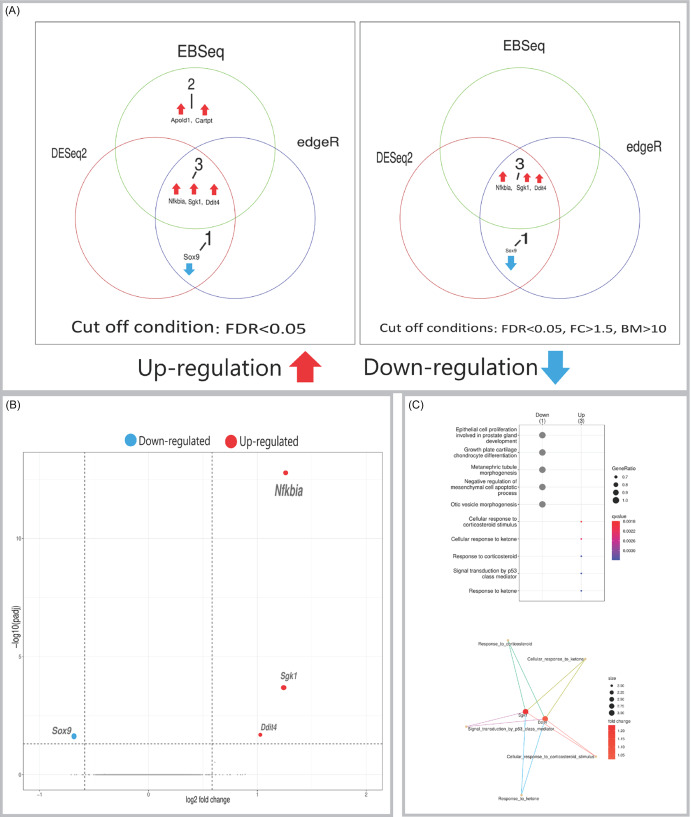
(A) Venn diagrams showing the number of DEGs identified in the medial prefrontal cortex by three independent methods (DESeq2, edgeR, EBSeq) using an FDR < 0.05 (left panel), and the number of high-confidence DEGs remaining after applying additional filters (|FC| > 1.5, base mean > 10) to the results from each method (right panel). (B) Volcano plot visualising differential expression results from DESeq2 for the medial prefrontal cortex. Significantly upregulated (red) and downregulated (blue) genes are labelled. (C) Gene Ontology (GO) enrichment analysis of DEGs. Top: dot plot of significantly enriched biological processes. Bottom: cnet plot visualising the relationships between the top enriched GO terms and the leading core genes associated with them. Input genes were the significant DEGs identified by DESeq2.

Six DEGs were found in the medial prefrontal cortex (Figure 4A). Five genes were upregulated, including the conserved trio *Sgk1*, *Nfkbia*, and *Ddit4*, alongside *Apold1*. Notably, *Cartpt* (which encodes Cocaine- and Amphetamine-Regulated Transcript peptide), a modulator of dopaminergic neurotransmission, was also upregulated. As in other regions, *Sox9* was downregulated. These results are detailed in the volcano plot (Figure 4B), with pathway enrichment identifying significantly enriched biological processes (Figure 4C).

### Cingulate cortex

**Figure 5.**
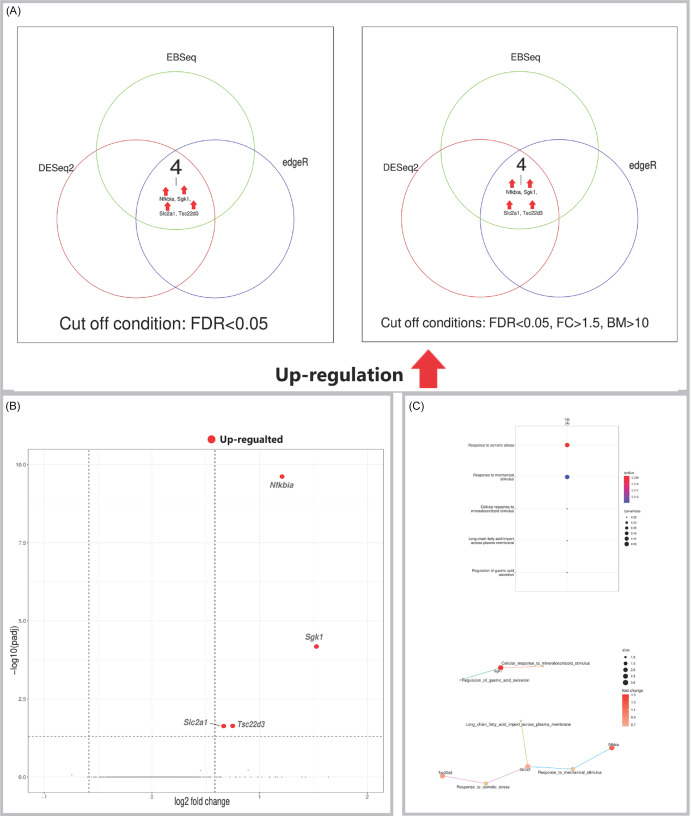
(A) Venn diagrams showing the number of DEGs identified in the cingulate cortex by three independent methods (DESeq2, edgeR, EBSeq) using an FDR < 0.05 (left panel), and the number of high-confidence DEGs remaining after applying additional filters (|FC| > 1.5, base mean > 10) to the results from each method (right panel). (B) Volcano plot visualising differential expression results from DESeq2 for the cingulate cortex. Significantly upregulated genes are shown in red and labelled. (C) Gene Ontology (GO) enrichment analysis of DEGs. Top: dot plot of significantly enriched biological processes. Bottom: cnet plot visualising the relationships between the top enriched GO terms and the leading core genes associated with them. Input genes were the significant DEGs identified by DESeq2.

The cingulate cortex exhibited four DEGs, all upregulated (Figure 5A). Changes included the upregulation of *Sgk1*, *Nfkbia*, *Ddit4*, and *Tsc22d3* (*Gilz*), hence suggesting a shared transcriptional signature with the amygdala. The magnitude and significance of these changes are illustrated in the volcano plot (Figure 5B). Furthermore, significantly enriched biological pathways induced by psilocybin in cingulate cortex are shown in Figure 5C.

### Comparison of regions

**Figure 6.**
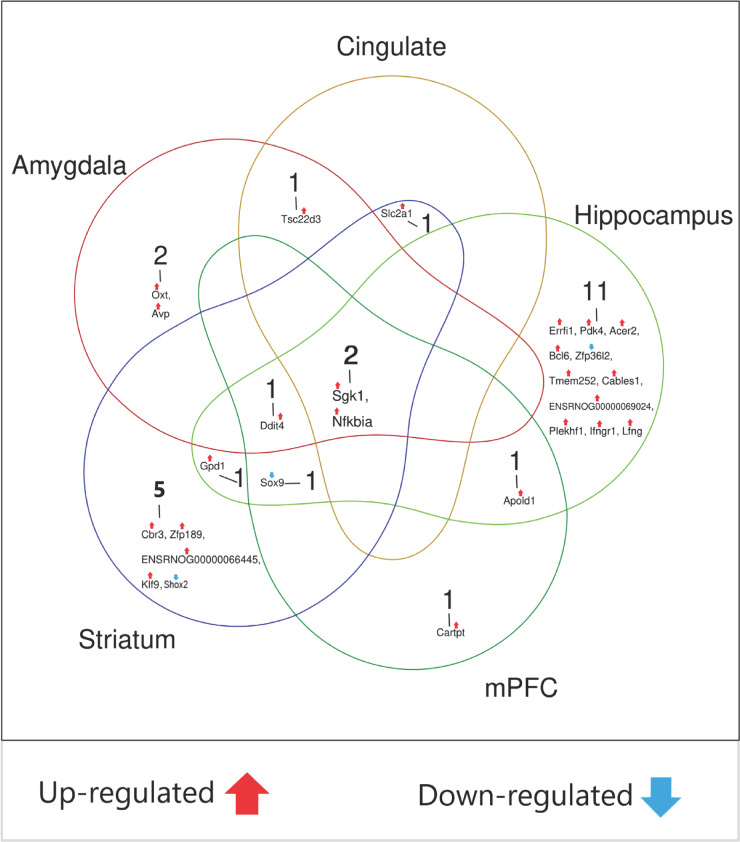
The number of overlapping and unique DEGs (FDR < 0.05) across brain regions. A gene was considered differentially expressed if called by any of the three methods (DESeq2, edgeR, or EBSeq).

A comparative analysis revealed a conserved transcriptional response to psilocybin across different brain areas (Figure 6). The most significant finding was the consistent upregulation of *Sgk1* and *Nfkbia* in all five brain regions and of *Ddit4* in four. Downregulation of *Sox9* was observed in the hippocampus, striatum, and medial prefrontal cortex. Other notable changes included the upregulation of *Apold1* in the hippocampus and medial prefrontal cortex and of *Tsc22d3* in the amygdala and cingulate cortex. Region-specific changes, such as the upregulation of the neuropeptides *Oxt* and *Avp* in the amygdala and *Cartpt* in the medial prefrontal cortex, suggest potential region-specific functional outcomes.

## Discussion

This study provides, to our knowledge, the first unbiased multi-region transcriptional analysis of the acute effects of psilocybin in the brain. PCA revealed that the dominant source of transcriptional variation was biological region of origin, with minimal discernible influence from the divergent processing protocols used for the hippocampus compared to the other four regions. As evidenced by the lack of intra-regional sample separation in the PCA, our more sensitive, region-wise differential expression analysis affirmed that only a targeted subset of genes was differentially expressed. Analysis of these DEGs revealed two key findings: first, we provide a foundational dataset that reveals a highly conserved core of cross-regional transcriptional changes (*Sgk1*, *Nfkbia*, and *Ddit4*), partially conserved alterations shared between specific regions (*Gpd1*, *Apold1*, *Sox9*, *Tsc22d3*, *Slc2a1*), and a limited number of region-specific alterations. Second, we report that a high proportion of psilocybin-induced DEGs are established glucocorticoid-responsive genes across all the studied regions (Figure 6, Table S4). This convergence suggests that activation of glucocorticoid-signalling pathways may represent a novel mechanistic axis for psilocybin’s acute transcriptional and potential antidepressant effects.

Foremost among these highly conserved, cross-regional changes was the universal upregulation of the serine/threonine kinase *Sgk1*. This finding significantly expands upon previous work that identified *Sgk1* induction as a key response to psychedelics; a targeted study of 46 predefined genes thus demonstrated a marked increase in *Sgk1* expression in the rat hippocampus and prefrontal cortex following psilocybin (Jefsen *et al.*, 2021), and enhanced expression of this gene was also reported in the hippocampus, midbrain, and prefrontal cortex after administration of LSD (Nichols & Sander-Bush, 2002). Our untargeted approach now definitively establishes that this is not a limited or region-dependent phenomenon but a conserved transcriptional response to psilocybin across multiple brain regions (Figure 6).

The widespread upregulation of *Sgk1* is significant given its critical role in neuroplasticity. *Sgk1* is reported to facilitate the expression of long-term potentiation in hippocampal neurons (Ma *et al.*, 2006) and may impact neuronal activity by regulating ion channels, carriers, Na+/K+-ATPase, enzymes, and transcription factors (e.g. Foxo3a, *β*-catenin, and Nuclear factor κB (NF-κB)) (Lang *et al.*, 2010). Its involvement in stress response pathways and depression (Anacker *et al.*, 2013) is particularly relevant, as elevated *Sgk1* expression is a common feature of diverse antidepressant treatments, including ketamine (Ficek *et al.*, 2016; Wegman-Points *et al.*, 2020), electroconvulsive therapy (Conti *et al.*, 2007), and serotonin reuptake inhibition (Conti *et al.*, 2007). This convergence suggests *Sgk1* activation may be a fundamental mechanism underpinning antidepressant effects. Likewise, a previous study (Hussain *et al.*, 2015) assessing the impact of the tricyclic antidepressant imipramine – administered in combination with an alpha_2_-receptor antagonist – showed enhanced expression of both *Sgk1* and two other genes which were also found to be upregulated by psilocybin in the present study – *Nfkbia and Acer2.* For a comparison of the transcriptional effects of psilocybin relative to those of other antidepressant treatments, see Supplement I (Table S5).

The enhanced expression of *Nfkbia* following psilocybin is in line with the previous reports regarding psilocybin (Jefsen *et al.*, 2021) and LSD (Nichols & Sander-Bush, 2002), and such a response has also been observed following serotonin reuptake inhibitors and electroconvulsive treatment (Conti *et al.*, 2007). The expression of *Nfkbia* is stimulated by *NF-κB* proteins which in turn are inactivated by *Nfkfbia* (Brown *et al.*, 1993). A likely mechanism for the up-regulation of *Nfkbia* would be that psilocybin has activated the NF-κB complex, which – like *Sgk1 –* is also activated in the context of long-term potentiation (Meberg *et al.*, 1996). NF-κB is also reported to be involved in a wide range of neuronal processes including neuroplasticity (Mattson & Camandola, 2001) and has been implicated in depression and the antidepressant effect of ketamine (Sokolowska et al., 2023).

Up-regulation of *Ddit4* (*REDD1*) was also observed in all the studied regions; in cingulate cortex, this difference was however significant only according to the Wilcoxon test. *Ddit4* suppresses mammalian target of rapamycin complex 1 (*mTORC1*) and regulates cell growth (Sofer *et al.*, 2005). Up-regulation of *Ddit4* may tentatively be the result of a negative feedback response following activation of the *mTOR* signalling pathway induced by psilocybin. It has been evidenced that *mTOR* activation plays a key role in structural plasticity (Jaworski *et al.*, 2005; Kumar *et al.*, 2005), production of proteins necessary for synaptogenesis (Hoeffer & Klann, 2010), the plasticity-promoting effects of classical serotonergic psychedelics (Ly *et al.*, 2018), and some behavioural effects of ketamine (Dwyer & Duman, 2013).

*Sox9* was significantly downregulated in hippocampus, striatum, and medial prefrontal cortex (Figure 6). Several studies indicate that *Sox9* plays an important role in the maintenance and multipotentiality of neural stem cells during development (Scott *et al.*, 2010). Conversely, overexpression of *Sox9* during spinal cord development reduces the number of neuronal progenitors and neurons (Vogel *et al.*, 2020). Its roles are, however, not limited to development since SOX proteins influence survival, regeneration, and cell death and control homeostasis also in adult tissues (Stevanovic *et al.*, 2021). It has been shown that knockdown of *miR-124*, which targets *Sox9*, increases *Sox9* expression, which in turn decreases neurogenesis in the adult mouse brain (Stevanovic *et al.*, 2021); it is hence not inconceivable that psilocybin may promote various forms of neuroplasticity by down-regulating *Sox9* and another member of the *Sox* gene family, *Sox2*; the latter was – according to the Wilcoxon test – downregulated in hippocampus only (Supplement I, Figure S5) (Mercurio *et al.*, 2019; Lima de Cruz et al., 2024).

*Apold1* was upregulated both in hippocampus and medial prefrontal cortex (Figure 6). This immediate early gene, which is expressed selectively in vascular endothelial cells – and also known as *Verge* (vascular early response gene) – is involved in angiogenesis (Font *et al.*, 2010). Of note it that *Verge* has previously been reported to be a target gene for BDNF, a protein playing a key role for neuroplasticity, and implicated in the mechanism of different types of antidepressants (Koshimizu *et al.*, 2021).

It was also found that *Gpd1* is upregulated by psilocybin in both hippocampus and striatum. The possible function of *Gpd1* in brain remains to be uncovered. The glucose transporter *Slc2a1*, which was upregulated in striatum and cingulate cortex, has previously been shown to be upregulated also by ketamine (Wegman-Points *et al.*, 2020). *Zfp189*, which was upregulated in striatum, is a zinc finger protein previously suggested to be involved in stress resilience (Lorsch *et al.*, 2019). *Klf9*, which was also upregulated in striatum, has been suggested to exert a protective impact on dopaminergic neurons (Parga *et al.*, 2018).

Of note is that not only *Sgk1*, but most of the psilocybin-induced DEGs in this study, are genes whose transcription in brain has been shown to be regulated by glucocorticoids such as cortisol, corticosterone, and dexamethasone (Juszczak & Stankiewicz, 2018) – thus, 10/17 in hippocampus, 6/10 in striatum, 4/6 in medial prefrontal cortex, 4/6 in amygdala, and 3/4 in cingulate cortex are established glucocorticoid-responsive genes (Figure 6, and Table S4 in Supplement I). This convergence, also confirmed by GO enrichment analysis (C panels in Figure 1 through Figure 5), raises the possibility of a novel mechanism: that a significant portion of psilocybin’s acute transcriptional effects may be mediated through the engagement of a stress response and glucocorticoid-signalling pathways. This could hence provide a unifying explanation for why psilocybin, stress hormones, and diverse antidepressants (ketamine, ECT, SSRIs (Conti *et al.*, 2007; Anacker *et al.*, 2013; Ficek *et al.*, 2016; Wegman-Points *et al.*, 2020) converge on similar plasticity genes like *Sgk1* and *Nfkbia*. Notably, it has been reported that glucocorticoids are released following the administration of psilocybin and many other hallucinogens both in humans (Hasler *et al.*, 2004; Schindler *et al.*, 2018) and mice (Jones *et al.*, 2023), and some data suggest that this effect may contribute to the anxiolytic-like effect of the substance (Anacker *et al.*, 2013).

Another finding of potential importance was that psilocybin – according to Wilcoxon statistics – enhanced the expression of the *Cartpt* mRNA in medial prefrontal cortex (Supplement I, Figure S13). This gene encodes a peptide, CART, which has been portrayed as an endogenous amphetamine because of its impact on dopaminergic transmission (Rogge *et al.*, 2008). While a previous study has reported enhanced *Cartpt* expression following administration of different antidepressants, including ketamine (Funayama *et al.*, 2022), an impact of psilocybin on CART has to our knowledge not been previously reported.

Given that psilocybin in all likelihood exerts its effects at least partly by activating serotonergic 5-HT2A receptors, and has also been shown to impact the activity of other major neurotransmitters (Sakashita *et al.*, 2015; Wojtas *et al.*, 2022; Wojtas *et al.*, 2023), it is noteworthy that the drug was not found to alter the expression of genes encoding proteins specifically implicated in the regulation of the synaptic activity of specific neurotransmitters, such as glutamate, and that we also observed no impact on the expression of 5-HT2A receptors or other postsynaptic serotonin receptors. It should however be noted that brain stem areas including monoaminergic cell bodies were not analysed. A transmitter-related observation of potential interest, however, was that psilocybin enhanced mRNA levels encoding two related neuropeptide transmitters, AVP and oxytocin neurophysin I prepropeptide, in the amygdala. Whereas of tentative importance given the suggested role for these peptides in the regulation of relevant aspects of behaviour, the observation regarding oxytocin should be interpreted with caution since oxytocinergic cell bodies are not expected to be present in the amygdala. AVP cell bodies, however, do exist not only in the hypothalamus, but also in the amygdala (Wang & De Vries, 1995), so the apparent increase in *Oxt* expression may be an artefact caused by the structural similarity between the two neuropeptides. The hypothalamus was not included in this study.

Several limitations of this study should be noted. First, we measured gene expression but not the levels of the proteins encoded by the identified DEGs. Second, the use of bulk RNA-seq means that we cannot attribute the observed transcriptional changes to specific cell types; future single-cell or single-nucleus studies will hence be crucial to resolve their cellular origin. Third, our findings are based on a single time point; investigating the dynamics of this response across different time points, and following blockade of serotonergic receptors, will be important to fully understand the dynamics and mechanistic underpinnings of this conserved transcriptional response. Fourth, this study was conducted exclusively in male rats to establish a foundational transcriptional profile since the oestrous cycle in females is known to impact 5-HT2A receptor expression (Summer & Fink, 1995; Summer & Fink, 1997; Zylko *et al.*, 2025; Cohen & Blest-Hopley, 2025) – including female rats would hence have required expanding the study to include animals at different phases of the reproductive cycle which was beyond the scope of this project. Given the well-established impact of sex and sex steroids on serotonergic activity, addressing the impact of psilocybin on gene transcription in females is, however, clearly warranted. Fifth, hippocampal samples were processed separately, which introduced minor technical differences; however, PCA confirmed that these changes were negligible and did not drive the primary results.

Taken together, the present study provides a foundational transcriptional dataset for psilocybin and suggests that its immediate impact on gene expression in five brain regions innervated by serotonin in the male rat is modest in number but highly consistent and biologically significant. We demonstrate that the largest number of DEGs was found in the hippocampus, and that no marked effects were observed on genes specifically regulating the activity of different neurotransmitters. A key finding is the discovery of a conserved glucocorticoid-psilocybin transcriptional axis, wherein many of the impacted genes are involved in the regulation of neuroplasticity and are also known to be regulated by glucocorticoids. This represents a novel and potentially fundamental mechanism that warrants further investigation.

## Supporting information

10.1017/neu.2026.10075.sm001 Veysi et al. supplementary material 1 Veysi et al. supplementary material

10.1017/neu.2026.10075.sm002 Veysi et al. supplementary material 2 Veysi et al. supplementary material

10.1017/neu.2026.10075.sm003 Veysi et al. supplementary material 3 Veysi et al. supplementary material

10.1017/neu.2026.10075.sm004 Veysi et al. supplementary material 4 Veysi et al. supplementary material

10.1017/neu.2026.10075.sm005 Veysi et al. supplementary material 5 Veysi et al. supplementary material

10.1017/neu.2026.10075.sm006 Veysi et al. supplementary material 6 Veysi et al. supplementary material

10.1017/neu.2026.10075.sm007 Veysi et al. supplementary material 7 Veysi et al. supplementary material

10.1017/neu.2026.10075.sm008 Veysi et al. supplementary material 8 Veysi et al. supplementary material

10.1017/neu.2026.10075.sm009 Veysi et al. supplementary material 9 Veysi et al. supplementary material

10.1017/neu.2026.10075.sm010 Veysi et al. supplementary material 10 Veysi et al. supplementary material

10.1017/neu.2026.10075.sm011 Veysi et al. supplementary material 11 Veysi et al. supplementary material

10.1017/neu.2026.10075.sm012 Veysi et al. supplementary material 12 Veysi et al. supplementary material

## Supplementary material

The supplementary material for this article can be found at https://doi.org/10.1017/neu.2026.10075.
