# Supplementary material for: Psilocybin elicits a conserved glucocorticoid-responsive gene signature across five 5-HT2A receptor-rich brain regions in rat
Source: Acta Neuropsychiatr. 2026 Apr 10;38:e37. doi: 10.1017/neu.2026.10075 (PMC13202413; doi:10.1017/neu.2026.10075)
Supplement: Veysi et al. supplementary material 1 — Veysi et al. supplementary material [file S0924270826100751sup001.pdf]

Supplement I

Psilocybin Elicits a Conserved Glucocorticoid-Responsive Gene Signature Across Five 5-HT2A Receptor-Rich Brain Regions in the Male Rat

Contents

Design of the experiment 1
PCA plot 2
Plots generated based on hippocampus samples' data 3
Plots generated based on striatum samples' data 7
Plots generated based on mPFC samples' data 11
Plots generated based on cingulate cortex samples' data 15
Plots generated based on amygdala samples' data 19
Tables 23
References 27

Design of the experiment

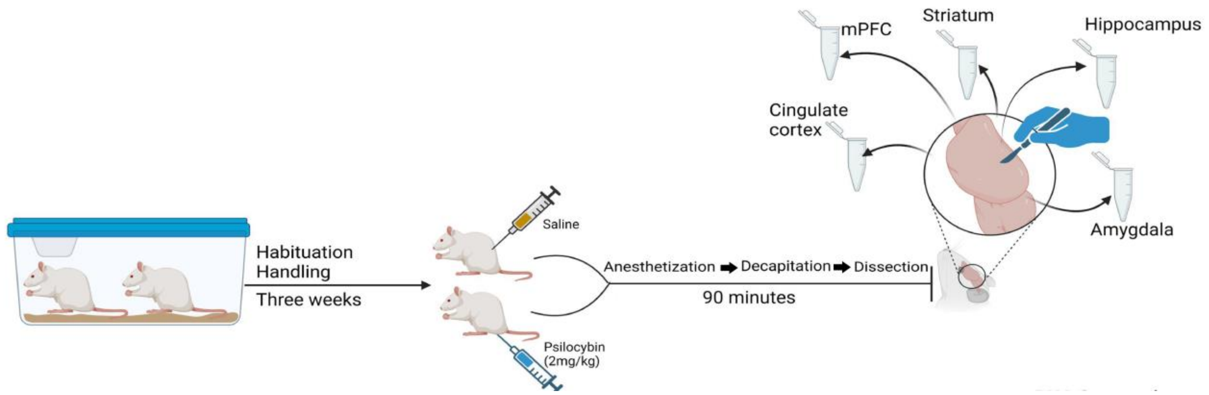

Figure S1 Design of the experiment.
Ninety minutes after psilocybin or saline administration subcutaneously, animals were anesthetized by administering a lethal dose of sodium pentobarbital (150 mg/kg) intraperitoneally, then subjected to decapitation, brain collection, and dissection of tissues of interest.

PCA plot

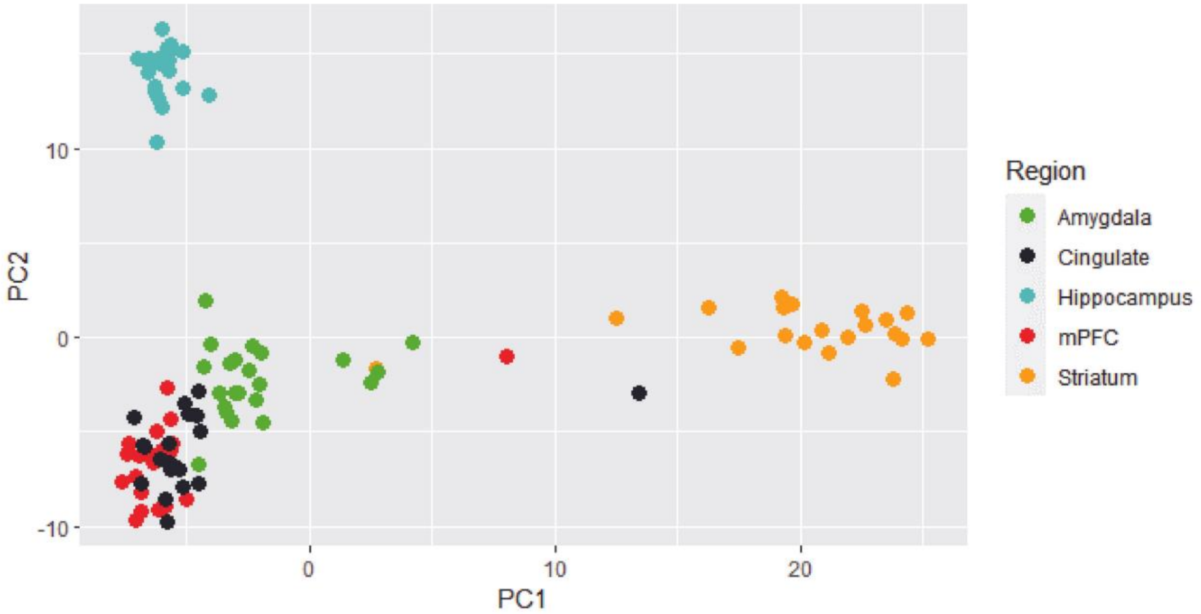

Figure S2 PCA plot depicting the first two components of the principal component analysis of the normalized counts of all samples with the aim of detecting outlier samples of each region.

Plots generated based on hippocampus samples' data

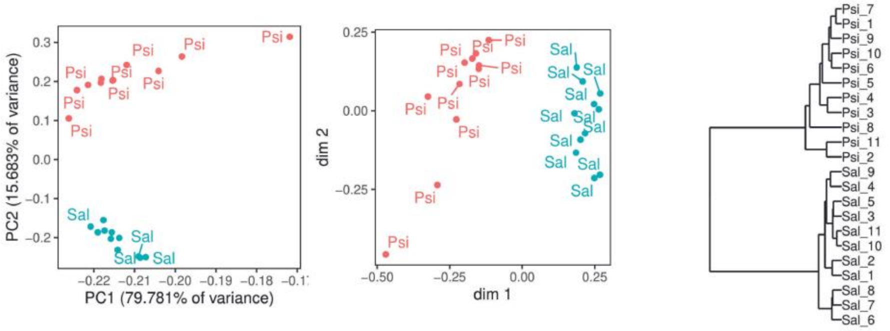

Figure S3 Three types of clustering analysis, including principal component analysis (PCA) (left panel), multidimensional scaling (MDS) (middle panel), and hierarchical clustering with the ward.D2 (right panel), performed based on DESeq2 normalized count table generated based on hippocampus samples' data and DEGs with FDR<0.05.

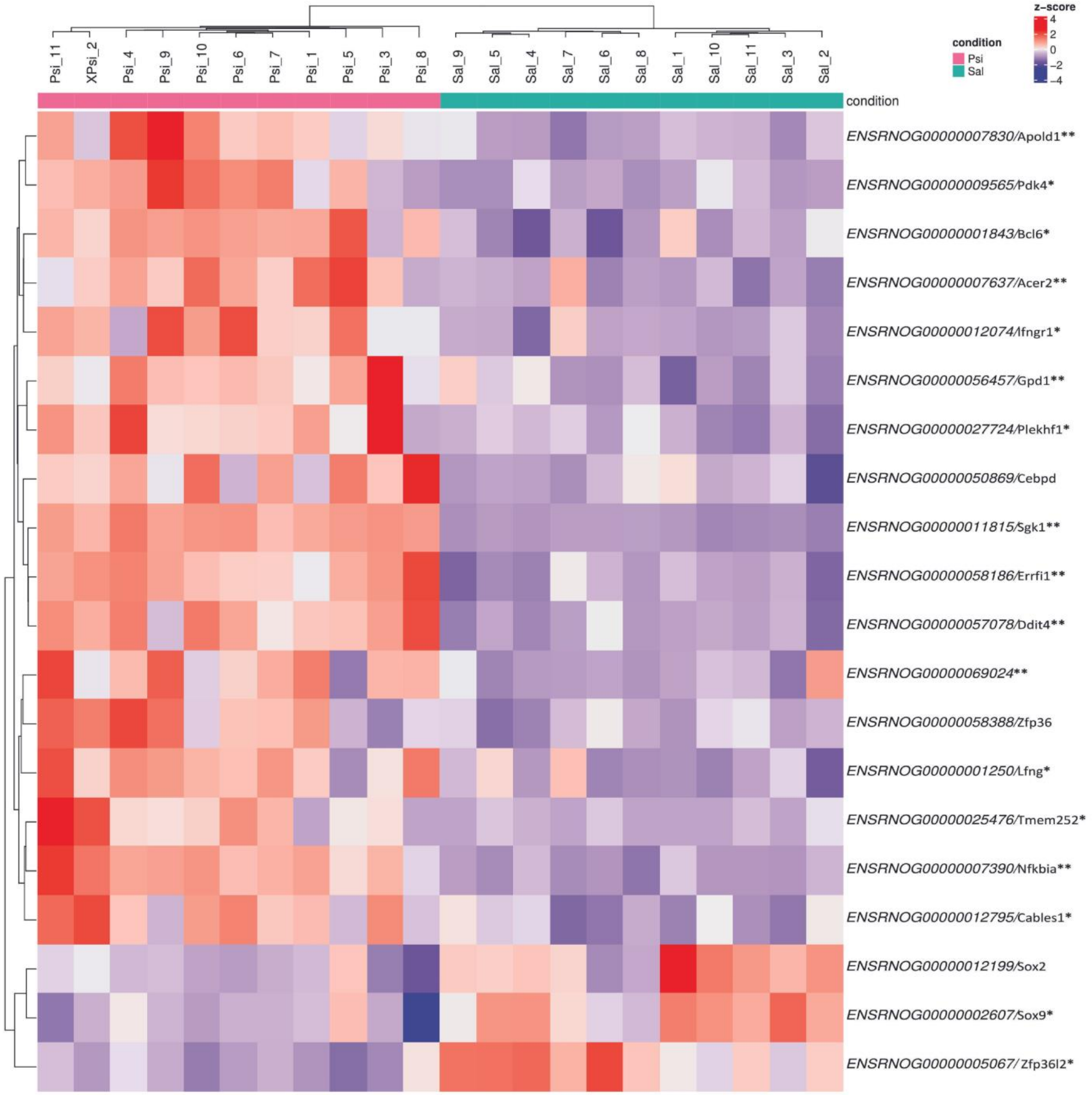

Figure S4 Heatmap of top 20 genes with lowest FDR extracted from DESeq2 result file generated based on hippocampus samples' data.

Genes with two asterisk are DEGs identified by the all the three data analysis methods, namely DESeq2, edgeR and EBSeq, but those with one asterisk are DEGs only identified by one or two, but not all the three methods.

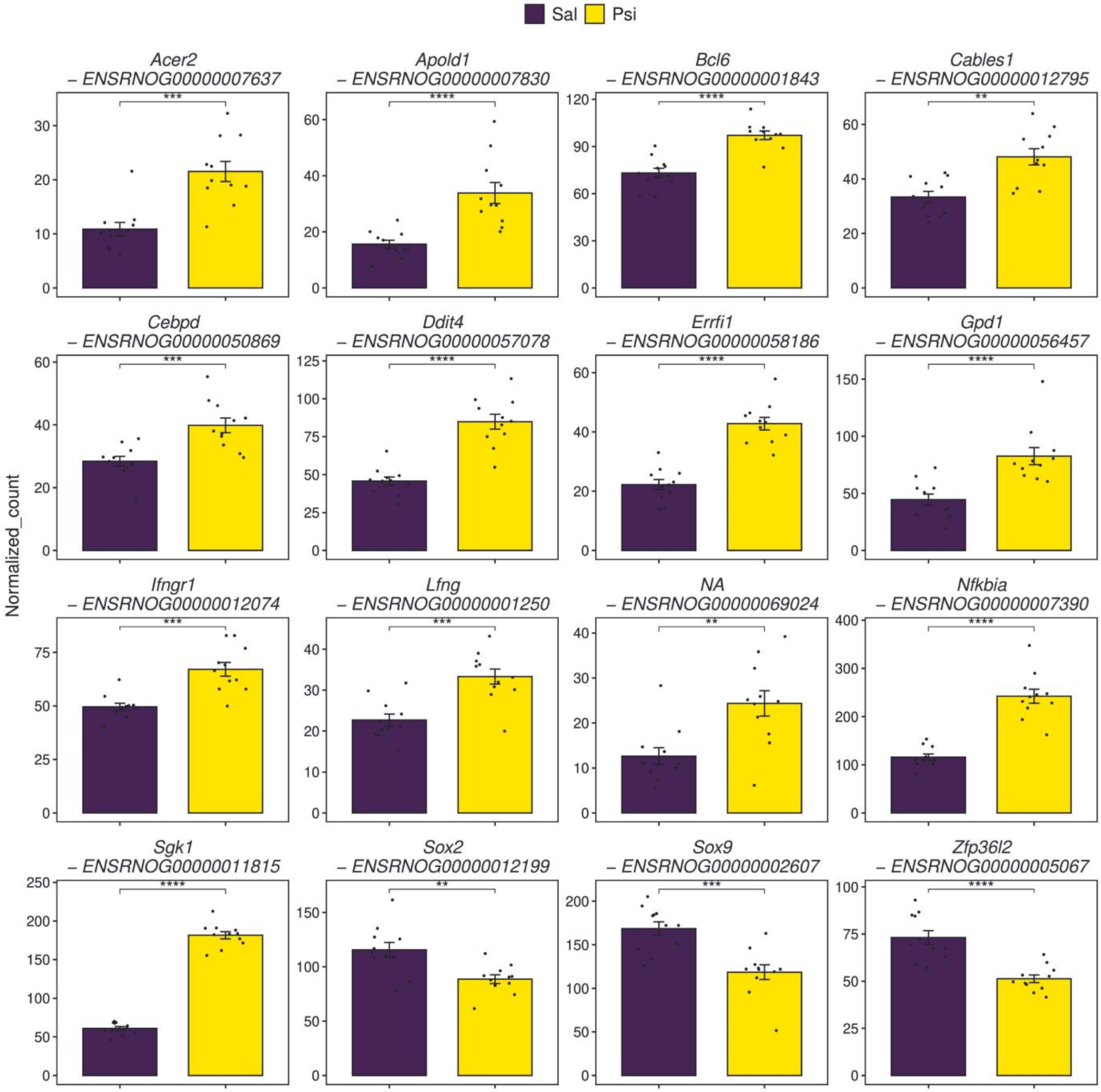

Figure S5 Bar-plot of DEGs with FDR<0.05, generated from DESeq2 normalized count table of hippocampus samples' data. Wilcoxon test has been applied to show fold change significance between two groups of samples for each DEG: the higher number of stars above pair bars of each DEG, the greater fold change.

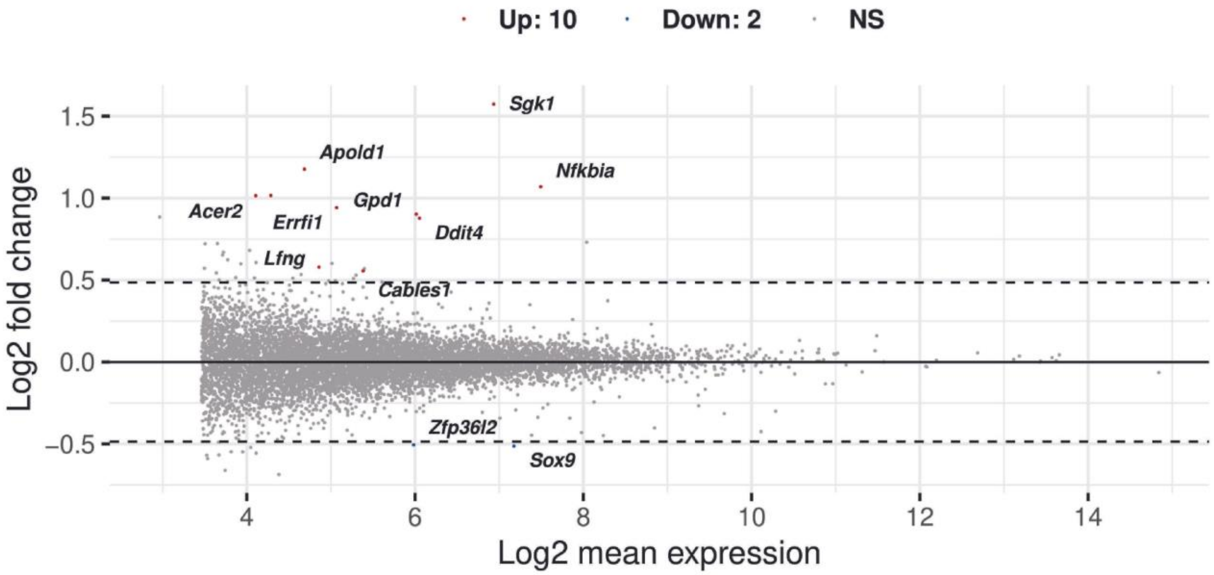

Figure S6 MA-plot of hippocampus *samples' data based on DEseq2 result file. Three types of thresholds were set for this plot: FDR < 0.05, FC > 1.4 and BM>10.*

Plots generated based on striatum samples' data

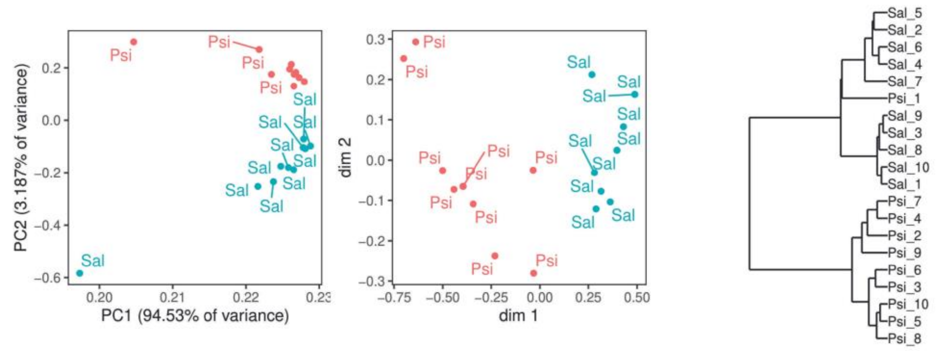

Figure S7 Three types of clustering analyses, including principal component analysis (PCA) (left panel), multidimensional scaling (MDS) (middle panel), and hierarchical clustering with the ward.D2 (right panel), performed based on DESeq2 normalized count table of striatum samples and DEGs with FDR<0.05.

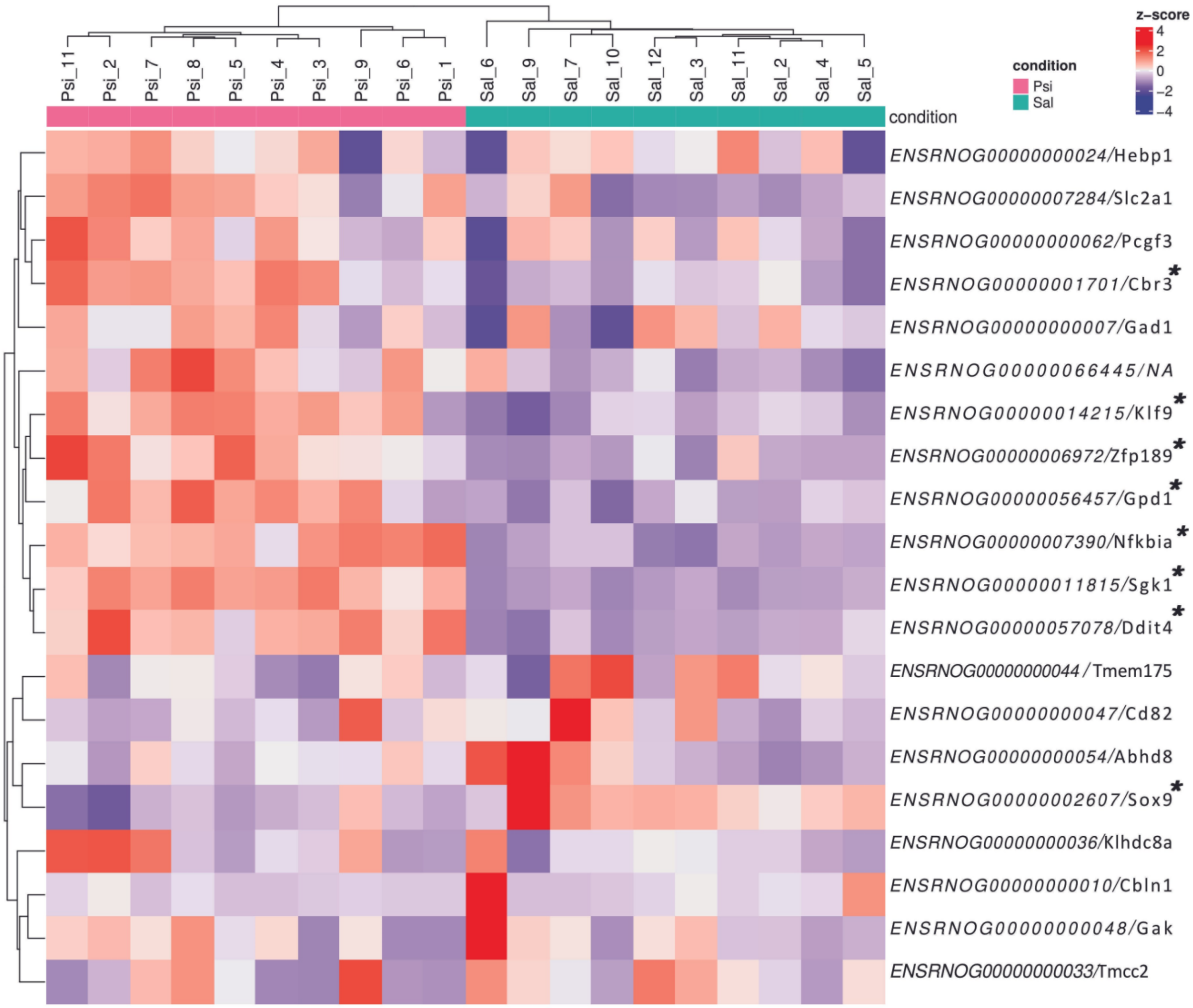

Figure S8 Heatmap of top 20 genes with lowest FDR extracted from DESeq2 result file generated based on striatum samples' data. Genes with asterisk are DEGs identified by the all the methods, namely DESeq2, edgeR and EBSeq.

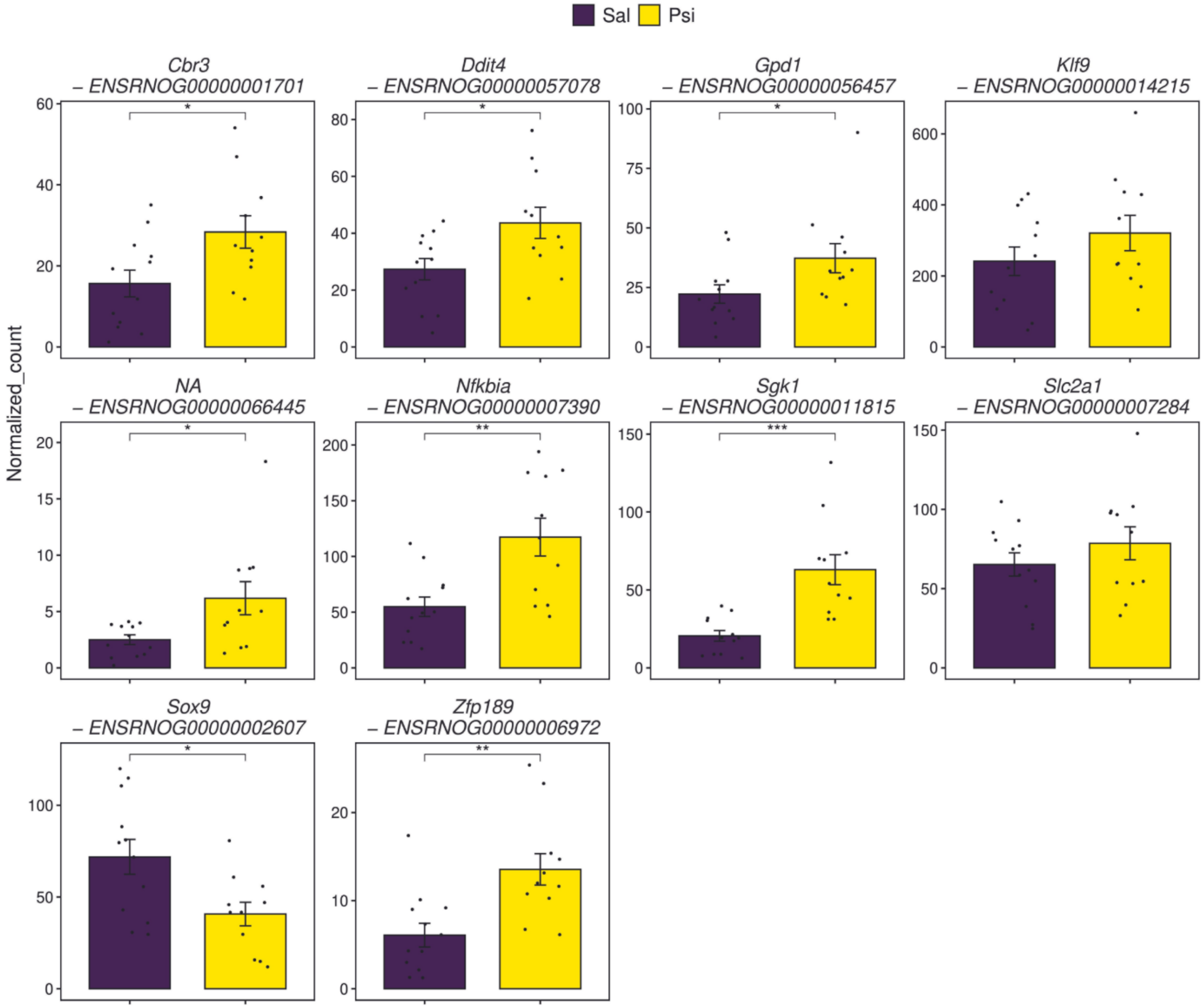

Figure S9 Bar-plot of DEGs with FDR<0.05 generated from DESeq2 normalized count table of striatum samples' data. Wilcoxon test has been applied to show fold change significance between two groups of samples for each DEG: the higher number of stars above pair bars of each DEG, the greater fold change.

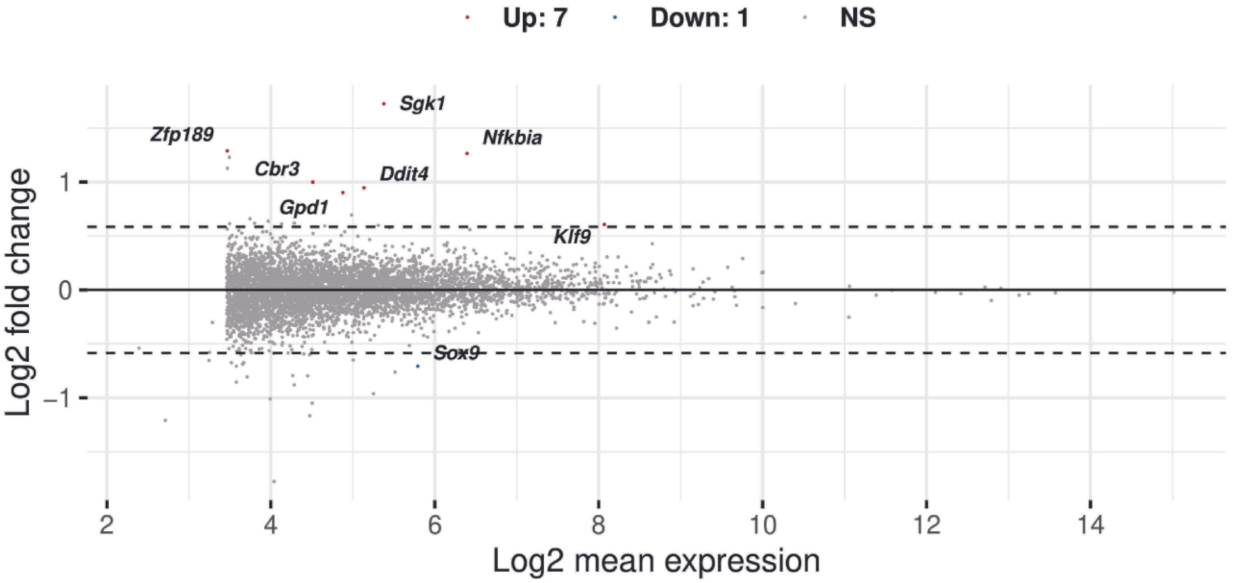

Figure S10 MA-plot based on DEseq2 result file of striatum samples' data. Three types of thresholds set for generating this plot include FDR < 0.05, FC > 1.5, and BM>10.

Plots generated based on mPFC samples' data

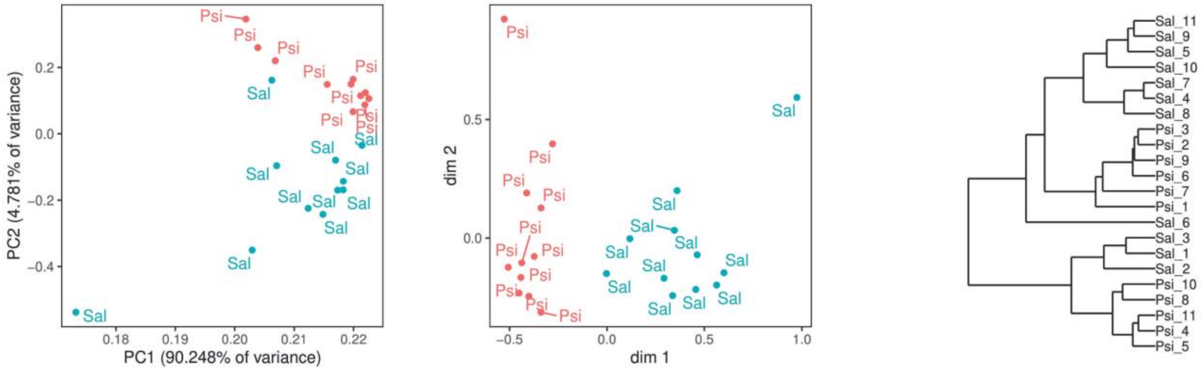

Figure S11 Three types of clustering analyses, including principal component analysis (PCA) (left panel), multidimensional scaling (MDS) (middle panel), and hierarchical clustering with the ward.D2 (right panel), performed based on DESeq2 normalized count table of mPFC samples and DEGs with FDR<0.05.

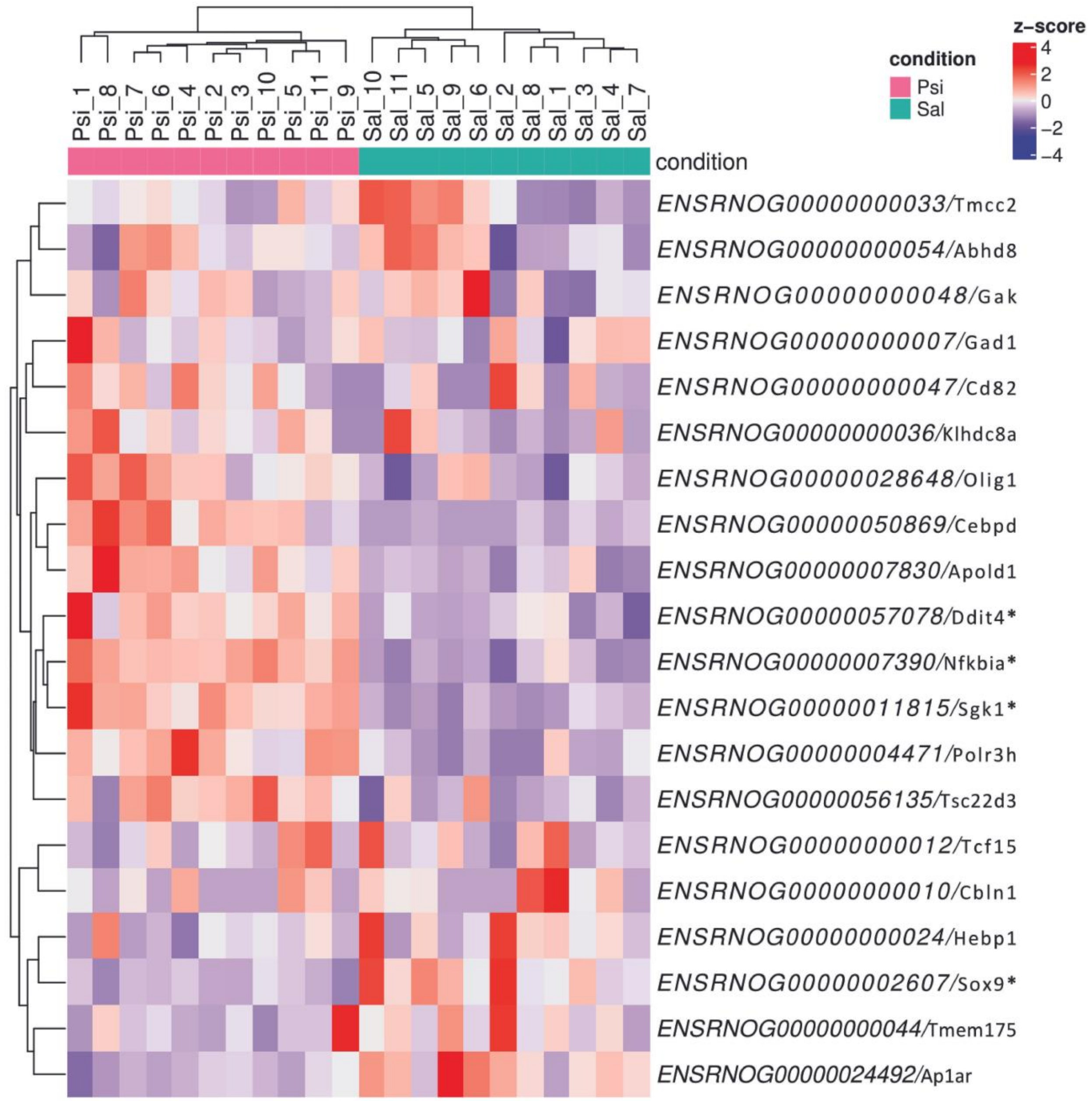

Figure S12 Heatmap of top 20 genes with lowest FDR extracted from DESeq2 result file generated based on mPFC samples' data from DESeq2 result file. Genes with asterisk are identified DEGs with FDR < 0.05 from the DEseq2 result file.

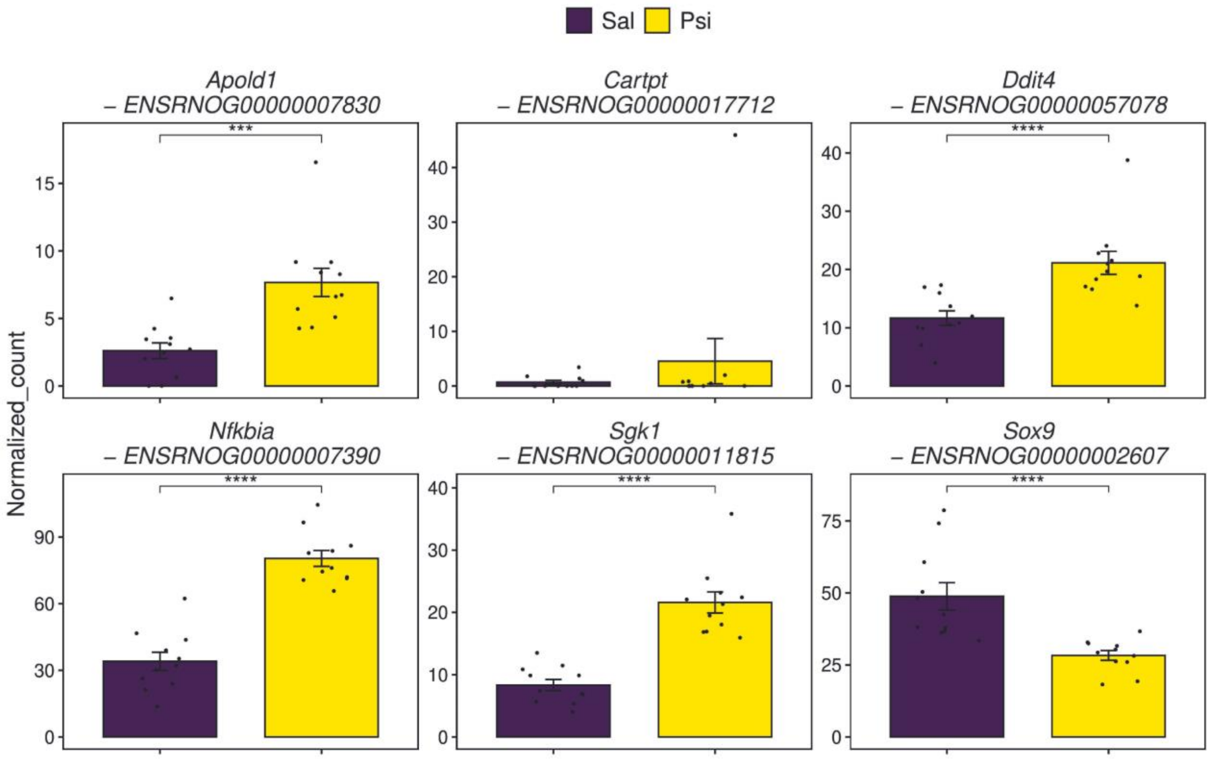

Figure S13 Bar-plot of DEGs with FDR<0.05 generated from DESeq2 normalized count table of mPFC samples' data. Wilcoxon test has been applied to show fold change significance between two groups of samples for each DEG: the higher number of stars above pair bars of each DEG, the greater fold change.

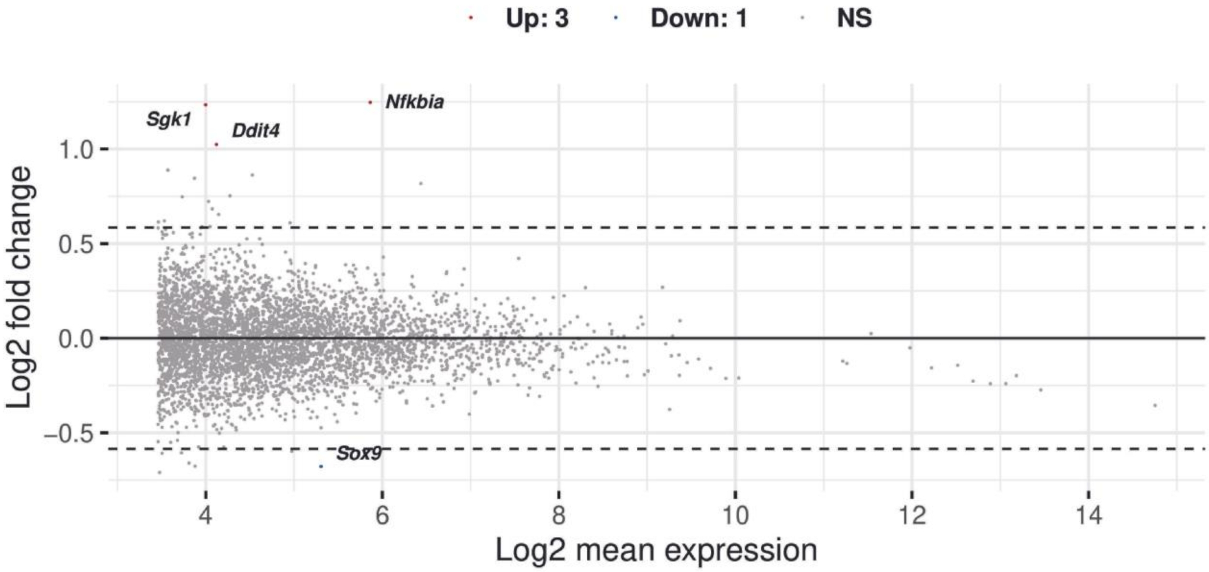

Figure S14 MA-plot based on DEseq2 result file of mPFC samples' data. Three types of thresholds set for generating this plot include FDR < 0.05, FC > 1.5, and BM>10.

Plots generated based on cingulate cortex samples' data

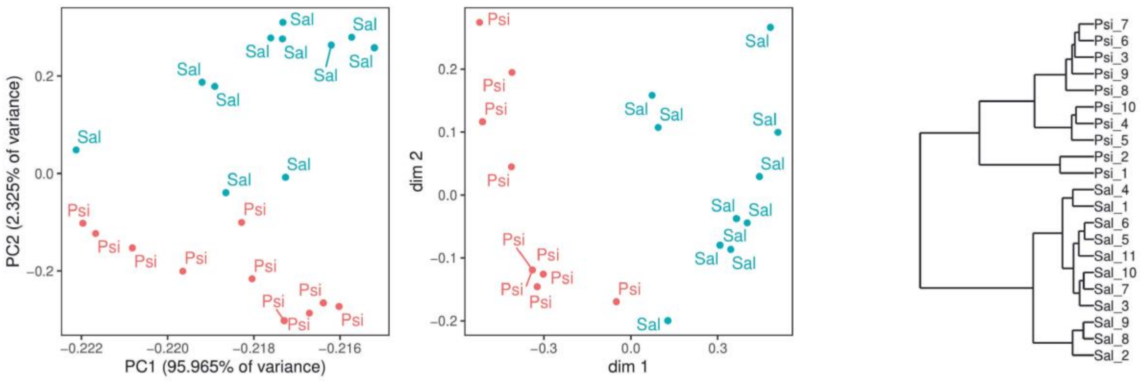

Figure S15 Three types of clustering analyses, including principal component analysis (PCA) (left panel), multidimensional scaling (MDS) (middle panel), and hierarchical clustering with the ward.D2 (right panel), performed based on DESeq2 normalized count table of cingulate cortex samples and DEGs with FDR<0.05.

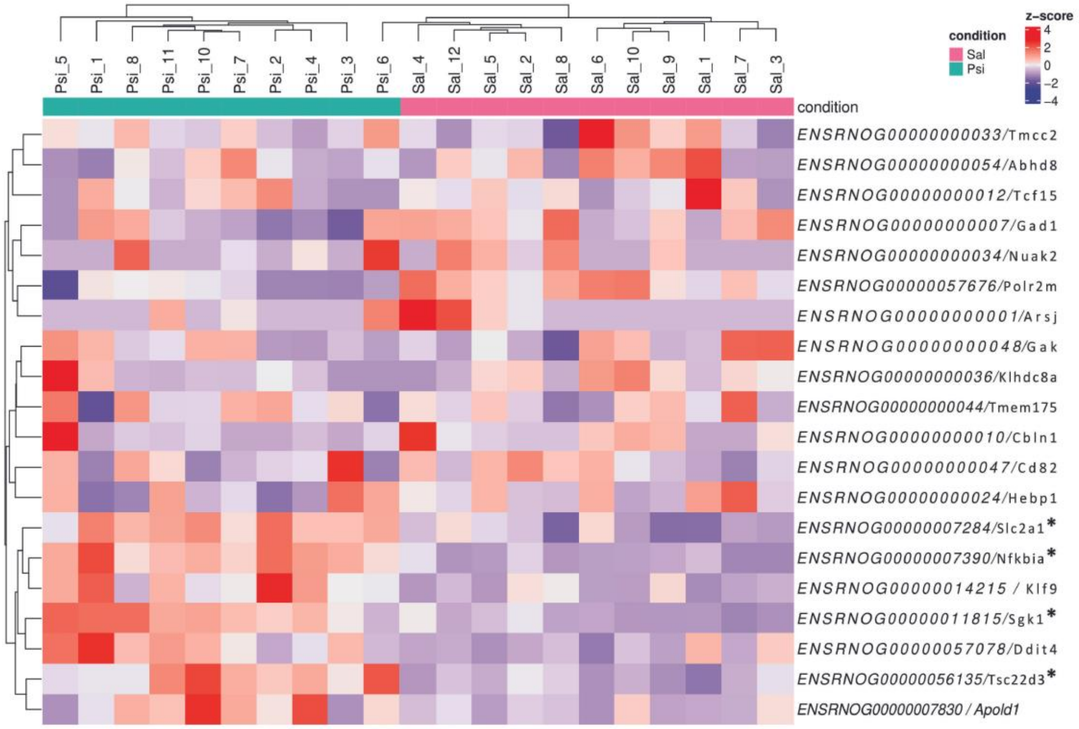

Figure S16 Heatmap of top 20 genes with lowest FDR extracted from DESeq2 result file generated based on cingulate cortex samples' data. Genes with asterisk are identified DEGs with FDR < 0.05 from the DEseq2 result file.

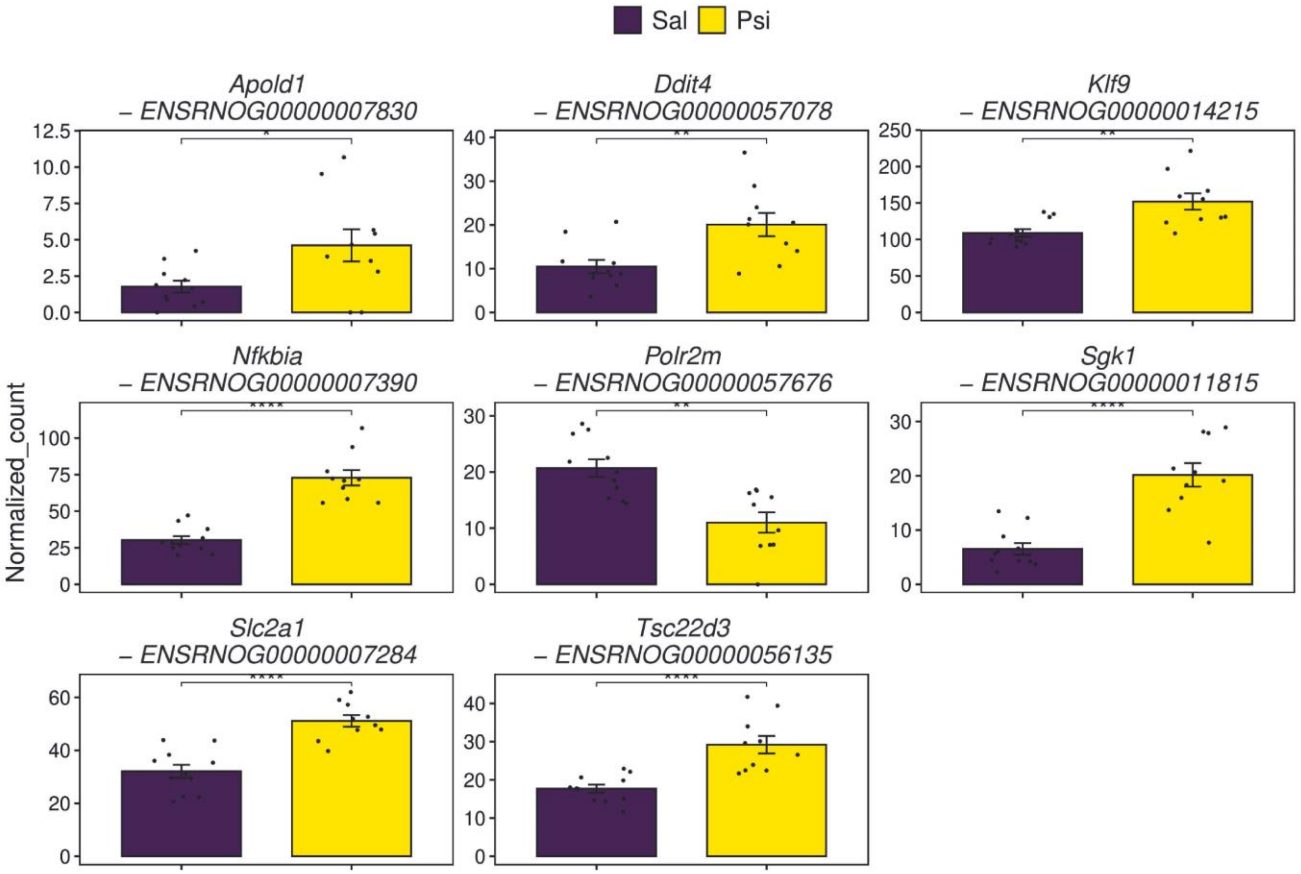

Figure S17 Bar-plot of DEGs with FDR<0.05 generated from DESeq2 normalized count table cingulate cortex samples' data. Wilcoxon test has been applied to show fold change significance between two groups of samples for each DEG: the higher number of stars above pair bars of each DEG, the greater fold change.

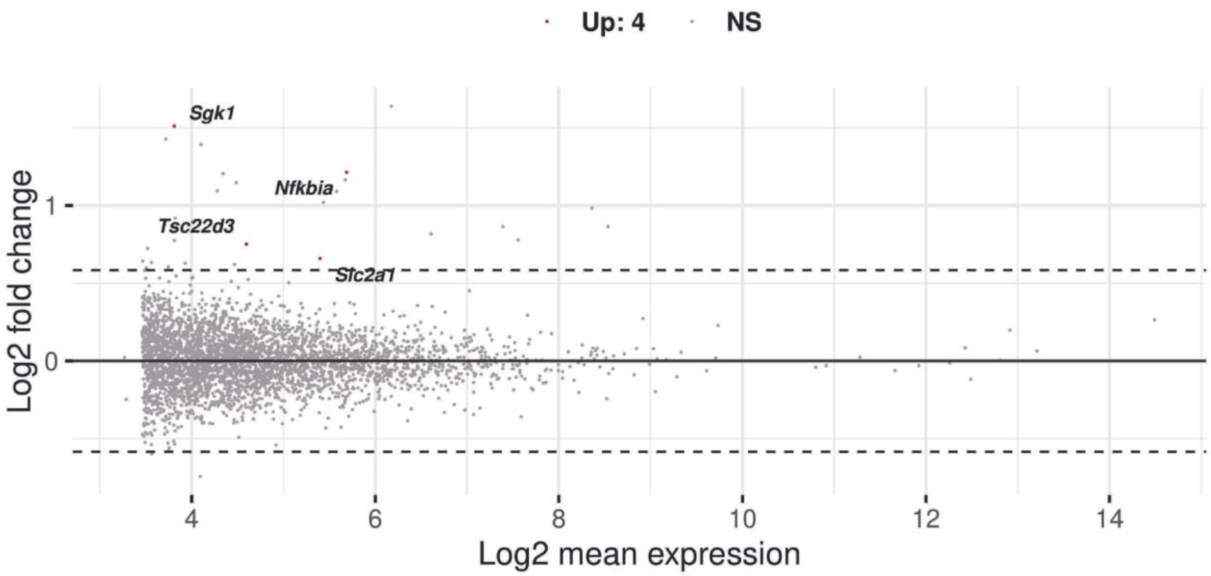

Figure S18 MA-plot based on DEseq2 result file of cingulate cortex samples' data. Three types of thresholds set for generating this plot include FDR < 0.05, FC > 1.5, and BM>10.

Plots generated based on amygdala samples’ data

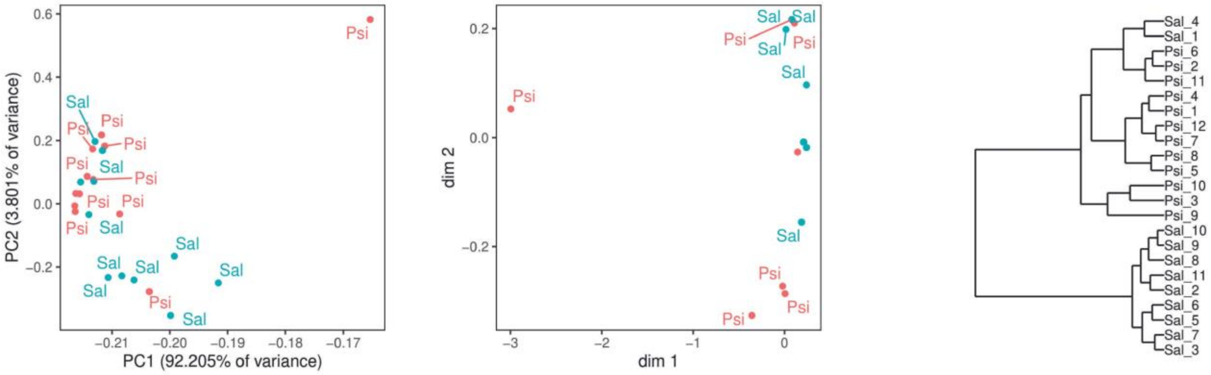

Figure S19 Three types of clustering analyses, including principal component analysis (PCA) (left panel), multidimensional scaling (MDS) (middle panel), and hierarchical clustering with the ward.D2 (right panel), were performed based on DESeq2 normalized count table of amygdala and DEGs with FDR<0.05.

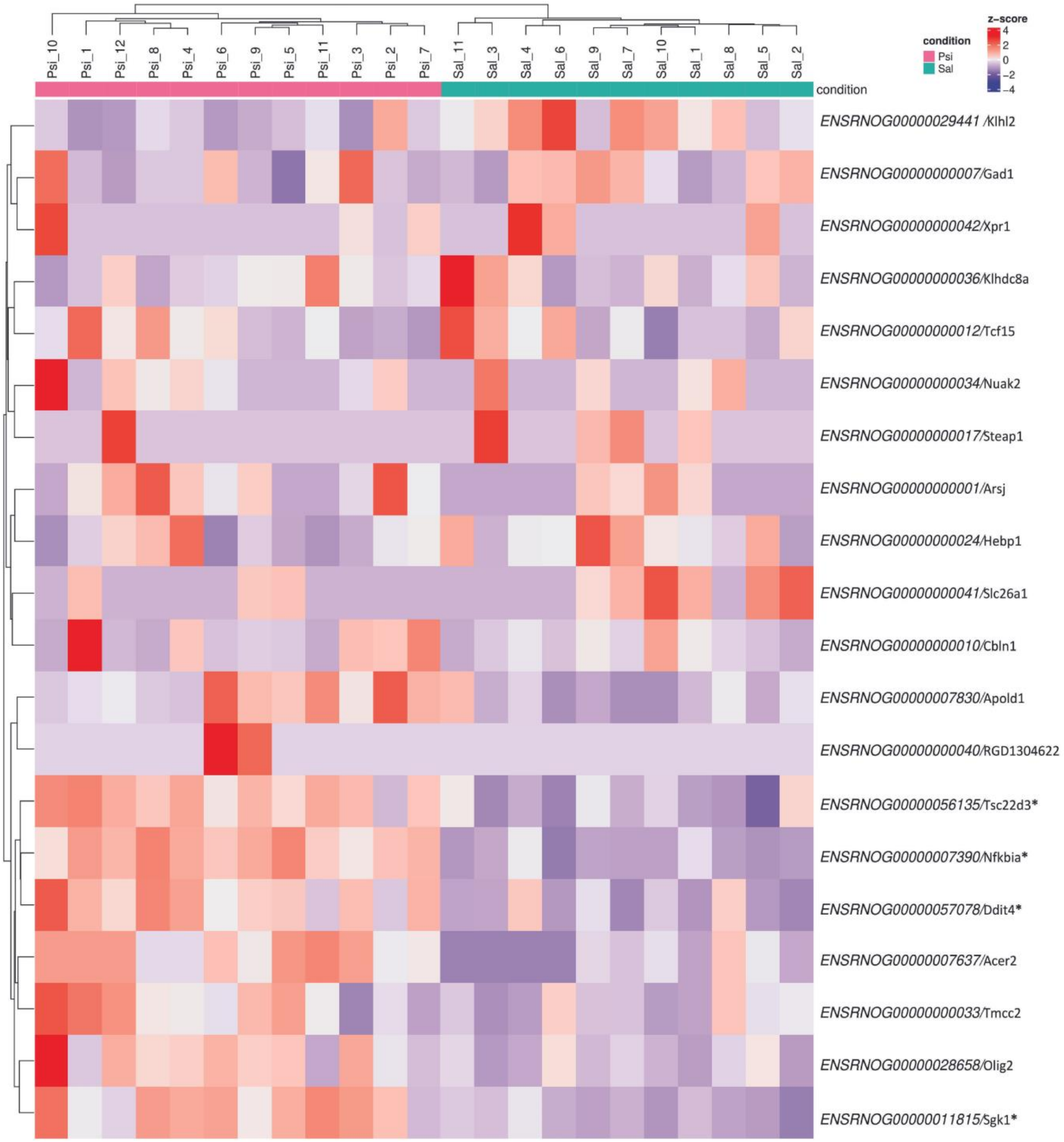

Figure S20 Heatmap of top 20 genes with lowest FDR extracted from DESeq2 result file generated based on amygdala samples' data. Genes with asterisk are identified DEGs with FDR < 0.05 from the DEseq2 result file.

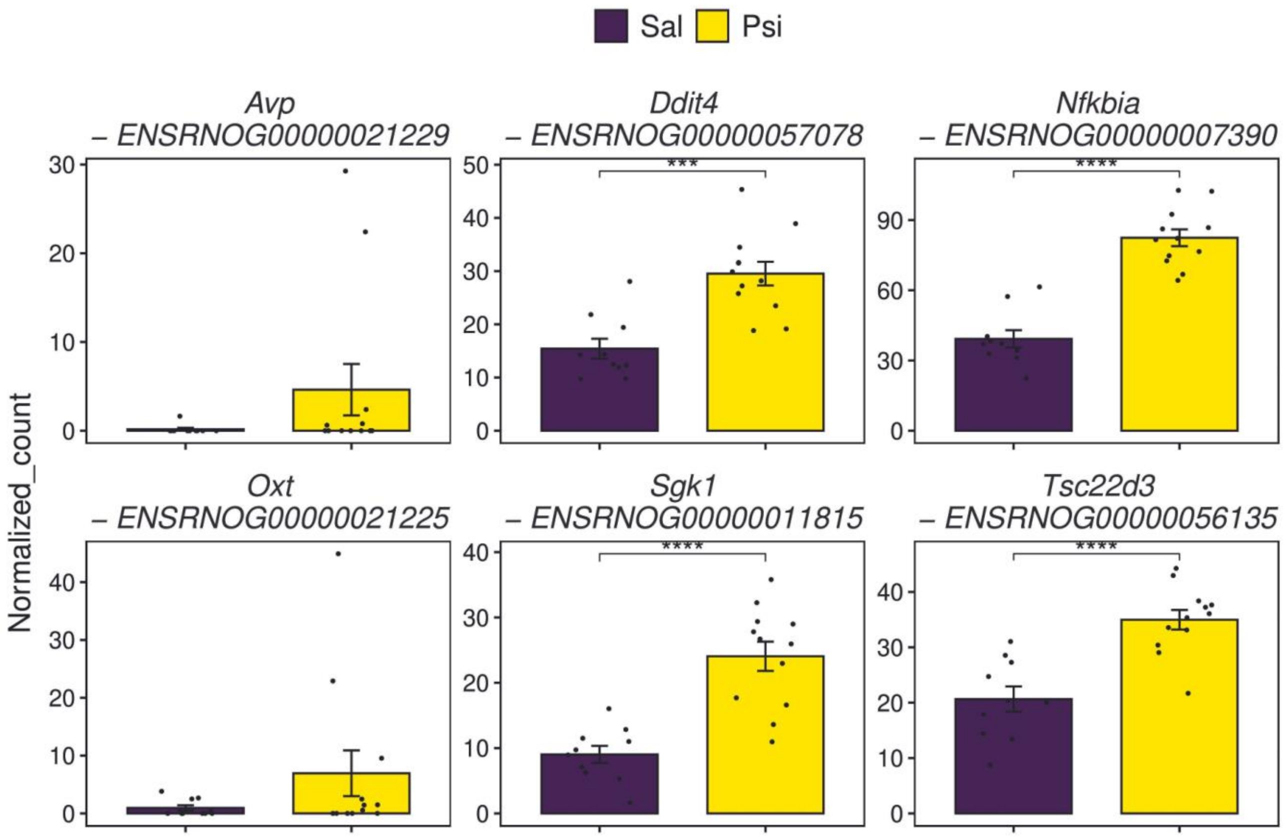

Figure S21 Bar-plot DEGs with FDR<0.05 generated from DESeq2 normalized count table of amygdala samples' data. Wilcoxon test has been applied to show fold change significance between two groups of samples for each DEG: the higher number of stars above pair bars of each DEG, the greater fold change.

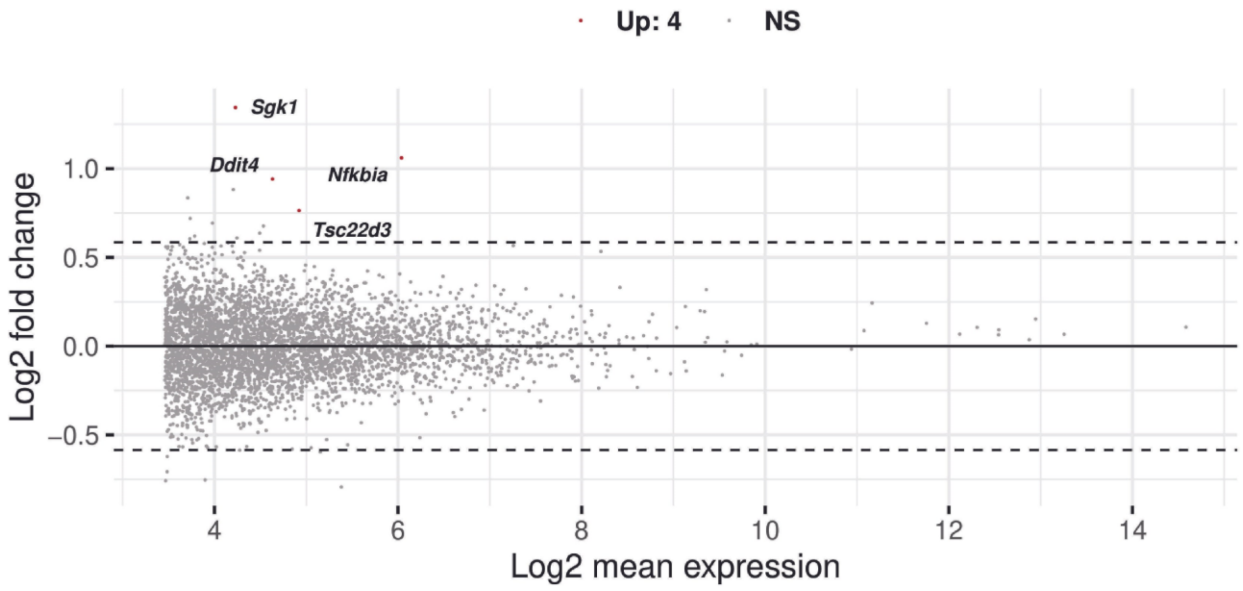

Figure S22 MA-plot based on DEseq2 result file of amygdala samples' data. Three types of thresholds set for generating this plot include FDR < 0.05, FC > 1.5, and BM>10.

Tables

Region	Hemisphere	Ensembl_ID	Gene_Symbol	log2FC	padj	Common
Amygdala	Right	ENSRNOG00000007390	*Nfkbia*	1.0439868	5.076429e-03	Yes
Amygdala	Left	ENSRNOG00000021745	*Bhlhe22*	-1.1617725	2.920164e-02	No
Amygdala	Left	ENSRNOG00000007390	*Nfkbia*	1.0720048	4.802436e-02	Yes
Cingulate cortex	Left	ENSRNOG00000007390	*Nfkbia*	1.2997478	8.754748e-05	No
Hippocampus	Left	ENSRNOG00000011815	*Sgk1*	1.5302704	3.747067e-30	Yes
Hippocampus	Right	ENSRNOG00000011815	*Sgk1*	1.6512456	3.317102e-24	Yes
Hippocampus	Right	ENSRNOG00000007390	*Nfkbia*	1.0402139	3.295122e-07	Yes
Hippocampus	Left	ENSRNOG00000007390	*Nfkbia*	1.0703514	4.419926e-06	Yes
Hippocampus	Left	ENSRNOG00000057078	*Ddit4*	0.8872608	9.855615e-05	Yes

Region	Hemisphere	Ensembl_ID	Gene_Symbol	log2FC	padj	Common
Hippocampus	Right	ENSRNOG00000019009	*Arrdc2*	2.2180125	1.537907e-03	No
Hippocampus	Right	ENSRNOG00000057078	*Ddit4*	0.9557835	4.704143e-03	Yes
Hippocampus	Left	ENSRNOG00000058186	*Errfi1*	0.9353817	9.568526e-03	No
mPFC	Left	ENSRNOG00000007390	*Nfkbia*	1.3771367	1.225477e-05	Yes
mPFC	Right	ENSRNOG00000007390	*Nfkbia*	1.0918011	1.479511e-02	Yes
Striatum	Right	ENSRNOG00000007390	*Nfkbia*	1.2210493	1.140829e-09	No
Striatum	Right	ENSRNOG00000011815	*Sgk1*	1.6924600	5.587213e-09	Yes
Striatum	Left	ENSRNOG00000011815	*Sgk1*	1.7797627	9.742248e-06	Yes

Table S 1. Hemisphere-specific analysis of psilocybin-induced differentially expressed genes. *Differential expression (psilocybin vs. saline) was performed separately for left and right hemispheres. As expected with reduced sample sizes, hemisphere-specific analysis revealed fewer DEGs (1–4 per region) compared to pooled analysis (4–17 per region). Importantly, most detected genes were shared between hemispheres. The small number of hemisphere-unique genes (1–2 per region) suggests that while the core transcriptional response to psilocybin (Sgk1, Nfkbia, Ddit4) is largely conserved across hemispheres, there may be subtle region-specific lateralization in a minority of genes. These findings indicate that hemisphere is not a major confounding factor in our main analysis, and that the pooled results robustly represent the primary drug response.*

Table S 2. *Thermal cycling protocol for qPCR.*

Step	Temperature	Time	Cycles
**Activation**	95°C	60 s	1
**Cycling**			
Denaturation	95°C	5 s	
Annealing	60°C	30 s	35
Extension	72°C	10 s	
**Melt curve**			
Melt	60-95°C*		1

*Continuous temp. increase (1°C/s)

Table S 3 . *Sequencing quality.*

Result table		
**Density (K/ mm2)**	**Cluster PF (%)**	**Cluster Count PF (M)**
149	92	129
**%>= Q30**	**Aligned PhiX (%)**	**PF Read / Sample (M)**
89.7	15	3.1

Number of studies	Gene name
**9**	**Ddit4**, **Errfi1**
**7**	**Klf9**
**6**	**Bcl6**, Fkbp5, **Nfkbia**, **Pdk4**, Mt2/Mt2A
**5**	Adcy9, *Cxxc5,* Dusp1, Eva1a, *Litaf,* Nedd9, Rhob, **Sgk1**, Sult1a1, Tiparp, **Gpd1***, Ctgf*, **Plekhf1***
**4**	Aldoc, Arhgef3, Arl4d, Bcl6b, **Cables1**, *Calm2, Ccnd1,* Cdkn1a, Cdo1, Chst1, *Cyp7b1,* Ehd3, Fzd1, *Gab1, Gap43,* Gjb6, Hepacam, Id1, *Irf1, Jun,* Klf15, Lhfp, Lyve1, Mertk, Mgst1, Mical2, Myh2, Ndrg2, Npy1r, *Nr3c1,* Nudt9, *Osbpl3,* Pim3, *Plscr1,* Prr5, Rasl11b, Rdx, Rhou, *Sall2, Scamp2,* Sdc4, Sesn1, **Slc**25a33, *Sox2, Sox4,* ***Sox9****,* Spsb1, Svil, *Tgfbr1,* Thra, *Tle4,* ***Tmem****109,* Tob2, **Tsc22d3**, *Vps37b,* Wipf3, Wnt16, *Wnt7a,* Il6r/Il6ra, Dgkz*, Mtmr2*, ***Zfp36l1*****,* Azin1*, *Cklf*,* Ppp5c*, *Sema6d*,* Tle3*

***Table S 4.* Consistently glucocorticoid-responsive genes across multiple studies.**
Genes showing the same direction of expression change in at least four independent reports are presented. Up-regulated genes are shown in red; down-regulated genes are shown in blue italics. Asterisks (*) denote genes that exhibited opposite regulation in one treatment group. Genes identified as differentially expressed in the present study are highlighted in bold. This table summarizes data extracted from Figure 1 of reference [1].

Gene	Psilocybin (This Study)	Ketamine	ECT	SSRIs / Imipramine
Sgk1	Up (5/5 regions)	Up[2]	Up[3]	Up[3,4]
Nfkbia	Up (5/5 regions)	NR	Up[3]	Up[3,4]
Ddit4	Up (4/5 regions)	NR	NR	NR
Slc2a1	Up (2/5 regions)	Up[5]	NR	NR
Cartpt	Up (1/5 regions)	Up[6]	NR	NR
Apold1	Up (2/5 regions)	NR	NR	NR
Tsc22d3	Up (2/5 regions)	NR	NR	NR
Sox9	Down (3/5 regions)	NR	NR	NR

***Table S 5**. Comparison of psilocybin-induced transcriptional changes with other antidepressant treatments.* Abbreviations: ECT, electroconvulsive therapy; SSRIs, selective serotonin reuptake inhibitors; NR, not reported in referenced studies.

References

1. Juszczak GR, Stankiewicz AM (2018). Glucocorticoids, genes and brain function. Prog Neuropsychopharmacol Biological Psychiatry 82, 136-168.

2. Ficek J, Zygmunt M, Piechota M, Hoinkis D, Rodriguez Parkitna J, Przewlocki R, Korostynski M. Molecular profile of dissociative drug ketamine in relation to its rapid antidepressant action. *BMC Genomics*. 2016;17:362.

3. Conti B, Maier R, Barr AM, Morale MC, Lu X, Sanna PP, Bilbe G, Hoyer D, Bartfai T. Region-specific transcriptional changes following the three antidepressant treatments electro convulsive therapy, sleep deprivation and fluoxetine. *Molecular Psychiatry*. 2007;12(2):167-89.

4. Hussain BF, Nanavaty IN, Marathe SV, Rajendran R, Vaidya VA. Hippocampal transcriptional and neurogenic changes evoked by combination yohimbine and imipramine treatment. *Progress in Neuropsychopharmacology and Biological Psychiatry*. 2015;61:1-9.

5. Wegman-Points L, Pope B, Zobel-Mask A, Winter L, Wauson E, Duric V, Yuan LL. Corticosterone as a Potential Confounding Factor in Delineating Mechanisms Underlying Ketamine's Rapid Antidepressant Actions. *Frontiers in Pharmacology*. 2020;11:590221.

6. Funayama Y, Li H, Ishimori E, Kawatake-Kuno A, Inaba H, Yamagata H, Seki T, Nakagawa S, Watanabe Y, Murai T, Oishi N, Uchida S. Antidepressant Response and Stress Resilience Are Promoted by CART Peptides in GABAergic Neurons of the Anterior Cingulate Cortex. *Biological Psychiatry: Global Open Science*. 2022;3(1):87-98.
