# Supplementary material for: Psilocybin elicits a conserved glucocorticoid-responsive gene signature across five 5-HT2A receptor-rich brain regions in rat
Source: Acta Neuropsychiatr. 2026 Apr 10;38:e37. doi: 10.1017/neu.2026.10075 (PMC13202413; doi:10.1017/neu.2026.10075)
Supplement: Veysi et al. supplementary material 2 — Veysi et al. supplementary material [file S0924270826100751sup002.pdf]

# Supplement II

## Quality control and Pre-processing of Sequencing data

*QC1 (Striatum, amygdala, CIN, and mPFC). .......... Page 2 - 5*

*QC2 (Hippocampus)..................................... Page  7 - 8*

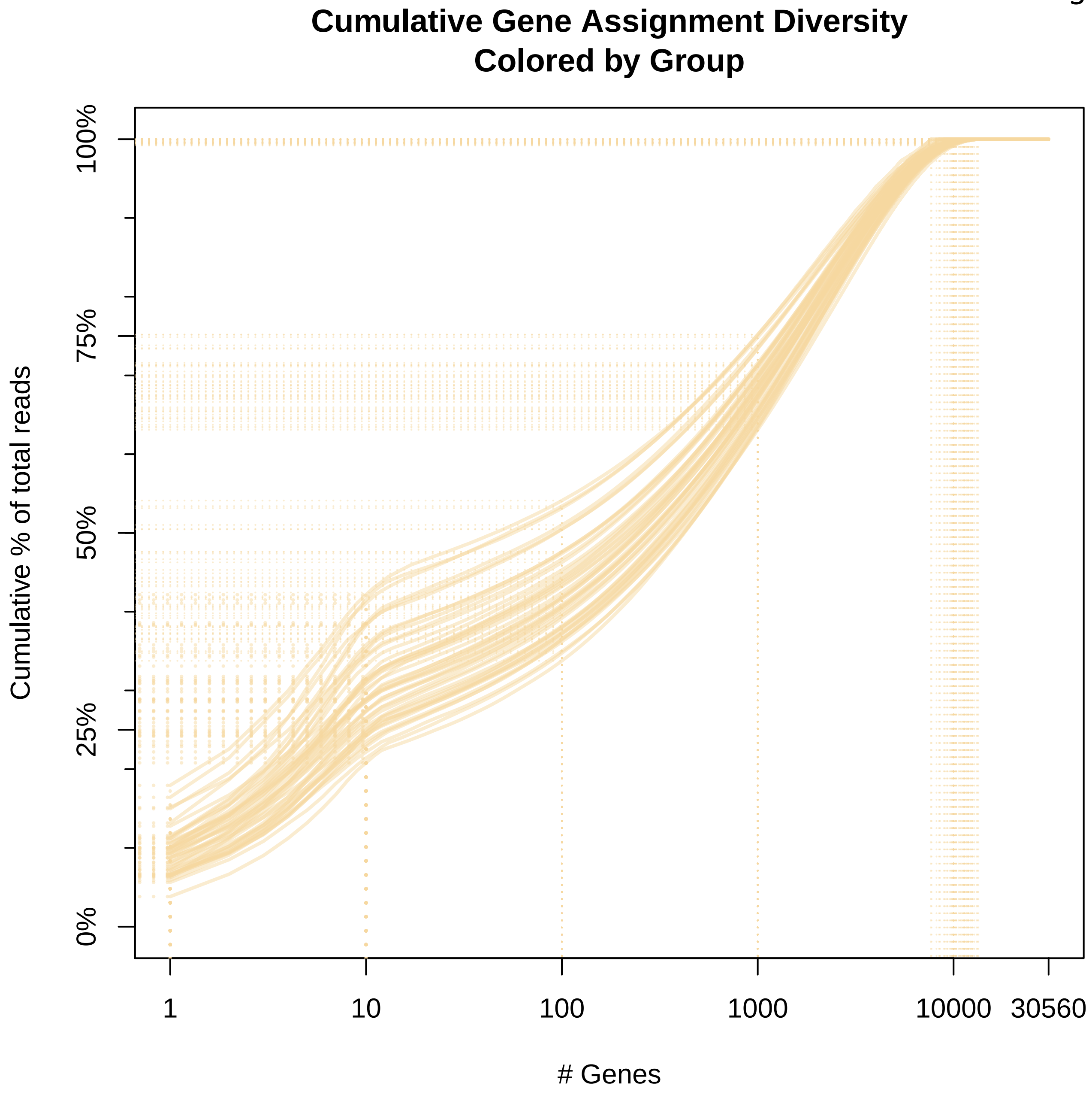
Cumulative Gene Assignment Diversity
Colored by Group
Cumulative % of total reads
100%
75%
50%
25%
0%
1
10
100
1000
10000
30560
# Genes

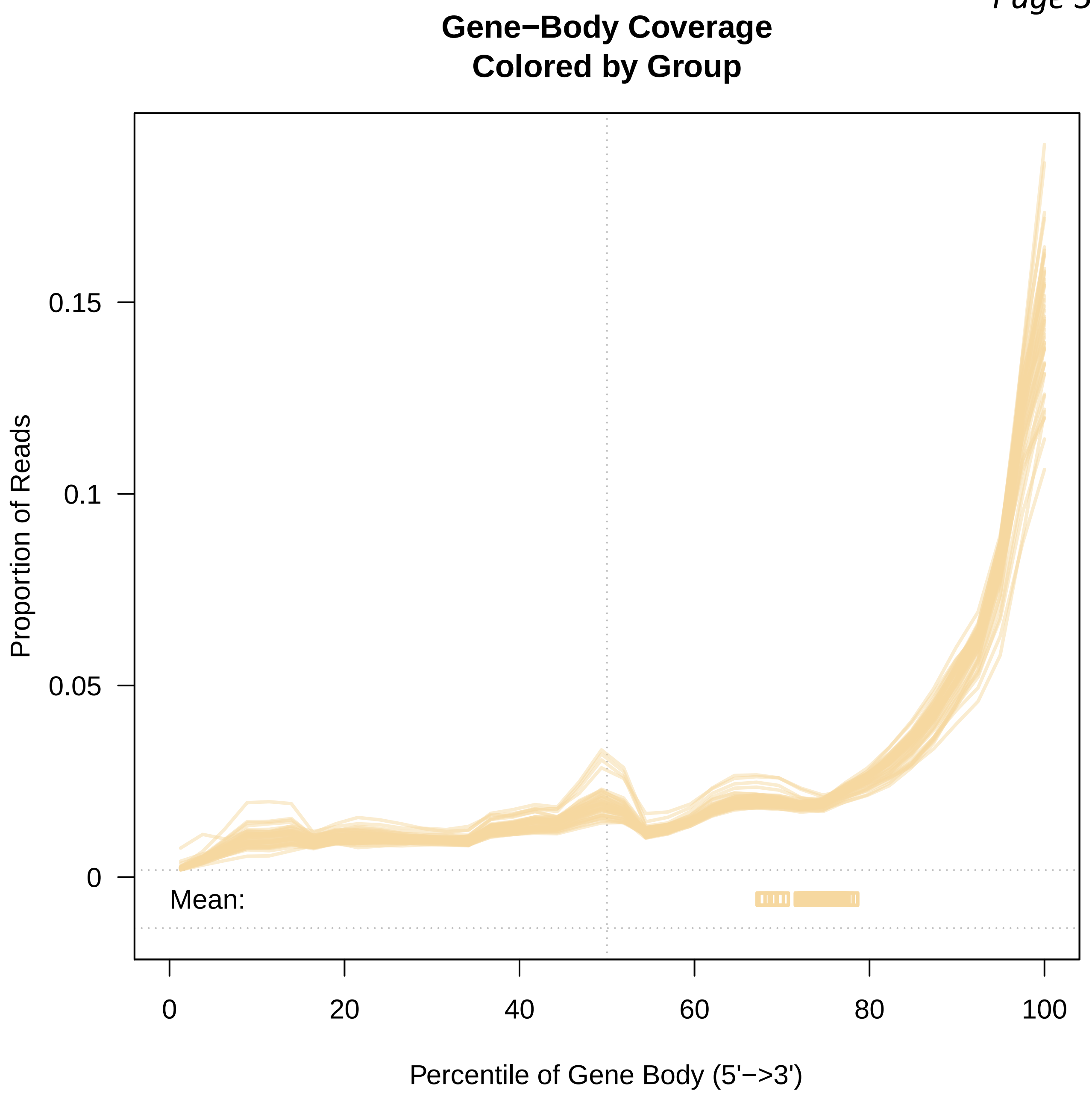
Gene−Body Coverage
Colored by Group
Proportion of Reads
0.15
0.1
0.05
0
Mean:
0
20
40
60
80
100
Percentile of Gene Body (5'−>3')

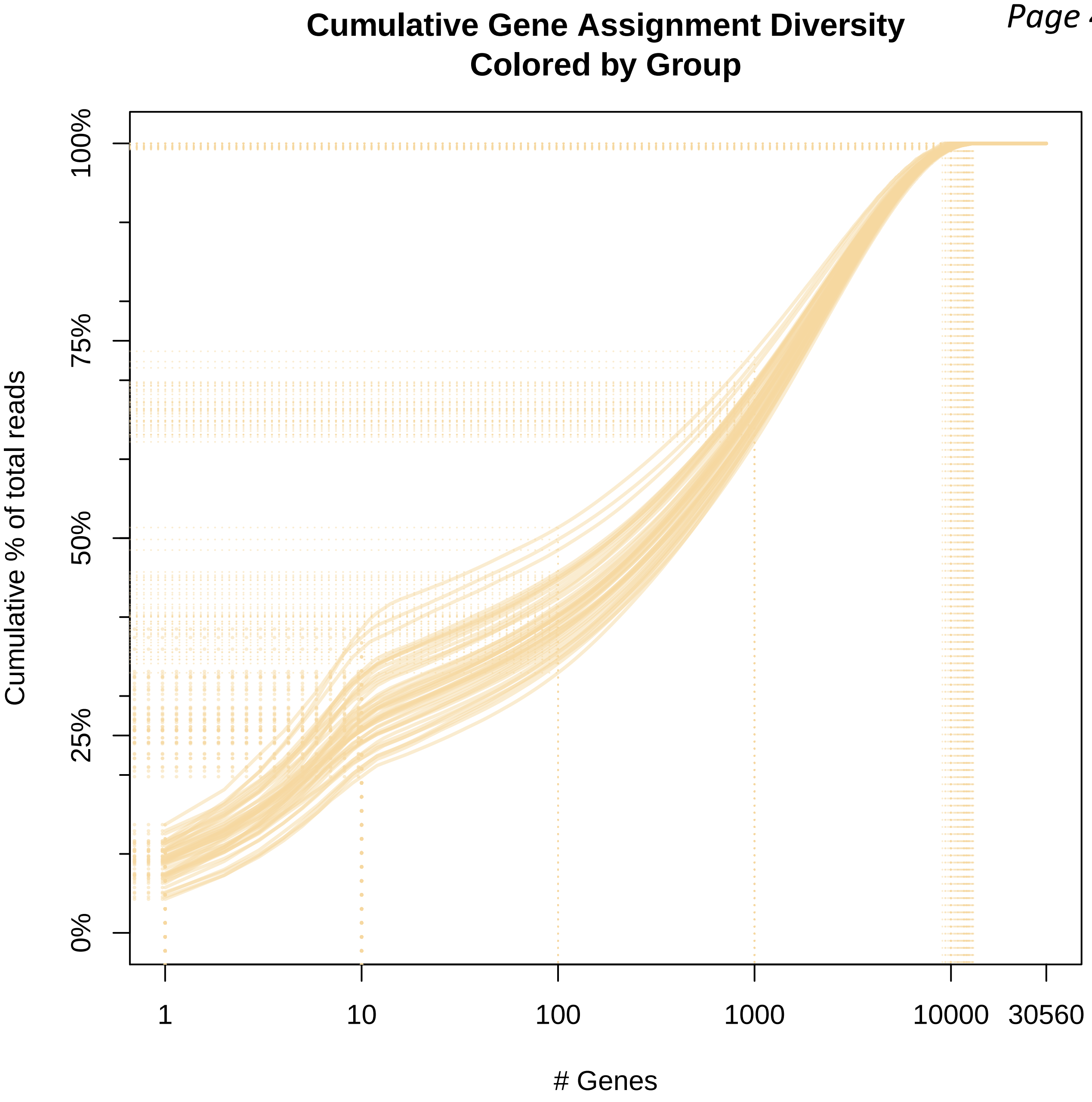
Cumulative Gene Assignment Diversity
Colored by Group
Cumulative % of total reads
0%
25%
50%
75%
100%
# Genes
1
10
100
1000
10000
30560

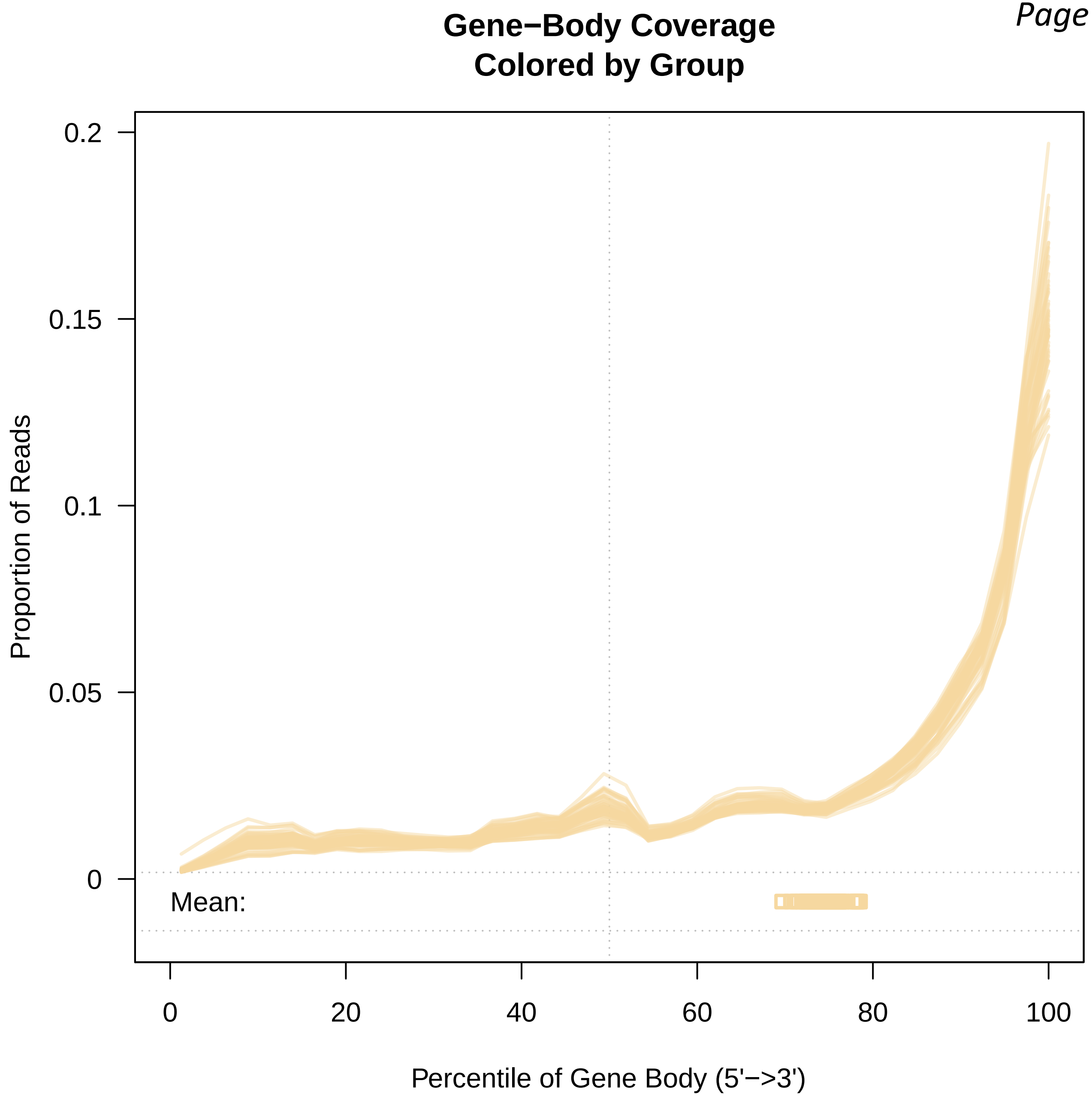
Gene−Body Coverage
Colored by Group
0.2
0.15
0.1
0.05
0
Proportion of Reads
Mean:
0
20
40
60
80
100
Percentile of Gene Body (5'−>3')

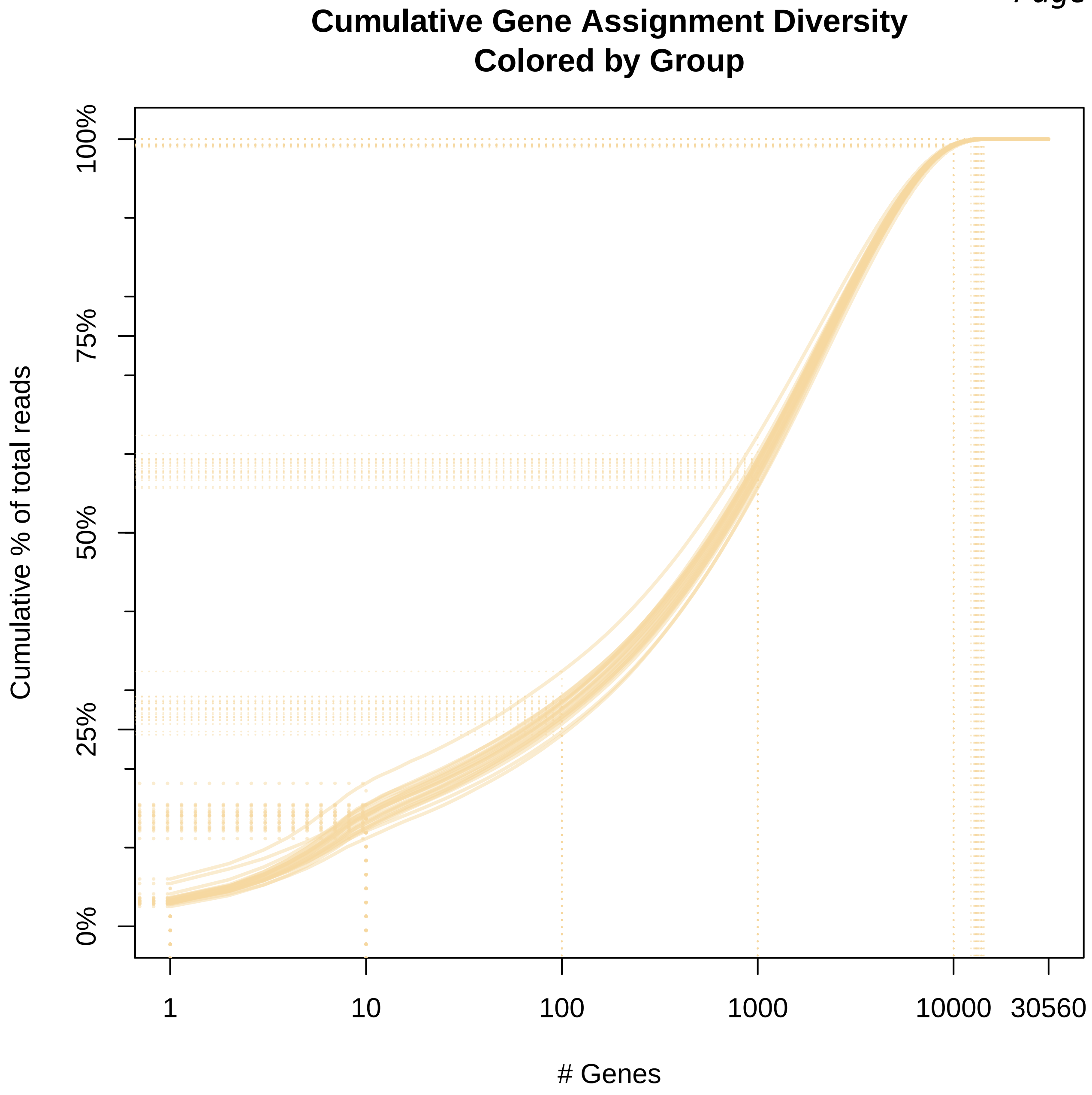
Cumulative Gene Assignment Diversity
Colored by Group
Cumulative % of total reads
0%
25%
50%
75%
100%
# Genes
1
10
100
1000
10000
30560

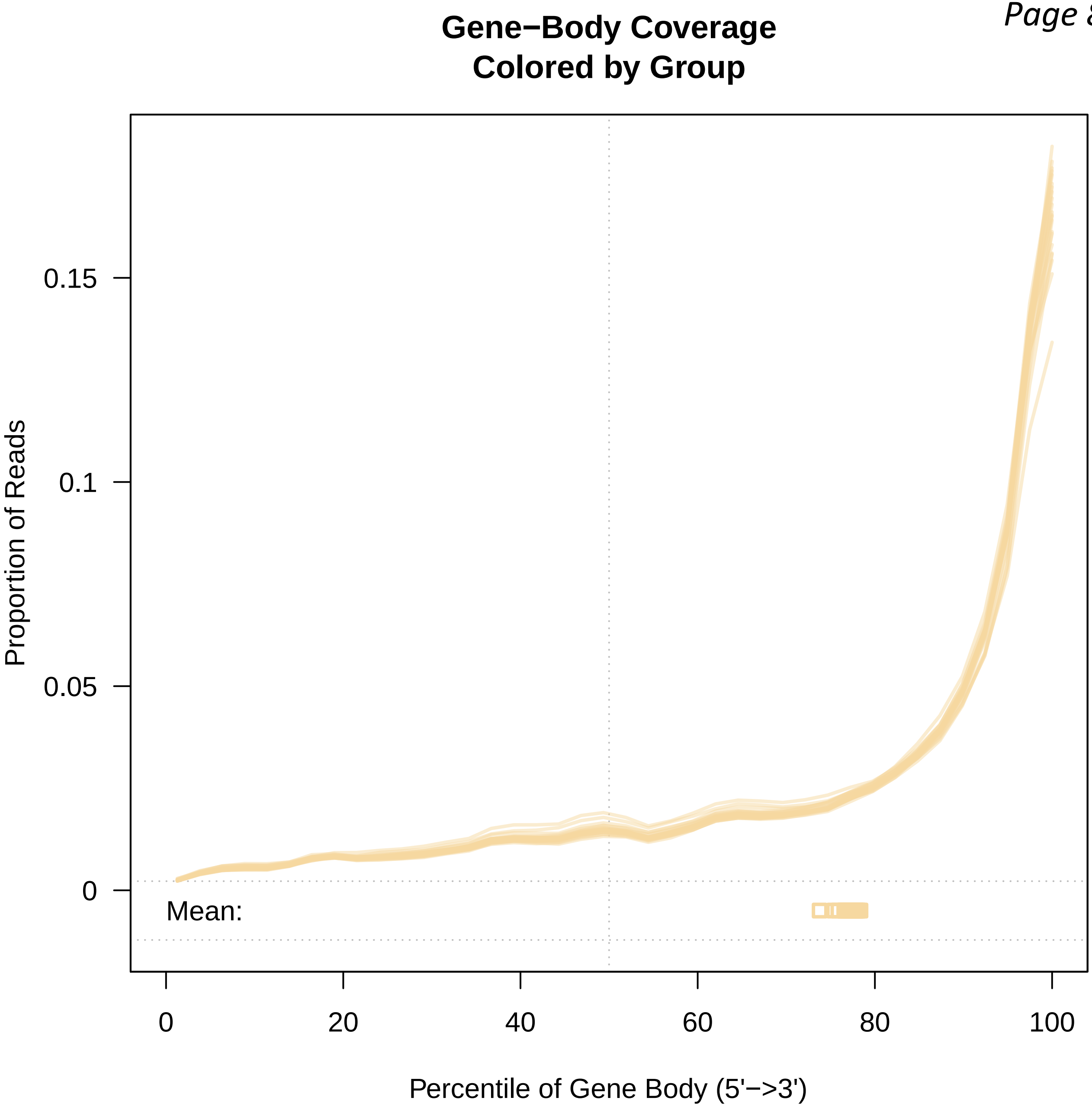
Gene−Body Coverage
Colored by Group
Proportion of Reads
0.15
0.1
0.05
0
Mean:
0
20
40
60
80
100
Percentile of Gene Body (5'−>3')
