# Supplementary material for: Psilocybin elicits a conserved glucocorticoid-responsive gene signature across five 5-HT2A receptor-rich brain regions in rat
Source: Acta Neuropsychiatr. 2026 Apr 10;38:e37. doi: 10.1017/neu.2026.10075 (PMC13202413; doi:10.1017/neu.2026.10075)
Supplement: Veysi et al. supplementary material 3 — Veysi et al. supplementary material [file S0924270826100751sup003.pdf]

Supplement III

Equipment/Kits/Reagents/Document/Software

Striatum, amygdala, CIN, and mPFC. .... *page 2-4*

Hippocampus. .... *page 5-9*

[*Gene Expression Analysis in rat brain with 3'mRNA-seq*]

List of materials

Library Preparation	
	**Name**
**Library Preparation Kit**	Lexogen QuantSeq 3' mRNA-Seq Library Prep kit from Lexogen (FWD)
**Index**	Lexogen UMI Second Strand Synthesis Module for QuantSeq FWD (Illumina, Read 1)
	Lexogen UDI 12 nt Unique Dual Indexing Add-on Kits: A2 (UDI 12A 0097-0192)
**Purification Module**	*Purification Module with Magnetic Beads*
**Cycle Det. Module**	*PCR Add-on Kit for Illumina, 96 rxn*
**UHR**	*UHR RNA QuantiGene Reagent Invitrogen*
**ERCC**	*SIRV-Set 3 (Iso Mix E0 / ERCC)*
**Ethanol**	*Absolute Ethanol*
**Water**	*Invitrogen Ultrapure distilled water (DNase/RNase free)*
**Fragment Analysis**	
	**Name**
**Frag. Analysis-kit**	***High Sensitivity NGS Fragment Analysis Kit***
	5X 930 dsDNA Inlet Buffer
	NGS Separation Gel
	Intercalating Dye
	0.25X TE Rinse Buffer
	HS-NGS Diluent Marker
**NGS**	
	**Name**
**SBS cartridge**	Kit NovaSeq 6000 SP/S1/S2 v1.5 SBS Cartridge
**Flow cell**	NovaSeq 6000 S1 flow cell
**Buffer cartgridge**	NovaSeq 6000 SP cluster cartridge v1.5
**PicoGreen**	Invitrogen Quant-iT PicoGreen dsDNA Assay Kit
**Tris-HCl**	Invitrogen UltraPure 1M Tris-HCl pH 8.5
**Tris-HCl**	Invitrogen UltraPure 1M Tris-HCl pH 8.0
**NaOH**	10N NaOH

Kits protocols

3' mRNA-Seq Library Prep Kit FWD with Unique Dual Indices User Guide. Reference: 113UG227V0110.
113UG227V0110_QuantSeq_FWD_UDI_Kits_2021-08-16.pdf (lexogen.com)

Lexogen UDI 12 nt Unique Dual Indexing Add-on Kits User Guide. Reference: 107UG223V0200
107UG223V0200_Lexogen-12-nt-Unique-Dual-Indexing-Add-on-Kit_2021-02-23.pdf

Unique Molecular Identifiers for QuantSeq User Guide. Reference: 081UG366V0100
081UG366V0100_UMI-Module-for-QuantSeq_2021-08-16.pdf (lexogen.com)

Reamplification Add-on Kit for Illumina Instruction Manual. Reference: 080IM169V0102
080IM169V0102_Reamplification-Add-on-Kit_2020-08-25.pdf (lexogen.com)

Kit/Equipment /Material	Manufacturer/Method/ Catalogue number/Detail
Spectrophotometer	Lunatic, Unchained Labs
Capillary gel electrophoresis	DNF-471-Standard Sensitivity RNA 15 nt, Fragment Analyzer, Agilent
Universal Human Reference (UHR) sample	Cat. No. QS0639, ThermoFisher Scientific
ERCC spike	Cat. No. 445670, ThermoFisher Scientific
UDI 12A 0097-0192	Cat. No. 119.384, Lexogen
PCR Add-on Kit	Cat. No. 020.96, Lexogen
SYBR Green I	Cat. No. S7563, ThermoFisher Scientific
capillary gel electrophoresis	Fragment Analyzer, Agilent
High Sensitivity NGS Fragment Analysis Kit	Cat. No. DNF-474, Agilent
QuantStudio 12K Flex platform	ThermoFisher Scientific
TATAA's NGS Library Quantification Kit	Cat. No. TA20-NGSQ, TATAA Biocenter
Re-amplification Add-on Kit	Cat. No. 080.96, Lexogen
Quant-it PicoGreen dsDNA Assay Kit	Cat. No. P7589, ThermoFisher Scientific
Nanodrop ND3300	ThermoFisher Scientific
PhiX spike-in	Cat. No. 15017666, Illumina
NovaSeq 6000 Denature and Dilute Libraries Guide	Illumina, Document # 1000000106351 v03
S1 Reagent Kit v1.5, 100 cycles	Cat. No. 20028312, Illumina
NovaSeq 6000 Sequencing System Guide	Illumina, Document # 1000000019358 v14

PROJECT SUMMARY

Hippocampus samples

Technology	3prime-RNASeq
Sample Type	RNA / *Rattus norvegicus*
Reference Genome	mRatBN7.2
Sample Receival / Storage	Tubes / Tissue / -80 °C
Sample Number	24
ISO17025 extraction	No
Library Preparation	QuantSeq 3' mRNA-Seq FWD
Selection	PolyA
UDI	UDI12A
Sequencer	Illumina NextSeq 500
Sequencing Configuration	SE 1 x 75, Mid Output Flowcell
PhiX spike-in	8 %
ERCC spike-in	No
UMI	NNNNNNTATA
Deliverables	Extraction QC, Library QC, Sequencing QC, FASTQ files, Data analysis result files

TATAA Biocenter AB
Sofierogatan 3A, 412 51 Göteborg
Tel: +46 31 761 57 00 Fax: +46 31 15 28 90
www.tataa.com

REAGENTS

Detailed information		
**Name**	**Catalogue nr**	**Vendor**
Fatty Tissue RNA Purification kit	36200	Norgen Biotek
Bead Tubes	26533	Norgen Biotek
RNA Standard Sensitivity Fragment Analyzer Kit	DNF-471	Agilent Technologies Inc
Universal Human Reference RNA	QS0639	ThermoFisher Scientific
QuantSeq 3'mRNA-Seq Library Prep Kit FWD	015.24	Lexogen
UDI 12nt Set A1	198.94	Lexogen
UMI Second Strand Synthesis Module for QuantSeq FWD	081.96	Lexogen
PCR Add-on Kit	020.24	Lexogen
SYBR Green I	S7563	ThermoFisher Scientific
High Sensitivity NGS Fragment Analysis Kit	DNF-474	Agilent
TATAA NGS Library Quantification Kit	TA20-NGSQ	TATAA Biocenter
TATAA SYBR GrandMaster Mix	TA01-625	TATAA Biocenter
Quant-it PicoGreen dsDNA Assay Kit	P7589	ThermoFisher Scientific
PhiX	FC-110-3001	Illumina
NextSeq 500/550 Mid Output Kit v2.5	20024904	Illumina

EQUIPMENT

Detailed information

Type	Name	Vendor
Sample disruption	TissueLyser II	Qiagen
qPCR I	CFX Opus 96	Bio-Rad
qPCR II	QuantStudio 7	ThermoFisher Scientific
Spectrophotometer	Lunatic	Unchained Labs
Capillary gel electrophoresis	Fragment Analyzer 12	Agilent
Fluorescence spectrophotometer	Nanodrop ND3300	ThermoFisher Scientific
Sequencing Platform	NextSeq500	Illumina

DOCUMENTS

Detailed information

Name	Number	version
3' mRNA-Seq Library Prep Kit FWD with Unique Dual Indices Guide	113UG227V0120	v.01
NextSeq System Denature and Dilute Libraries Guide	15048776	v.18
NextSeq 500 Sequencing System Guide	15069765	v.07
Lexogen - QuantSeq data analysis guide	-	https://www.lexogen.com/wp-content/uploads/2021/05/015UG108V0311_QuantSeq-Data-Analysis-Pipeline_2021-05-04.pdf

SOFTWARE

Detailed information

Type	Name	Vendor
LIMS	The Benchling R&D Cloud	Benchling
Electronic lab book	Biovia Notebook	Dassault Systèmes
Cloud storage	SharePoint - Office 365	Microsoft

DATA ANALYSIS SOFTWARE

Detailed information

Type	Name	Version
Demultiplexing	bcl2fastq	2.20.0.422
Quality Control	FastQC	0.11.9
Quality Control	MultiQC	1.12
UMI processing and deduplication	UMI-tools	1.1.2
Read trimming	BBMap/BBDuk	38.96
Alignment	Star	2.7.10
Gene counts	HTSeq	2.0.1
Alignment statistics	QoRTs	1.3.6
Statistical computing	R	4.2.3
DGE	DESeq2	1.38.3
Pathway	clusterProfiler	4.6.2
Pathway	ReactomePA	1.42.0
Pathway	biomaRt	2.54.1

TATAA Biocenter AB
Sofierogatan 3A, 412 51 Göteborg
Tel: +46 31 761 57 00 Fax: +46 31 15 28 90
www.tataa.com
