# Supplementary material for: Psilocybin elicits a conserved glucocorticoid-responsive gene signature across five 5-HT2A receptor-rich brain regions in rat
Source: Acta Neuropsychiatr. 2026 Apr 10;38:e37. doi: 10.1017/neu.2026.10075 (PMC13202413; doi:10.1017/neu.2026.10075)
Supplement: Veysi et al. supplementary material 4 — Veysi et al. supplementary material [file S0924270826100751sup004.pdf]

Supplement IV

TATAA BIOCENTER

Project #211001-RC-GU-SE-Elias Eriksson-QuantSeq

TATAA-ID	Region	Rat	Treatment	(…)	Hemisphere	Customer Sample Name	Extraction	Library Plate #	Sample Plate Row	Sample Plate Column	Concentration (ng/ul)	A260/A230	A260/A280	RIN	Total Reads	%reads >= phred 30
1	Mpfc	1	Saline	(…)	L	M_1S(…)L	2	42	B	6	42,7	1,51	2,11	9,4	7057647	94,57
2	Mpfc	1	Psilocybin	(…)	R	M_1P(…)R	2	32	H	4	39	1,56	2,08	9,3	455638	95,57
3	Mpfc	2	Psilocybin	(…)	L	M_2P(…)L	2	31	G	4	39,7	1,46	2,07	9,5	2	100
4	Mpfc	2	Saline	(…)	R	M_2S(…)R	4	88	H	11	32,2	1,4	2,05	8,9	4357347	95,13
5	Mpfc	3	Saline	(…)	L	M_3S(…)L	4	73	A	10	5	1,07	1,91	9,5	5933117	90,65
6	Mpfc	3	Psilocybin	(…)	R	M_3P(…)R	2	35	C	5	39,5	1,48	2,07	9,3	2342973	96,03
7	Mpfc	4	Psilocybin	(…)	L	M_4P(…)L	2	39	G	5	25,3	1,3	2,01	9,2	4525099	96,02
8	Mpfc	4	Saline	(…)	R	M_4S(…)R	4	74	B	10	51,6	1,37	2,02	9,3	13011733	90,01
9	Mpfc	5	Saline	(…)	L	M_5S(…)L	2	48	H	6	67,4	1,76	2,09	9,3	7404632	93,58
10	Mpfc	5	Psilocybin	(…)	R	M_5P(…)R	1	2	B	1	25,1	1,34	2,07	9,8	6347683	95,75
11	Mpfc	6	Saline	(…)	L	M_6S(…)L	2	40	H	5	12	0,93	1,91	9	6365676	92,87
12	Mpfc	6	Psilocybin	(…)	R	M_6P(…)R	1	6	F	1	23,7	1,21	2,17	9	5287317	93,74
13	Mpfc	7	Psilocybin	(…)	L	M_7P(…)L	1	16	H	2	26	1,38	2,27	9,2	14539491	92,25
14	Mpfc	7	Saline	(…)	R	M_7S(…)R	4	84	D	11	5	1,17	2,05	8,6	7255876	94,82
15	Mpfc	8	Saline	(…)	L	M_8S(…)L	2	44	D	6	58,7	1,68	2,12	9,2	7960460	95,75
16	Mpfc	8	Psilocybin	(…)	R	M_8P(…)R	1	5	E	1	18,6	1,22	2,07	8,7	3994126	96,19
17	Mpfc	9	Psilocybin	(…)	L	M_9P(…)L	4	79	G	10	48,3	1,51	2,02	9,1	2315467	95,06
18	Mpfc	9	Saline	(…)	R	M_9S(…)R	1	1	A	1	15,1	1,08	2,24	9,8	391348	92,46
19	Mpfc	10	Saline	(…)	L	M_10S(…)L	3	70	F	9	18,4	1,39	2,09	9,1	8313966	95,24
20	Mpfc	10	Psilocybin	(…)	R	M_10P(…)R	3	66	B	9	25,9	1,4	2,12	9,3	1583270	96,07
21	Mpfc	11	Psilocybin	(…)	L	M_11P(…)L	4	90	B	12	30,6	1,41	2,11	9,3	5643584	94,25
22	Mpfc	11	Saline	(…)	R	M_11S(…)R	4	86	F	11	16,2	1,03	2,02	8,6	1540075	95,49
23	Mpfc	12	Saline	(…)	L	M_12S(…)L	3	54	F	7	21,8	1,37	2,26	8,9	7136092	93,86
24	Mpfc	12	Psilocybin	(…)	R	M_12P(…)R	2	45	E	6	18,6	1,19	2,14	9,4	2087679	95,64
25	Cigulate	1	Saline	(…)	L	C_1S(…)L	3	56	H	7	26,4	1,51	2,19	8,9	5672822	92,52
26	Cigulate	1	Psilocybin	(…)	R	C_1P(…)R	2	38	F	5	23	1,22	2,08	9,1	1966440	94,29
27	Cigulate	2	Psilocybin	(…)	L	C_2P(…)L	4	93	E	12	4,4	0,78	2,21	10	8501181	91,71
28	Cigulate	2	Saline	(…)	R	C_2S(…)R	1	15	G	2	29,7	1,32	2,2	8,9	9241026	95,19
29	Cigulate	3	Saline	(…)	L	C_3S(…)L	3	67	C	9	37,5	1,57	2,11	9,2	6199085	88,31
30	Cigulate	3	Psilocybin	(…)	R	C_3P(…)R	4	82	B	11	24,5	1,25	2,04	8,8	1332316	91,19
31	Cigulate	4	Psilocybin	(…)	L	C_4P(…)L	1	14	F	2	32,1	1,36	2,16	9,1	985141	96,24
32	Cigulate	4	Saline	(…)	R	C_4S(…)R	2	41	A	6	38,9	1,54	2,07	9,1	5464237	94,57
33	Cigulate	5	Saline	(…)	L	C_5S(…)L	1	12	D	2	19,9	1,15	2,2	9,3	9443423	93,14
34	Cigulate	5	Psilocybin	(…)	R	C_5P(…)R	4	92	D	12	92,8	1,65	2,07	9,3	642171	95,6
35	Cigulate	6	Saline	(…)	L	C_6S(…)L	4	77	E	10	6	1,03	1,95	9	1197201	93,83
36	Cigulate	6	Psilocybin	(…)	R	C_6P(…)R	2	26	B	4	51,2	1,63	2,1	9,1	272940	96,07
37	Cigulate	7	Psilocybin	(…)	L	C_7P(…)L	3	55	G	7	17,9	1,29	2,16	9,1	6963121	94,23
38	Cigulate	7	Saline	(…)	R	C_7S(…)R	3	71	G	9	34,2	1,59	2,15	9,3	6241672	96,15
39	Cigulate	8	Saline	(…)	L	C_8S(…)L	1	20	D	3	36,6	1,58	2,19	9,2	1244019	94,73
40	Cigulate	8	Psilocybin	(…)	R	C_8P(…)R	1	4	D	1	19,9	1,13	2,11	9,7	14852306	92,37
41	Cigulate	9	Psilocybin	(…)	L	C_9P(…)L	2	27	C	4	28	1,4	2,05	9	12353268	86,51
42	Cigulate	9	Saline	(…)	R	C_9S(…)R	3	52	D	7	8,1	1,03	2,33	10	6393738	94,43
43	Cigulate	10	Saline	(…)	L	C_10S(…)L	3	49	A	7	3,6	0,67	2	10	2839719	94,78
44	Cigulate	10	Psilocybin	(…)	R	C_10P(…)R	2	29	E	4	7,5	0,65	2,07	9,2	465471	95,4
45	Cigulate	11	Psilocybin	(…)	L	C_11P(…)L	3	57	A	8	8	1,09	2,17	10	4026319	95,04
46	Cigulate	11	Saline	(…)	R	C_11S(…)R	4	96	H	12	42,1	1,36	2,02	8,6	10319053	94,6
47	Cigulate	12	Saline	(…)	L	C_12S(…)L	1	11	C	2	17,1	1,09	2,36	9,1	5859414	94,86
48	Cigulate	12	Psilocybin	(…)	R	C_12P(…)R	2	34	B	5	25,7	1,29	2,09	9,5	5535500	91,42
49	Striatum	1	Saline	(…)	L	S_1S(…)L	4	91	C	12	87	1,64	2,08	9	515105	96,15
50	Striatum	1	Psilocybin	(…)	R	S_1P(…)R	1	23	G	3	141,6	1,97	2,13	9,1	1557159	95,11
51	Striatum	2	Psilocybin	(…)	L	S_2P(…)L	3	63	G	8	126,1	1,93	2,11	9,3	9020613	93,81
52	Striatum	2	Saline	(…)	R	S_2S(…)R	1	21	E	3	72,8	1,8	2,14	9	19111687	92,46
53	Striatum	3	Saline	(…)	L	S_3S(…)L	2	30	F	4	119,3	1,83	2,09	8,9	12175226	90,24
54	Striatum	3	Psilocybin	(…)	R	S_3P(…)R	2	33	A	5	84,3	1,79	2,11	9,2	4780983	95,67
55	Striatum	4	Psilocybin	(…)	L	S_4P(…)L	2	37	E	5	102,6	1,9	2,09	9,4	5554585	92,04
56	Striatum	4	Saline	(…)	R	S_4S(…)R	3	58	B	8	126,3	1,95	2,1	9,4	9385258	95,13
57	Striatum	5	Saline	(…)	L	S_5S(…)L	1	19	C	3	104,3	1,92	2,14	9,2	3421088	93,2
58	Striatum	5	Psilocybin	(…)	R	S_5P(…)R	2	43	C	6	87,3	1,82	2,1	9,7	2561027	95,73
59	Striatum	6	Saline	(…)	L	S_6S(…)L	3	61	E	8	58,8	1,73	2,13	9,2	7231174	94,85
60	Striatum	6	Psilocybin	(…)	R	S_6P(…)R	4	94	F	12	115,7	1,76	2,09	9	831638	96,37
61	Striatum	7	Psilocybin	(…)	L	S_7P(…)L	2	28	D	4	114,4	1,91	2,09	9,4	26373350	93,4
62	Striatum	7	Saline	(…)	R	S_7S(…)R	4	95	G	12	99,9	1,72	2,06	8,9	N/A	N/A
63	Striatum	8	Saline	(…)	L	S_8S(…)L	3	69	E	9	72,1	1,84	2,12	9,3	775577	96,18
64	Striatum	8	Psilocybin	(…)	R	S_8P(…)R	1	13	E	2	72,9	1,71	2,12	9,5	5434477	96,34
65	Striatum	9	Psilocybin	(…)	L	S_9P(…)L	3	59	C	8	80	1,89	2,11	9,4	2938789	96,02
66	Striatum	9	Saline	(…)	R	S_9S(…)R	4	78	F	10	34,2	1,28	2	8,7	6567958	93,32
67	Striatum	10	Saline	(…)	L	S_10S(…)L	3	65	A	9	85,2	1,89	2,12	9,8	1366855	94,47
68	Striatum	10	Psilocybin	(…)	R	S_10P(…)R	4	87	G	11		N/A	N/A	10	2143658	95,45
69	Striatum	11	Psilocybin	(…)	L	S_11P(…)L	4	76	D	10	59,9	1,43	2,04	9,1	1157493	95,81
70	Striatum	11	Saline	(…)	R	S_11S(…)R	2	46	F	6	138,6	1,95	2,1	9,2	7205523	95,23
71	Striatum	12	Saline	(…)	L	S_12S(…)L	3	62	F	8	64,3	1,76	2,16	9,3	4994129	90,25
72	Striatum	12	Psilocybin	(…)	R	S_12P(…)R	3	53	E	7	74	1,82	2,12	9,1	5936479	96,3
73	Amygdala	1	Saline	(…)	L	A_1S(…)L	4	75	C	10	80,8	1,61	2,03	8,9	1374015	96,69
74	Amygdala	1	Psilocybin	(…)	R	A_1P(…)R	1	3	C	1	18,7	1,1	2,04	8,8	8433028	95,42
75	Amygdala	2	Psilocybin	(…)	L	A_2P(…)L	1	18	B	3	56,3	1,69	2,15	9,2	1159574	93,47
76	Amygdala	2	Saline	(…)	R	A_2S(…)R	3	68	D	9	61,6	1,79	2,12	9,3	N/A	N/A
77	Amygdala	3	Saline	(…)	L	A_3S(…)L	1	9	A	2	20,7	1,28	2,13	9,4	18790729	94
78	Amygdala	3	Psilocybin	(…)	R	A_3P(…)R	2	47	G	6	42,4	1,61	2,11	9,4	6903376	95,19
79	Amygdala	4	Psilocybin	(…)	L	A_4P(…)L	1	7	G	1	59,2	1,71	2,11	9,3	12799848	93,94
80	Amygdala	4	Saline	(…)	R	A_4S(…)R	4	81	A	11	46,7	1,42	2,03	9,3	5957305	92,99
81	Amygdala	5	Saline	(…)	L	A_5S(…)L	3	50	B	7	41,9	1,68	2,14	9,5	9695885	92,6
82	Amygdala	5	Psilocybin	(…)	R	A_5P(…)R	3	72	H	9	72,1	1,87	2,14	9,1	4037746	95,18
83	Amygdala	6	Saline	(…)	L	A_6S(…)L	3	51	C	7	29,1	1,55	2,19	9,1	1230618	95,31
84	Amygdala	6	Psilocybin	(…)	R	A_6P(…)R	1	10	B	2	52,2	1,58	2,15	9,1	7337003	91,1
85	Amygdala	7	Psilocybin	(…)	L	A_7P(…)L	2	25	A	4	40,4	1,52	2,07	9,1	6656100	93,07
86	Amygdala	7	Saline	(…)	R	A_7S(…)R	1	22	F	3	78,9	1,85	2,13	9	2228582	95,27
87	Amygdala	8	Saline	(…)	L	A_8S(…)L	3	60	D	8	62,7	1,79	2,12	9,5	4900776	90,83
88	Amygdala	8	Psilocybin	(…)	R	A_8P(…)R	1	17	A	3	32,3	1,49	2,18	9,3	7821269	88,34
89	Amygdala	9	Psilocybin	(…)	L	A_9P(…)L	1	8	H	1	78,6	1,8	2,12	9	6337737	91,54
90	Amygdala	9	Saline	(…)	R	A_9S(…)R	4	80	H	10	40,6	1,5	2,03	8,7	1176849	93,87
91	Amygdala	10	Saline	(…)	L	A_10S(…)L	2	36	D	5	79,1	1,77	2,09	9,5	191545	95,3
92	Amygdala	10	Psilocybin	(…)	R	A_10P(…)R	4	83	C	11	32,3	1,21	1,99	8,7	3626926	95,71
93	Amygdala	11	Psilocybin	(…)	L	A_11P(…)L	4	89	A	12	35,4	1,25	2,02	9,6	12365043	91,35
94	Amygdala	11	Saline	(…)	R	A_11S(…)R	3	64	H	8	53,1	1,77	2,14	9,1	18982711	96,03
96	Amygdala	12	Psilocybin	(…)	R	A_12P(…)R	4	85	E	11	7,7	N/A	N/A	8,7	4940100	90,32
ENTC	-	-	-	-	-	-	1	24	H	3	1,1	0,15	4,84	1	6275321	94,44

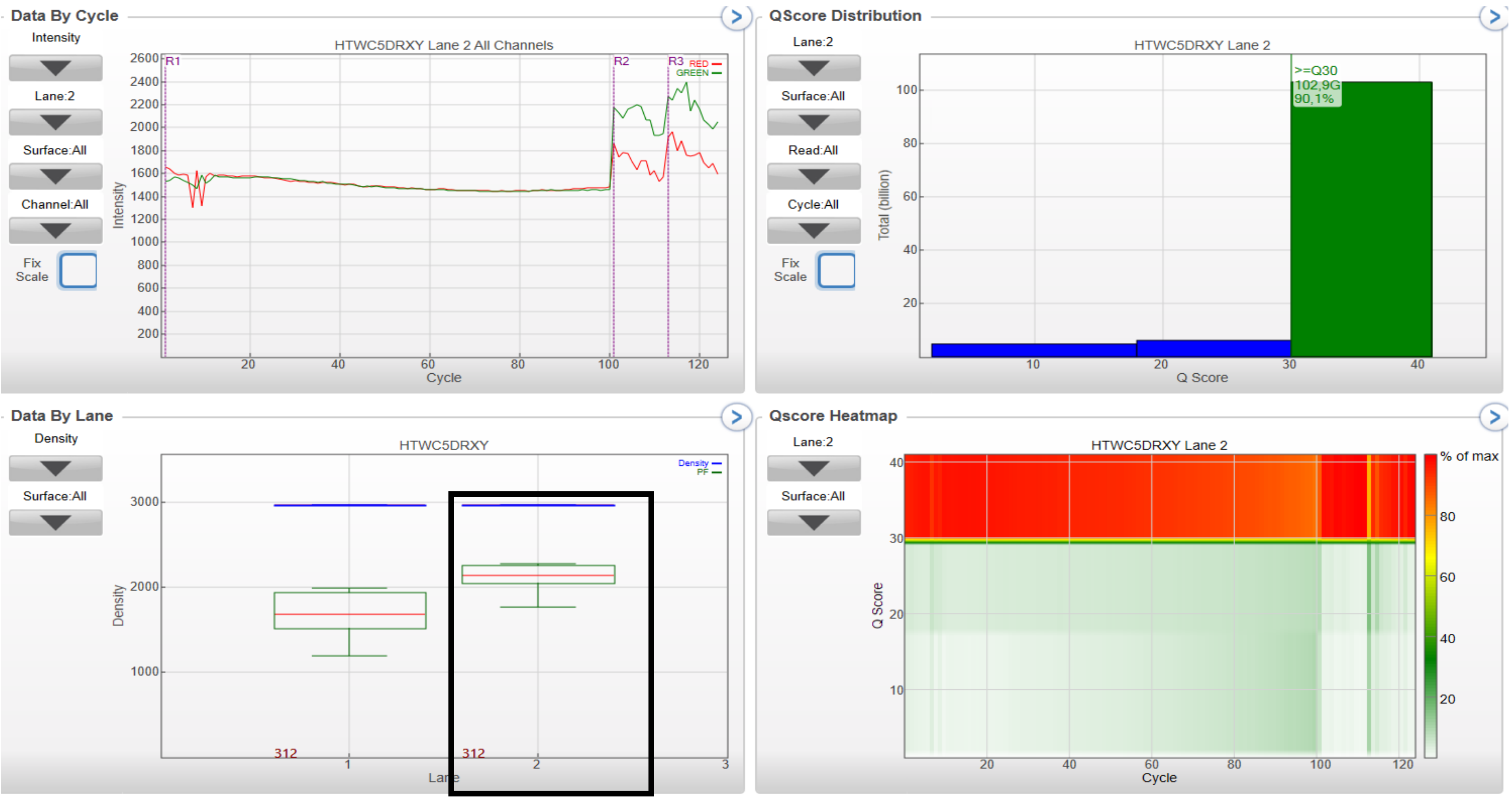
Data By Cycle
Intensity
Lane:2
Surface:All
Channel:All
Fix Scale
HTWC5DRXY Lane 2 All Channels
R1
R2
R3
RED
GREEN
Intensity
Cycle
QScore Distribution
Lane:2
Surface:All
Read:All
Cycle:All
Fix Scale
HTWC5DRXY Lane 2
>=Q30
102,9G
90,1%
Total (billion)
Q Score
Data By Lane
Density
Surface:All
HTWC5DRXY
Density
PF
312
312
Lane
Qscore Heatmap
Lane:2
Surface:All
HTWC5DRXY Lane 2
% of max
Q Score
Cycle

Cycle Status

Extracted	124	Called	124	Scored	124	Error Rated	0 - 100

Run Summary

Level	Yield Total (G)	Projected Total Yield (G)	Aligned (%)	Error Rate (%)	Intensity Cycle 1	% >= Q30
Read 1	164,77	164,77	28,21	0,34	1630	87,95
Non-Indexed Total	164,77	164,77	28,21	0,34	1630	87,95
Total	201,38	201,38	28,21	0,34	1776	88,17

Read 1

Lane	Tiles	Density (K/mm2)	Clusters PF (%)	Legacy Phasing/Prephasing rate	Phasing slope/offset	Prephasing slope/offset	Cluster Count Raw (M)	Cluster Count PF (M)	% >= Q30	Yield(G)	Cycles Err Rated	Aligned (%)	Error Rate (%)	Error Rate 35 cycle (%)	Error Rate 75 cycle (%)	Error Rate 100 cycle (%)	Intensity Cycle 1
2	312	2961 ± 0	72,12 ± 3,88	0,074 / 0,031	0,100 / 1,149	0,063 / -0,054	1276,67	920,76	89,71	91,19	0 - 100	35,59 ± 0,79	0,21 ± 0,03	0,13 ± 0,02	0,19 ± 0,02	0,21 ± 0,03	1660 ± 130

Well	Sample ID	Range	ng/uL	% Total	nmole/L	Avg. Size	%CV	Threshold (	DQN
A1	18	1	4,5434	99,9	26,1996	285	19,25	300	3,3
B1	79	1	6,3852	99,7	34,292	306	23,32	300	4,3
C1	77	1	0,4127	79,6	2,0784	327	28,58	300	4,2
D1	88	1	1,7892	99,7	10,0274	293	19,39	300	3,8
E1:	75	1	3,8953	98	19,7191	325	22,48	300	5,5
F1:	86	1	5,0978	98,8	26,6056	315	24,18	300	4,8
G1:	36	1	18,9468	100	104,0379	300	19,92	300	4,2
H1:	91	1	12,3757	97,1	63,6649	320	21,65	300	5,1
A2	26	2	21,4596	100	112,2251	315	19,39	300	5,2
B2	32	2	10,9173	96,9	57,8479	310	23,37	300	4,5
C2	83	2	1,7077	99,7	8,5702	328	25,15	300	5,3
D2	23	2	2,2399	99	12,0479	306	20,54	300	4,6
E2:	65	2	2,1972	98,5	11,6745	310	22,96	300	4,6
F2:	59	2	1,299	93,9	6,8369	313	20,7	300	4,9
G2:	51	2	5,3389	98,1	27,0068	325	23,56	300	5,4
H2:	94	2	3,2495	97,8	16,8166	318	20,37	300	5,3
A3	20	3	3,7669	100	20,4888	302	20,77	300	4,2
B3	29	3	10,5862	99,5	54,426	320	23,19	300	5,1
C3	76	3	4,6163	99	22,4515	338	25,91	300	5,8
D3:	38	3	1,7043	97,4	8,6156	325	27,31	300	5,1
E3:	69	3	5,0029	99,1	25,5146	323	23,56	300	5,1
F3:	35	3	1,9337	94,9	10,0004	318	22,86	300	4,9
G3:	66	3	2,0728	98,4	10,5589	323	22,87	300	5,2
H3:	Neg	3	0,0093	83,6	0,0608		33,35	300	less than lo
A4	80	4	10,3201	99,8	60,6564	280	17,08	300	2,8
B4	92	4	7,118	99,2	35,7687	327	23,93	300	0,3
C4	14	4	5,3035	99,2	24,9782	349	24,71	300	less than lo
A5	1	5	4,009	92,5	23,1538	285	20,92	300	3,3
B5	43	5	7,2524	98,9	37,8036	316	23,61	300	4,9
C5:	45	5	20,3756	97,5	90,7269	369	25,81	300	7,4
D5:	87	5	3,2328	99,5	17,2601	308	19,64	300	4,9
E5:	5	5	12,3488	97,7	62,2762	326	24,87	300	5,5
F5:	49	5	4,2343	98,2	22,9289	304	24,23	300	4,1
G5:	POS	5	0,0656	82,5	0,6176		3,21	300	0,1
