# Supplementary material for: Psilocybin elicits a conserved glucocorticoid-responsive gene signature across five 5-HT2A receptor-rich brain regions in rat
Source: Acta Neuropsychiatr. 2026 Apr 10;38:e37. doi: 10.1017/neu.2026.10075 (PMC13202413; doi:10.1017/neu.2026.10075)
Supplement: Veysi et al. supplementary material 5 — Veysi et al. supplementary material [file S0924270826100751sup005.pdf]

Supplement V

TATAA BIOCENTER

PROJECT 101 005 - 24 samples QuantSeq 3'mRNA Sequencing of rat hipp

RESULT FILES

Information - Download verification

File (Number)	Analysis
raw_multiqc_report*.html (2)	Pre-processing and QC
trim_multiqc_report*.html (1)	Alignment
	Alignment
QC.cumulative.gene.assignment.geneboby.pdf (1)	Alignment
*001_read_counts.txt (22)	Alignment
	DGE
group_condition*annotated.txt (1)	DGE
PCA.svg (1)	
MA*.svg (1)	DGE
gsea*results*.txt (4)	Pathway analysis
gsea*dotplot*.svg (4)	
gsea*cnet*.svg (4)	Pathway analysis

FASTQ

Information - Download verification

Extraction ID	md5sum	File
Sample 1	731165b02506336074bcd524b8d4c01a	1_S1_L001_R1_001.fastq.gz
Sample 1	ce8cd5cb3ab734bdc5b13b6c126a3bb1	1_S1_L002_R1_001.fastq.gz
Sample 1	1d6ceef680032d5b7bc1b09d7dd02563	1_S1_L003_R1_001.fastq.gz
Sample 1	ae4940b38ebcc7c661e3e14105774f7e	1_S1_L004_R1_001.fastq.gz
Sample 2	1f5556f9b2b8f10e80916defa6b7e61b	2_S2_L001_R1_001.fastq.gz
Sample 2	87b7c4c042929a187ece42062dca6cd5	2_S2_L002_R1_001.fastq.gz
Sample 2	7257a529e5f5679185c69b0ceea6e18c	2_S2_L003_R1_001.fastq.gz
Sample 2	13e9894385d58b46d3b913f5a2bfc36f	2_S2_L004_R1_001.fastq.gz
Sample 3	e4b66aeb28ed92374806c2bbb6e568a1	3_S3_L001_R1_001.fastq.gz
Sample 3	b215adacb6c56e5db3801cdc29f09db7	3_S3_L002_R1_001.fastq.gz
Sample 3	17c8f00681dcaf5ec2de01932179570c	3_S3_L003_R1_001.fastq.gz
Sample 3	025a17929f6ddf3a97e5e47716c589fb	3_S3_L004_R1_001.fastq.gz
Sample 4	dbe312cd94d15901a0b336e0bbbcd5d8	4_S8_L001_R1_001.fastq.gz
Sample 4	4fdb8771fb5de37dae54e2901a26e4d1	4_S8_L002_R1_001.fastq.gz
Sample 4	e9183d6543ba703fd47b353311bdb0d0	4_S8_L003_R1_001.fastq.gz
Sample 4	47cab26a6527c012ef014629c713b3a4	4_S8_L004_R1_001.fastq.gz
Sample 5	168fbd1d7745743e47fd49f96e40e871	5_S4_L001_R1_001.fastq.gz
Sample 5	a6b649c76bf4c42de913b2f7c261f929	5_S4_L002_R1_001.fastq.gz
Sample 5	e3d79d02cd1f805d45ff6f87b5258a5e	5_S4_L003_R1_001.fastq.gz
Sample 5	aabffce6802232ad061594170d25d7bc	5_S4_L004_R1_001.fastq.gz
Sample 7	5d8edd9788fab02b6c7e946e135c48eb	7_S6_L001_R1_001.fastq.gz
Sample 7	93b557af4196d0d1297c797bff060053	7_S6_L002_R1_001.fastq.gz
Sample 7	b1602fcc07dcb7bdcaebc9b228094b92	7_S6_L003_R1_001.fastq.gz
Sample 7	04697cfc1a43cf145d61b39ae6490569	7_S6_L004_R1_001.fastq.gz
Sample 8	700f8bcc95a3058ffece505a997a4434	8_S7_L001_R1_001.fastq.gz
Sample 8	0b08392384fa31d1b365a40d62aa1fcd	8_S7_L002_R1_001.fastq.gz
Sample 8	794da5c38ad1ce00ee37b8071219527e	8_S7_L003_R1_001.fastq.gz
Sample 8	4299934f24ec3ada3f8752787cc70d2e	8_S7_L004_R1_001.fastq.gz
Sample 9	231e9d8a8e2c36418fff74319b0c4717	9_S5_L001_R1_001.fastq.gz
Sample 9	c56c1dd1863a302059b7122046e49f22	9_S5_L002_R1_001.fastq.gz
Sample 9	57a33048f9c7ea3bc932bc6e6b724c4f	9_S5_L003_R1_001.fastq.gz
Sample 9	a8282573e73e0632942c61313af74b6d	9_S5_L004_R1_001.fastq.gz
Sample 10	20f6ba71498c71fd97a06759cf9a737a	10_S9_L001_R1_001.fastq.gz
Sample 10	6e7e2140b38c6f7706cadf3e6032ae04	10_S9_L002_R1_001.fastq.gz
Sample 10	27d7a706a32d4b2d1afbeed93641f5a2	10_S9_L003_R1_001.fastq.gz
Sample 10	cdfaeea89da6eceefd56c7fcba44e0a2	10_S9_L004_R1_001.fastq.gz
Sample 11	28f6dace9353f1753656b644fc6fb1da	11_S10_L001_R1_001.fastq.gz
Sample 11	e502c41be7eae57717d855194cbaf8d3	11_S10_L002_R1_001.fastq.gz
Sample 11	efa6400945227ef3b99423e16a614db3	11_S10_L003_R1_001.fastq.gz
Sample 11	91930c81bdd03484b0407329460e70ed	11_S10_L004_R1_001.fastq.gz
Sample 12	915c6c17258adab2802e873813d936bf	12_S11_L001_R1_001.fastq.gz
Sample 12	096e332927c0515352a3887b1786eba0	12_S11_L002_R1_001.fastq.gz
Sample 12	23999dd72caeddd5df5dc9c02d95b084	12_S11_L003_R1_001.fastq.gz
Sample 12	5f4b0c5e9a739d34ced1d3db9ecf315f	12_S11_L004_R1_001.fastq.gz
Sample 13	3d3bbdb4aa8b6c469ecb3fea6d984ca7	13_S12_L001_R1_001.fastq.gz
Sample 13	a33b740056d6542d84e806dc51b79df8	13_S12_L002_R1_001.fastq.gz
Sample 13	575f9792ddda6b09d7670d777f720a37	13_S12_L003_R1_001.fastq.gz
Sample 13	39b7e23058e08ae024806dd7e6c9302c	13_S12_L004_R1_001.fastq.gz
Sample 14	21103eecaee155656160aed8d04cd5d6	14_S13_L001_R1_001.fastq.gz
Sample 14	ddfa3dc3eb51dad339b5ce315881c56b	14_S13_L002_R1_001.fastq.gz
Sample 14	dc1d2e130940f22ffe9c761fc78d43cb	14_S13_L003_R1_001.fastq.gz
Sample 14	cb9df92e06c0ad155562f393313e3b0a	14_S13_L004_R1_001.fastq.gz
Sample 15	76d468de630ac531ccff83142bc719a3	15_S14_L001_R1_001.fastq.gz
Sample 15	8b67d37a19b4e4c2fc746d7beabe18ee	15_S14_L002_R1_001.fastq.gz
Sample 15	aee954149fcbbc22942a797561da28a9	15_S14_L003_R1_001.fastq.gz

Sample 15	e01a6da8b7fed3ba12b92b5dbc3ecbd2	15_S14_L004_R1_001.fastq.gz
Sample 16	6449dbd378eba8c4bdef6b979ce8a323	16_S15_L001_R1_001.fastq.gz
Sample 16	08c238b0cafb410df522f65de9baabf7	16_S15_L002_R1_001.fastq.gz
Sample 16	19e0b0b420a434836ef57d5a809602bd	16_S15_L003_R1_001.fastq.gz
Sample 16	ddacd9c2c59849bc17fc8e88e460d318	16_S15_L004_R1_001.fastq.gz
Sample 17	78563cff033e1142dd07aa7921c9ff1b	17_S16_L001_R1_001.fastq.gz
Sample 17	ebde92730ab99166a5e0eeb5b1e01397	17_S16_L002_R1_001.fastq.gz
Sample 17	08ab21cb2a5139fa1f8474b48b8cc64c	17_S16_L003_R1_001.fastq.gz
Sample 17	fb92ad258dee32cf5ac9e6e45da4579f	17_S16_L004_R1_001.fastq.gz
Sample 18	bc9927673bfcb7c48a00341cc12d2b34	18_S17_L001_R1_001.fastq.gz
Sample 18	4f319a0e78aec1197a4ce8ed2b205429	18_S17_L002_R1_001.fastq.gz
Sample 18	08a87a107d1e675c66b4cbd9f3b4dcd6	18_S17_L003_R1_001.fastq.gz
Sample 18	0ff73f88ce7429c9199830845c944e18	18_S17_L004_R1_001.fastq.gz
Sample 20	da4fe6bce4fd25dcd54254f591f3f50b	20_S18_L001_R1_001.fastq.gz
Sample 20	c56980b50fc785de7b09472c471a555a	20_S18_L002_R1_001.fastq.gz
Sample 20	9f304aad200a1a15e498315ec8dcdfd4	20_S18_L003_R1_001.fastq.gz
Sample 20	1c04cf827abfb7451568823e5fb3ecef	20_S18_L004_R1_001.fastq.gz
Sample 21	fa9052f64b24517bacb5ab518da4e976	21_S19_L001_R1_001.fastq.gz
Sample 21	34a39933d9921aec549c1e3885f1461a	21_S19_L002_R1_001.fastq.gz
Sample 21	f9684201d03f20139f65744d246b710f	21_S19_L003_R1_001.fastq.gz
Sample 21	79375f04dc26b138179f5dd5a5c3191d	21_S19_L004_R1_001.fastq.gz
Sample 22	81a3c6c63480d8534c66cc98d9dde327	22_S20_L001_R1_001.fastq.gz
Sample 22	3a9cdad9acf39040e529ed5ec9746dd8	22_S20_L002_R1_001.fastq.gz
Sample 22	f5f00634076534291fe1583db7782f29	22_S20_L003_R1_001.fastq.gz
Sample 22	4df4555696fa9fc6c075ad5e6698a7fa	22_S20_L004_R1_001.fastq.gz
Sample 23	917af86a9e594c281b32a304e6f8a353	23_S21_L001_R1_001.fastq.gz
Sample 23	3ad905850220ebe288209119a6d9014b	23_S21_L002_R1_001.fastq.gz
Sample 23	a36f40b501c65db1956c9d47af839be2	23_S21_L003_R1_001.fastq.gz
Sample 23	35bdfbd95c90c4ced09fbb104d4bb6d5	23_S21_L004_R1_001.fastq.gz
Sample 24	978cfa74f4e9e08c74db665f39abbf0a	24_S22_L001_R1_001.fastq.gz
Sample 24	0bce853172dd9c4eea736d9263fd6823	24_S22_L002_R1_001.fastq.gz
Sample 24	20fcec0046bb46fe6f52edb8fa0bc470	24_S22_L003_R1_001.fastq.gz
Sample 24	c1e90993b275f5abc6885844187a70df	24_S22_L004_R1_001.fastq.gz

PROJECT 101 005 - 24 samples QuantSeq 3'mRNA Sequencing of rat hippocampus

SAMPLE LIST

Information table

TATAA ID	Sponsor ID	Experimental Groups	Comment
Sample 1	1SHL	Saline	
Sample 2	1PHR	Psilocybin	
Sample 3	2PHL	Psilocybin	
Sample 4	2SHR	Saline	
Sample 5	3SHL	Saline	
Sample 6	3PHR	Psilocybin	Sample lost in extraction process
Sample 7	4PHL	Psilocybin	
Sample 8	4SHR	Saline	
Sample 9	5SHL	Saline	
Sample 10	5PHR	Psilocybin	
Sample 11	6SHL	Saline	
Sample 12	6PHR	Psilocybin	
Sample 13	7PHL	Psilocybin	
Sample 14	7SHR	Saline	
Sample 15	8SHL	Saline	
Sample 16	8PHR	Psilocybin	
Sample 17	9PHL	Psilocybin	
Sample 18	9SHR	Saline	
Sample 19	10SHL	Saline	Sample lost in extraction process
Sample 20	10PHR	Psilocybin	
Sample 21	11PHL	Psilocybin	
Sample 22	11SHR	Saline	
Sample 23	12SHL	Saline	
Sample 24	12PHR	Psilocybin	

PROJECT 101 005 - 24 samples QuantSeq 3'mRNA Sequencing of rat hippocampus

EXTRACTION QUALITY CONTROL

Result table

TATAA ID	Sponsor ID	Concentration (ng/ul)	A260/230	A260/280	RQN
Sample 1	1SHL	204,0	0,2	2,1	7,9
Sample 2	1PHR	259,8	1,5	2,1	10,0
Sample 3	2PHL	421,0	2,0	2,0	9,9
Sample 4	2SHR	73,0	1,5	2,0	10,0
Sample 5	3SHL	220,8	2,0	2,0	10,0
Sample 7	4PHL	229,2	2,0	2,1	10,0
Sample 8	4SHR	216,8	1,9	2,1	10,0
Sample 9	5SHL	265,4	2,0	2,1	10,0
Sample 10	5PHR	245,1	0,6	2,1	10,0
Sample 11	6SHL	58,0	1,3	2,0	10,0
Sample 12	6PHR	273,5	1,9	2,1	9,0
Sample 13	7PHL	84,3	0,2	2,1	9,5
Sample 14	7SHR	420,3	2,1	2,0	9,9
Sample 15	8SHL	50,8	0,5	2,0	10,0
Sample 16	8PHR	331,3	2,0	2,1	9,8
Sample 17	9PHL	50,7	1,5	2,0	10,0
Sample 18	9SHR	55,6	0,5	2,1	10,0
Sample 20	10PHR	251,9	1,9	2,1	9,4
Sample 21	11PHL	428,7	2,2	2,1	10,0
Sample 22	11SHR	155,6	0,7	2,1	10,0
Sample 23	12SHL	386,6	2,3	2,1	10,0
Sample 24	12PHR	107,2	1,5	2,0	10,0

THRESHOLD LEVELS

TATAA Acceptance Criteria

Values outside thresholds are reported in red (A260/230 < 1.2)

Values outside thresholds are reported in red (A260/280 < 1.7)

Values outside thresholds are reported in orange (RQN < 7)

PROJECT 101 005 - 24 samples QuantSeq 3'mRNA Sequencing of rat hippocampus

TEMPERATURE PROTOCOL CYCLE DETERMINATION qPCR I

Information - Thermal Cycling

Step		Temperature	Duration	Cycles
Activation		98°C	30 s	1
Cycling				
	Denaturation	98°C	10 s	
	Annealing	65°C	20 s	35
	Elongation	72°C	30 s	
Extension				
		72°C	1 min	1
		10°C	∞	

MASTERMIX SETUP qPCR

Information - Mastermix protocol

Reagents	Stock conc.	Volume (µl)	Final conc.
Primers (7000)	-	5	-
PCR mix	-	7	-
Enzyme (E)	-	1	-
SYBR Green I nucleic acid stain	2.5X	1,2	1X
Elution Buffer (EB)	-	14,1	-
Template	-	1,7	-
Total Reaction Volume		**30**	

TEMPERATURE PROTOCOL FOR QUANTIFICATION qPCR II

Information - Thermal Cycling

Step		Temperature	Duration	Cycles
Activation		95°C	1 min	1
Cycling				
	Denaturation	95°C	15 s	
	Annealing	60°C	15 s	14
	Elongation	72°C	60s	
Extension				
		72°C	1 min	1
		10°C	∞	

MASTERMIX SETUP qPCR

Information - Mastermix protocol

Reagents	Stock conc.	Volume (µl)	Final conc.
Unique Dual Index Primer Pairs	-	10	-
PCR mix (PM)	-	7	-
Enzyme (PE)	-	1	-
Template	-	17	-
Total Reaction Volume		**35**	

TEMPERATURE PROTOCOL LIBRARY QUANTIFICATION qPCR III

Information - Thermal Cycling

Step		Temperature	Duration	Cycles
Activation		95°C	1 m	1
Cycling				
	Denaturation	95°C	5 s	
	Annealing	60°C	30 s	35
	Elongation	72°C	10 s	
Extension				
	Denaturation	95°C	15 s	1
	Annealing	60°C	15 s	
	Melt	95°C	-	

MASTERMIX SETUP qPCR

Information - Mastermix protocol

Reagents	Stock conc.	Volume (µl)	Final conc.
Primers (Fwd + Rv)	10 µM	0,4	400 nM
TATAA SYBR GrandMaster mix Low Rox	2X	5	1X
RNAse-free water	-	2,6	-
Template	-	2	-
Total Reaction Volume		**10**	

PROJECT 101 005 - 24 samples QuantSeq 3'mRNA Sequencing of rat hippocampus

LIBRARY QUALITY CONTROL

Result table

TATAA ID	Sponsor ID	Average fragment length (bp)	Library Concentration (nM)
Sample 1	1SHL	255	11,2
Sample 2	1PHR	259	22,1
Sample 3	2PHL	260	10,9
Sample 4	2SHR	259	9,8
Sample 5	3SHL	266	10,1
Sample 7	4PHL	268	8,8
Sample 8	4SHR	286	7,0
Sample 9	5SHL	285	15,1
Sample 10	5PHR	268	7,9
Sample 11	6SHL	266	8,5
Sample 12	6PHR	265	19,6
Sample 13	7PHL	282	14,6
Sample 14	7SHR	284	5,6
Sample 15	8SHL	284	16,7
Sample 16	8PHR	283	1,3
Sample 17	9PHL	257	13,8
Sample 18	9SHR	288	18,7
Sample 20	10PHR	286	4,6
Sample 21	11PHL	283	10,2
Sample 22	11SHR	284	13,2
Sample 23	12SHL	263	4,2
Sample 24	12PHR	284	17,8

PROJECT 101 005 - 24 samples QuantSeq 3'mRNA Sequencing of rat hippocampus

SEQUENCING QUALITY CONTROL - SUMMARY

Result table

Density (K/mm2)	Cluster PF (%)	Cluster Count PF (M)	%>= Q30	Aligned PhiX (%)	PF Reads / Sample (M)
149	92,0	129	89,7	15,0	3,1

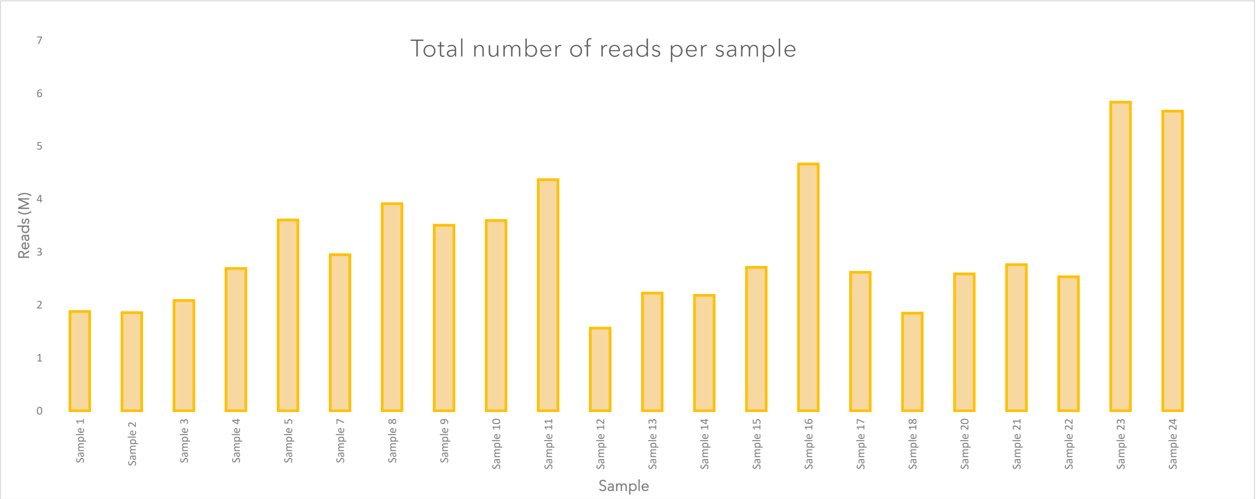

SEQUENCING QUALITY CONTROL

Result table

TATAA ID	Customer ID	Index i7	Index i5	% Reads Identified (PF)	Total number of Reads (M)
Sample 1	1SHL	CGGGAACCCGCA	GTCTTTGGCCCT	1,46	1,88
Sample 2	1PHR	AAACGTTCATCC	TTAGTAACTGGG	1,44	1,86
Sample 3	2PHL	TTGTCCGATATG	CAGAGCTTACAA	1,62	2,09
Sample 4	2SHR	ATCGACTTGTGT	ACACAATGCTAG	2,09	2,69
Sample 5	3SHL	CCAAAGAGGGAT	AGCCCGCGGGTT	2,80	3,61
Sample 7	4PHL	GAAGGGTAAAGC	TCGGGACCCGGC	2,29	2,95
Sample 8	4SHR	AGTCTCAGCAAA	GGGTCGTATACG	3,04	3,92
Sample 9	5SHL	TCCTCTCTTCTA	GATAATATATTA	2,72	3,51
Sample 10	5PHR	AACCCTGGGAAG	CGGCCCATTTGG	2,79	3,60
Sample 11	6SHL	AGGTGGTTCTAC	TGATAACCACCG	3,39	4,37
Sample 12	6PHR	TACGCCCACGTG	CACTGTTTCTGA	1,21	1,56
Sample 13	7PHL	GATTTCCCCCGA	GCTTTTTAAAGC	1,73	2,23
Sample 14	7SHR	CCCAATTTTGCC	TTCAAAAGGTTT	1,69	2,18
Sample 15	8SHL	TCAACAACCGGT	ACCTTGGTGGTA	2,10	2,71
Sample 16	8PHR	CAGATAATACGT	ACTGAGGAGCGT	3,62	4,67
Sample 17	9PHL	TATTGGCGGCCT	ATTCCTCCAAGA	2,03	2,62
Sample 18	9SHR	AGAGGCCGAACA	GTGGCTTCCGCG	1,43	1,85
Sample 20	10PHR	TGCTAAATTAGT	ACGAAGGGATCA	2,01	2,59
Sample 21	11PHL	CTATGCAAGCTG	CATCGGTCAGAT	2,14	2,76
Sample 22	11SHR	CCGGGCGTCATG	CAGTACTCCCTT	1,97	2,54
Sample 23	12SHL	TGGAGACTGGGC	TCCCATGCGCCA	4,52	5,84
Sample 24	12PHR	CTTACCGGGTAC	TGACCCTTGAAT	4,39	5,66
				Average:	3,08

THRESHOLD LEVELS

Sequencing depth

Values outside < 3 M reads are reported in red
