# Supplementary material for: Psilocybin elicits a conserved glucocorticoid-responsive gene signature across five 5-HT2A receptor-rich brain regions in rat
Source: Acta Neuropsychiatr. 2026 Apr 10;38:e37. doi: 10.1017/neu.2026.10075 (PMC13202413; doi:10.1017/neu.2026.10075)
Supplement: Veysi et al. supplementary material 6 — Veysi et al. supplementary material [file S0924270826100751sup006.pdf]

Supplement VI

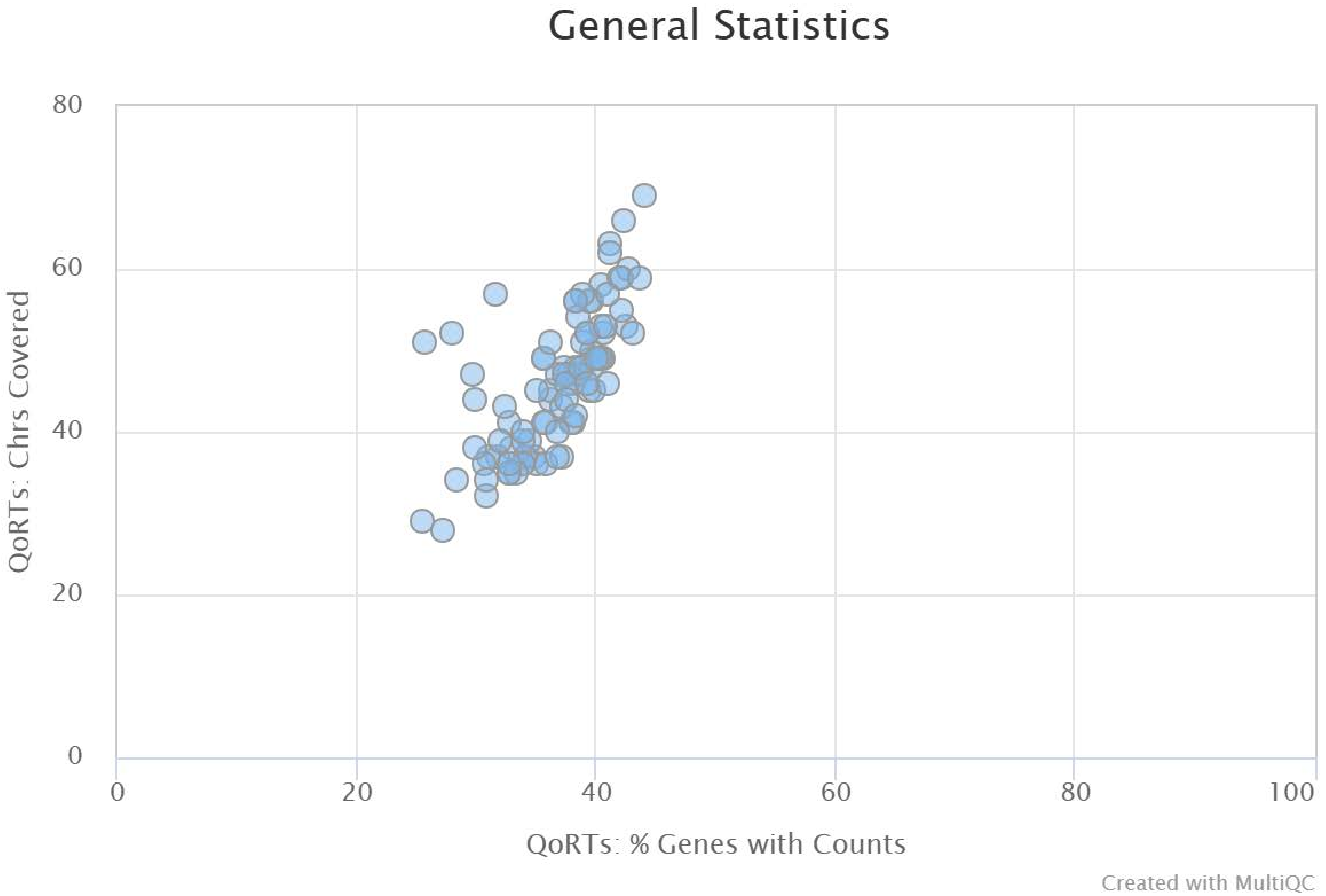

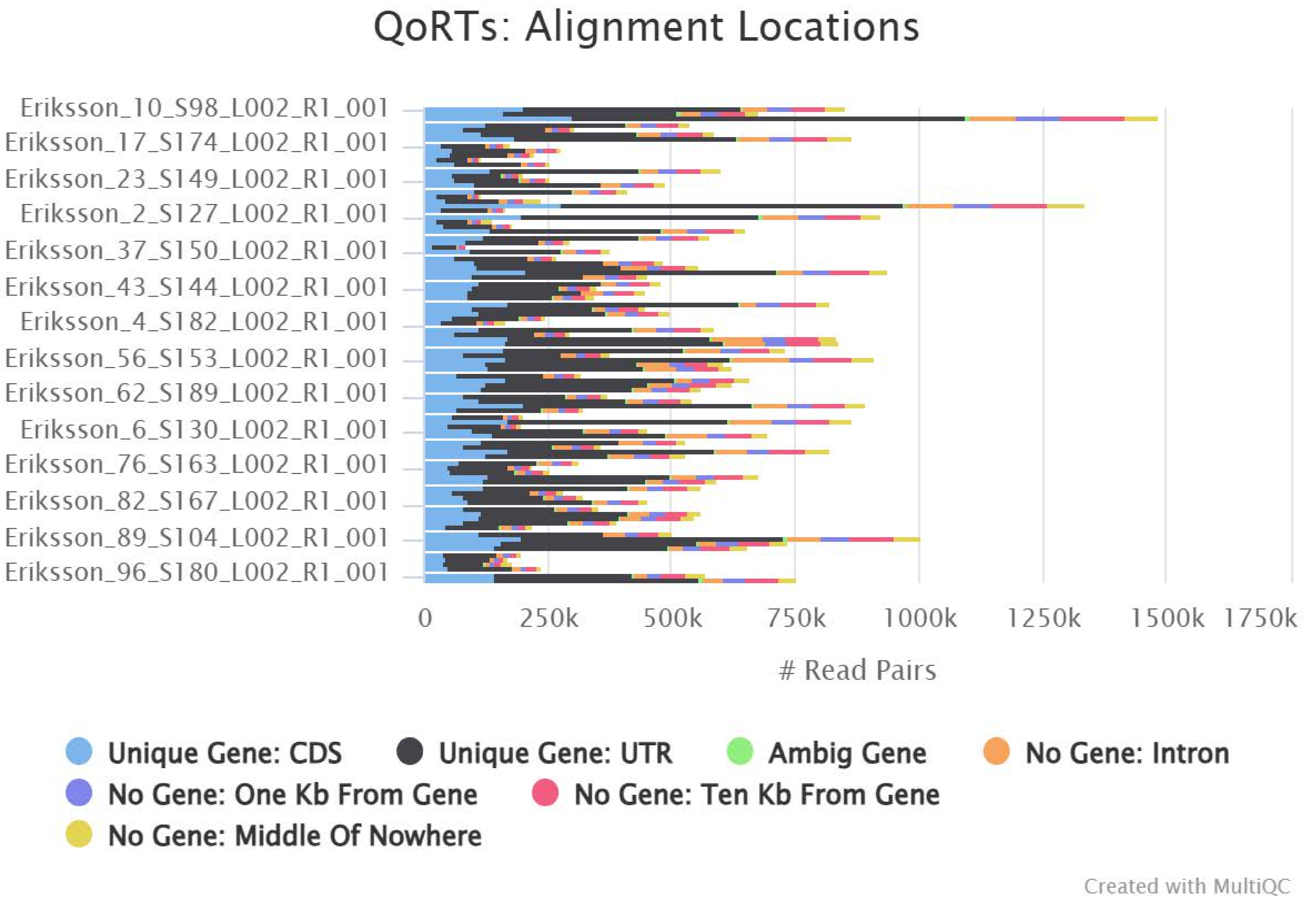

QoRTs: Alignment Locations
Eriksson_10_S98_L002_R1_001
Eriksson_17_S174_L002_R1_001
Eriksson_23_S149_L002_R1_001
Eriksson_2_S127_L002_R1_001
Eriksson_37_S150_L002_R1_001
Eriksson_43_S144_L002_R1_001
Eriksson_4_S182_L002_R1_001
Eriksson_56_S153_L002_R1_001
Eriksson_62_S189_L002_R1_001
Eriksson_6_S130_L002_R1_001
Eriksson_76_S163_L002_R1_001
Eriksson_82_S167_L002_R1_001
Eriksson_89_S104_L002_R1_001
Eriksson_96_S180_L002_R1_001
0
250k
500k
750k
1000k
1250k
1500k
1750k
Read Pairs
Unique Gene: CDS
Unique Gene: UTR
Ambig Gene
No Gene: Intron
No Gene: One Kb From Gene
No Gene: Ten Kb From Gene
No Gene: Middle Of Nowhere
Created with MultiQC

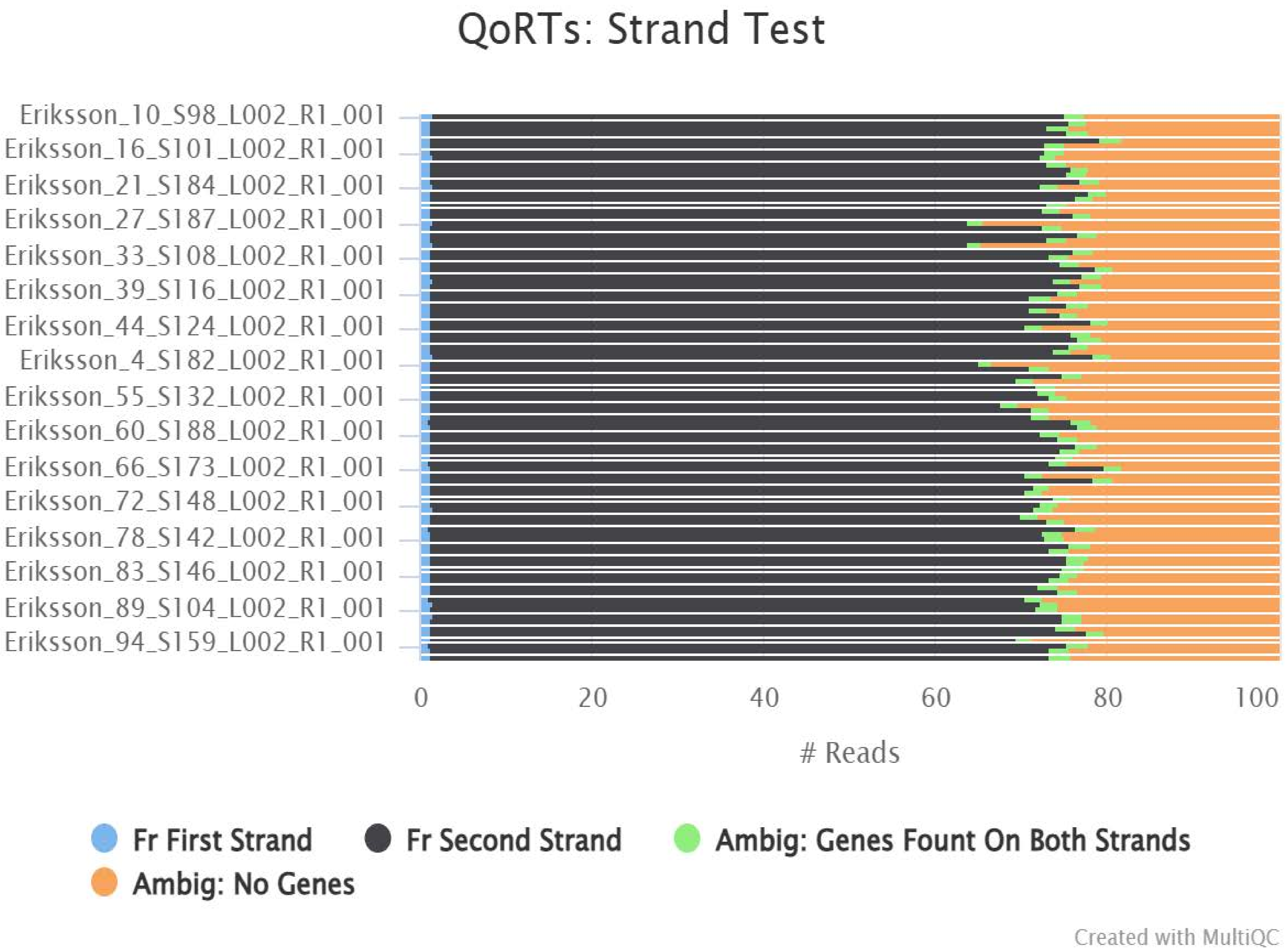
QoRTs: Strand Test
Eriksson_10_S98_L002_R1_001
Eriksson_16_S101_L002_R1_001
Eriksson_21_S184_L002_R1_001
Eriksson_27_S187_L002_R1_001
Eriksson_33_S108_L002_R1_001
Eriksson_39_S116_L002_R1_001
Eriksson_44_S124_L002_R1_001
Eriksson_4_S182_L002_R1_001
Eriksson_55_S132_L002_R1_001
Eriksson_60_S188_L002_R1_001
Eriksson_66_S173_L002_R1_001
Eriksson_72_S148_L002_R1_001
Eriksson_78_S142_L002_R1_001
Eriksson_83_S146_L002_R1_001
Eriksson_89_S104_L002_R1_001
Eriksson_94_S159_L002_R1_001
0
20
40
60
80
100
Reads
Fr First Strand
Fr Second Strand
Ambig: Genes Fount On Both Strands
Ambig: No Genes
Created with MultiQC
