# Supplementary material for: Psilocybin elicits a conserved glucocorticoid-responsive gene signature across five 5-HT2A receptor-rich brain regions in rat
Source: Acta Neuropsychiatr. 2026 Apr 10;38:e37. doi: 10.1017/neu.2026.10075 (PMC13202413; doi:10.1017/neu.2026.10075)
Supplement: Veysi et al. supplementary material 7 — Veysi et al. supplementary material [file S0924270826100751sup007.pdf]

Supplement VII

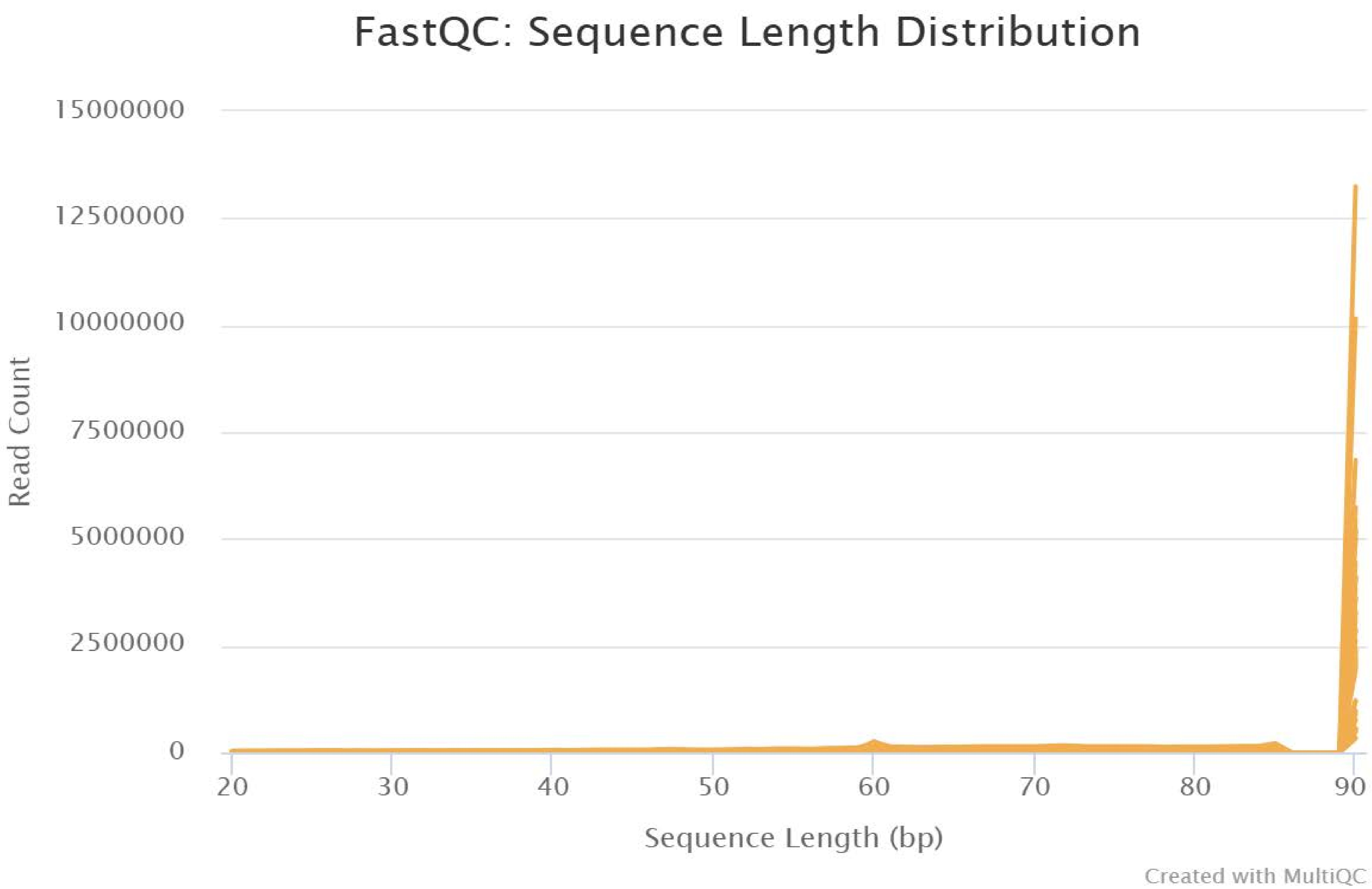

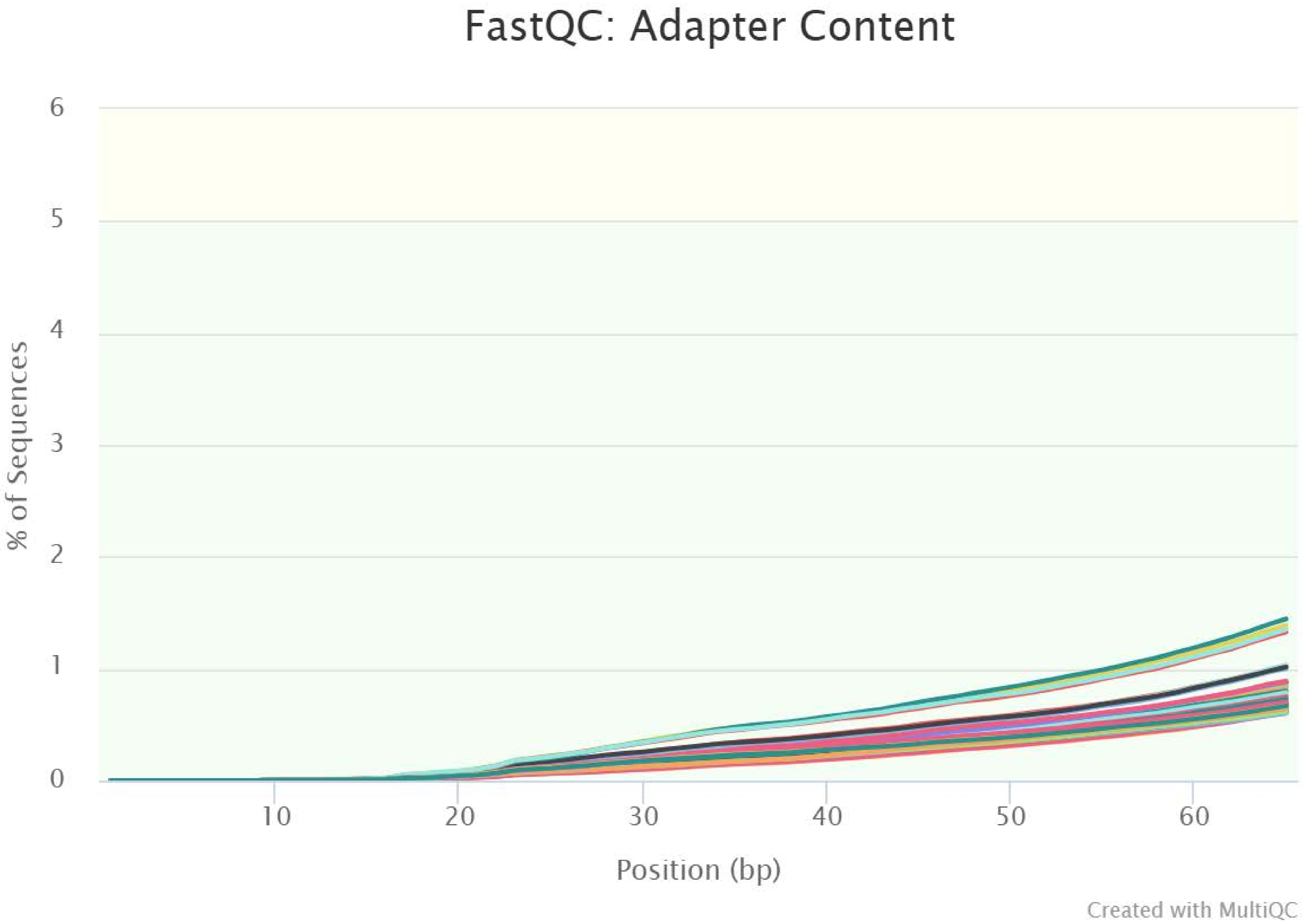

FastQC: Adapter Content
6
5
4
3
2
1
0
% of Sequences
10
20
30
40
50
60
Position (bp)
Created with MultiQC

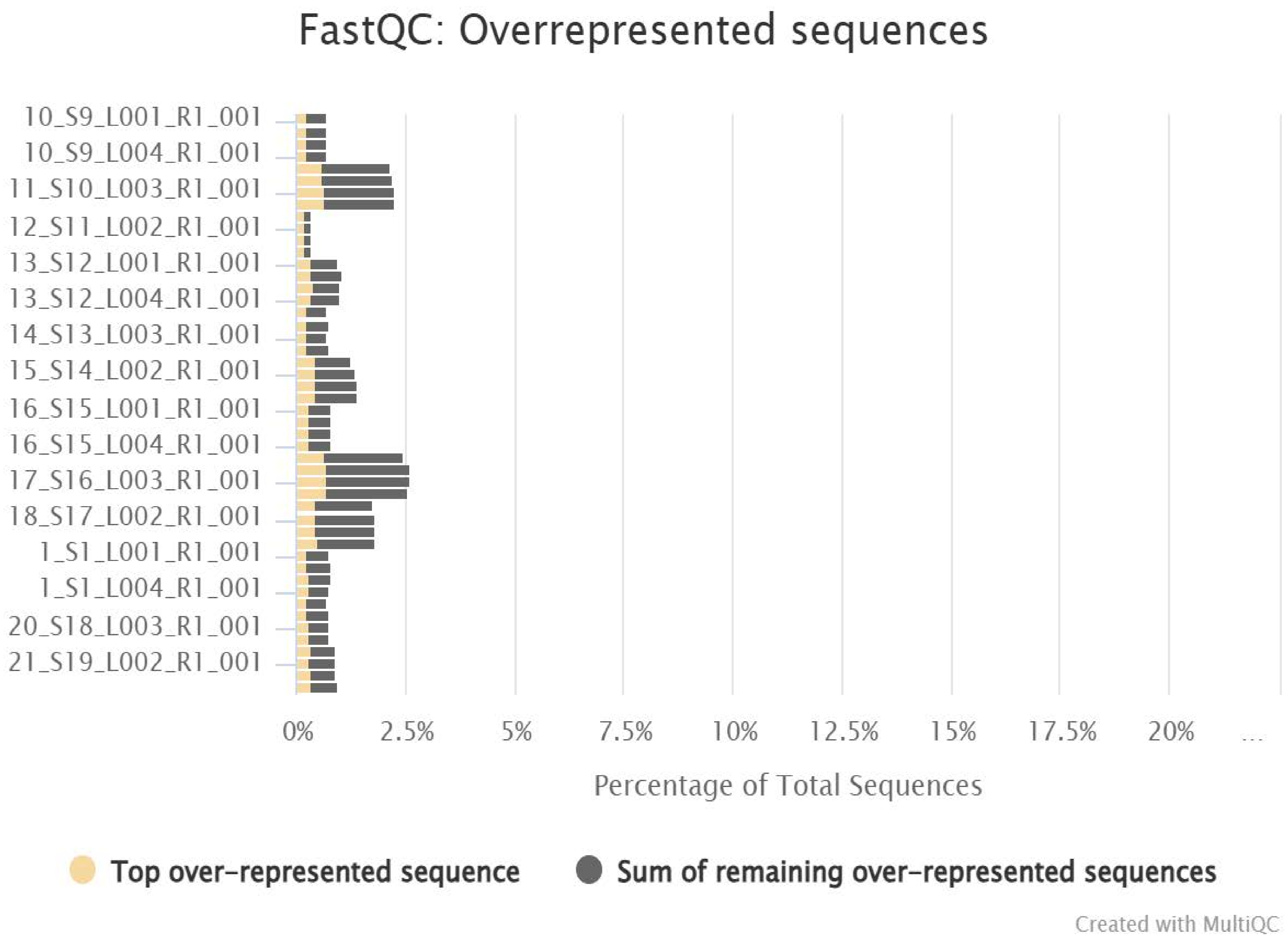
FastQC: Overrepresented sequences
10_S9_L001_R1_001
10_S9_L004_R1_001
11_S10_L003_R1_001
12_S11_L002_R1_001
13_S12_L001_R1_001
13_S12_L004_R1_001
14_S13_L003_R1_001
15_S14_L002_R1_001
16_S15_L001_R1_001
16_S15_L004_R1_001
17_S16_L003_R1_001
18_S17_L002_R1_001
1_S1_L001_R1_001
1_S1_L004_R1_001
20_S18_L003_R1_001
21_S19_L002_R1_001
0%
2.5%
5%
7.5%
10%
12.5%
15%
17.5%
20%
...
Percentage of Total Sequences
Top over-represented sequence
Sum of remaining over-represented sequences
Created with MultiQC

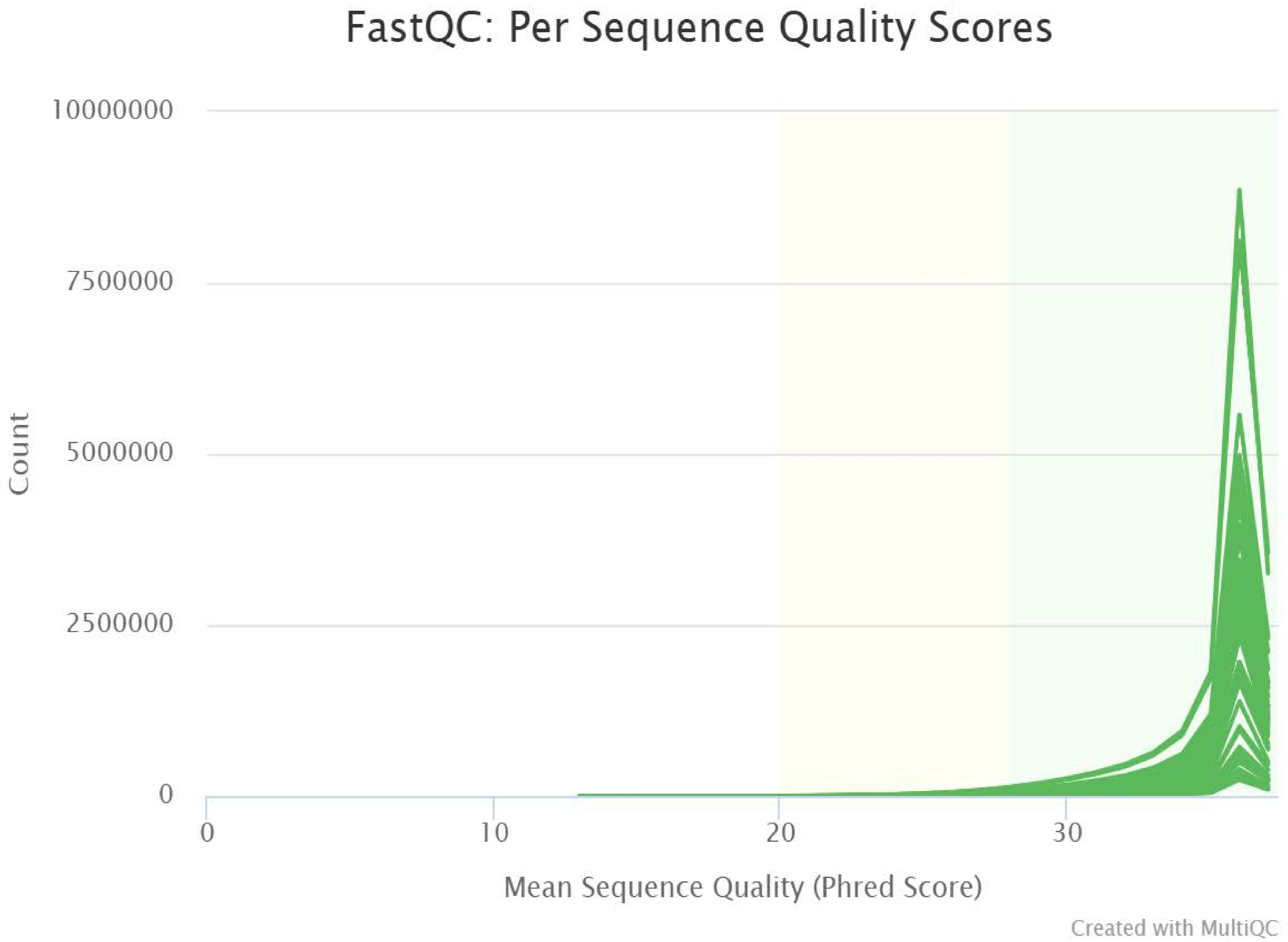
FastQC: Per Sequence Quality Scores
10000000
7500000
5000000
2500000
0
Count
0
10
20
30
Mean Sequence Quality (Phred Score)
Created with MultiQC

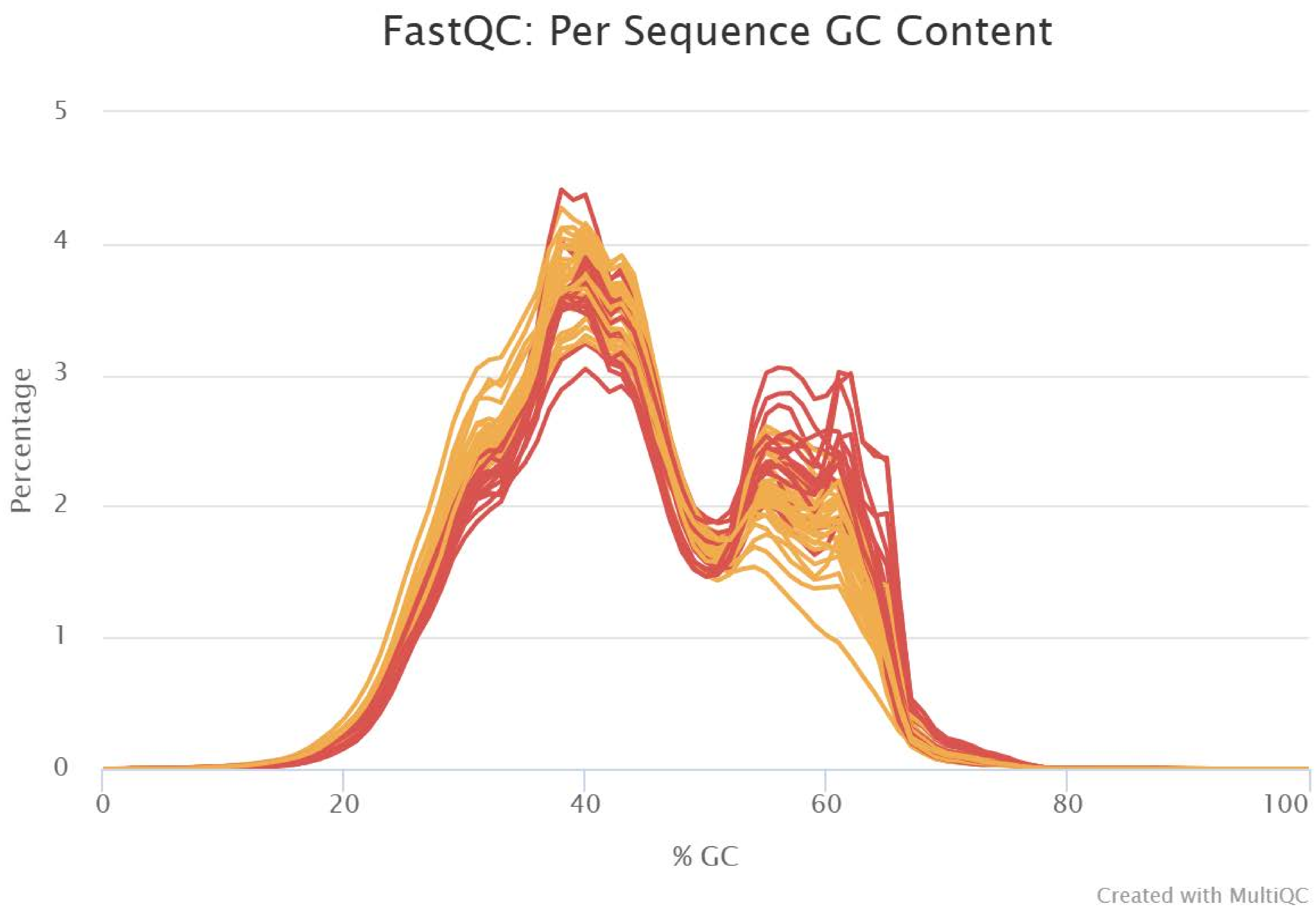
FastQC: Per Sequence GC Content
5
4
3
2
1
0
Percentage
0
20
40
60
80
100
% GC
Created with MultiQC

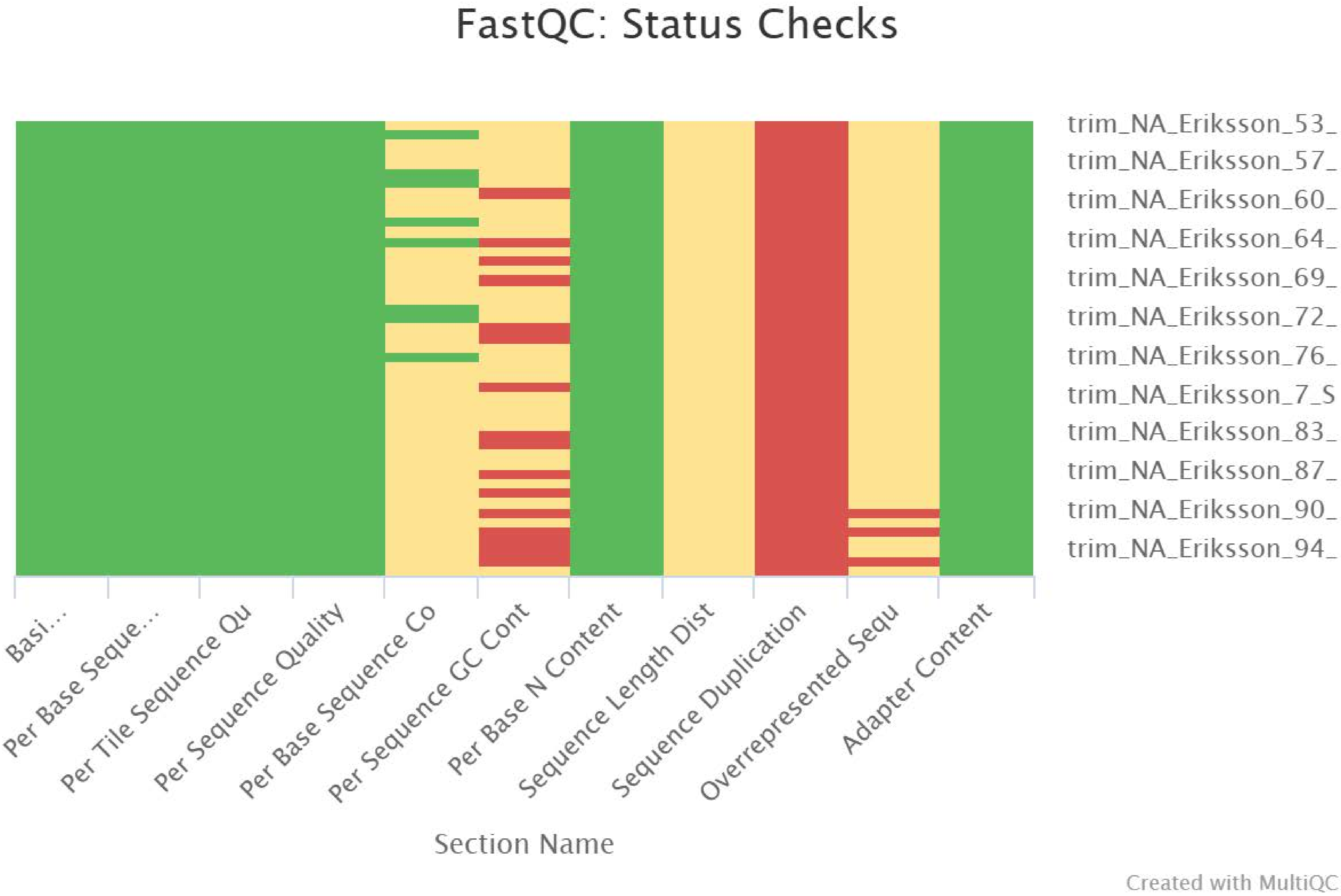

FastQC: Status Checks
trim_NA_Eriksson_53_
trim_NA_Eriksson_57_
trim_NA_Eriksson_60_
trim_NA_Eriksson_64_
trim_NA_Eriksson_69_
trim_NA_Eriksson_72_
trim_NA_Eriksson_76_
trim_NA_Eriksson_7_S
trim_NA_Eriksson_83_
trim_NA_Eriksson_87_
trim_NA_Eriksson_90_
trim_NA_Eriksson_94_
Basi...
Per Base Seque...
Per Tile Sequence Qu
Per Sequence Quality
Per Base Sequence Co
Per Sequence GC Cont
Per Base N Content
Sequence Length Dist
Sequence Duplication
Overrepresented Sequ
Adapter Content
Section Name
Created with MultiQC

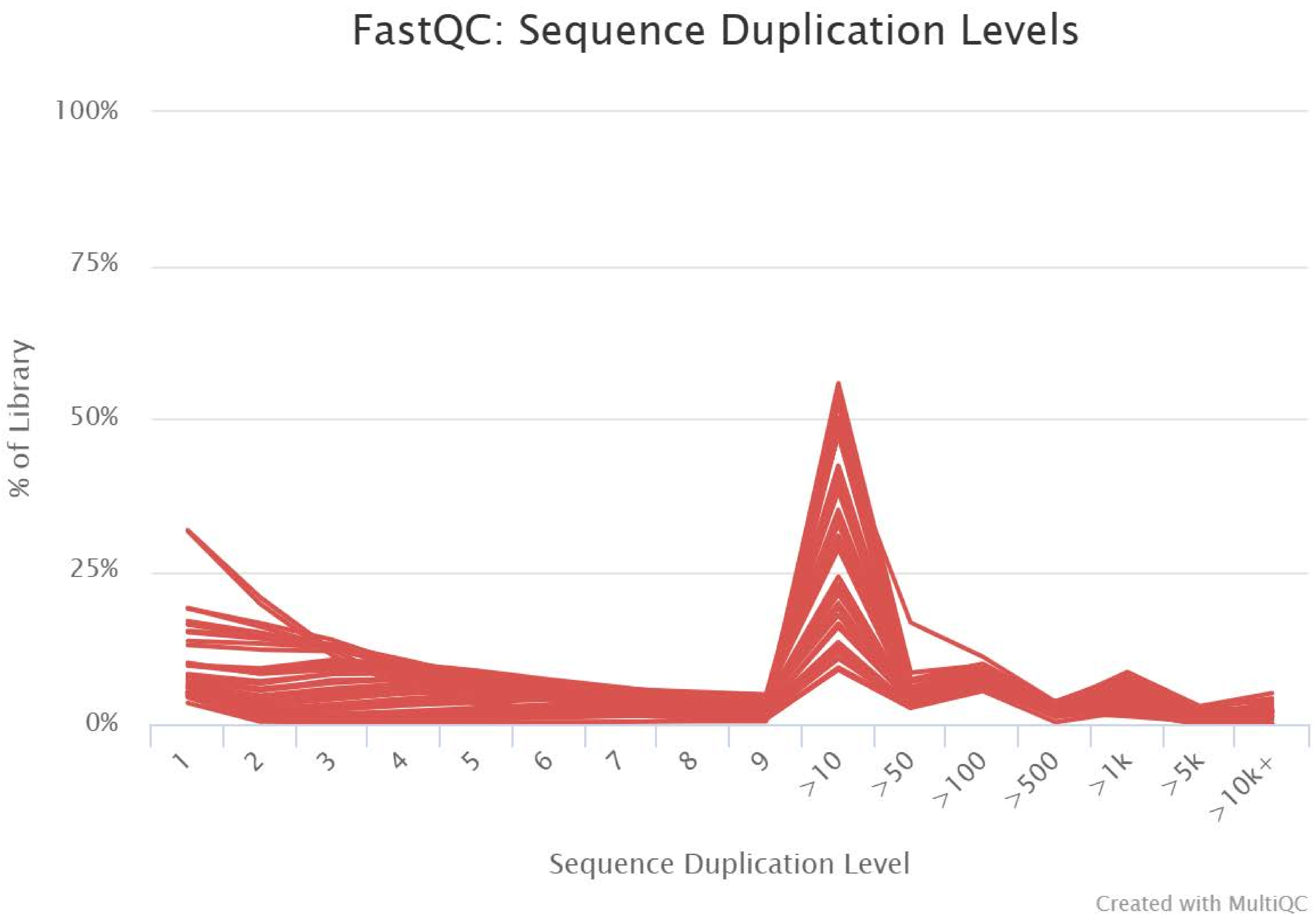
FastQC: Sequence Duplication Levels
100%
75%
50%
25%
0%
% of Library
1
2
3
4
5
6
7
8
9
>10
>50
>100
>500
>1k
>5k
>10k+
Sequence Duplication Level
Created with MultiQC

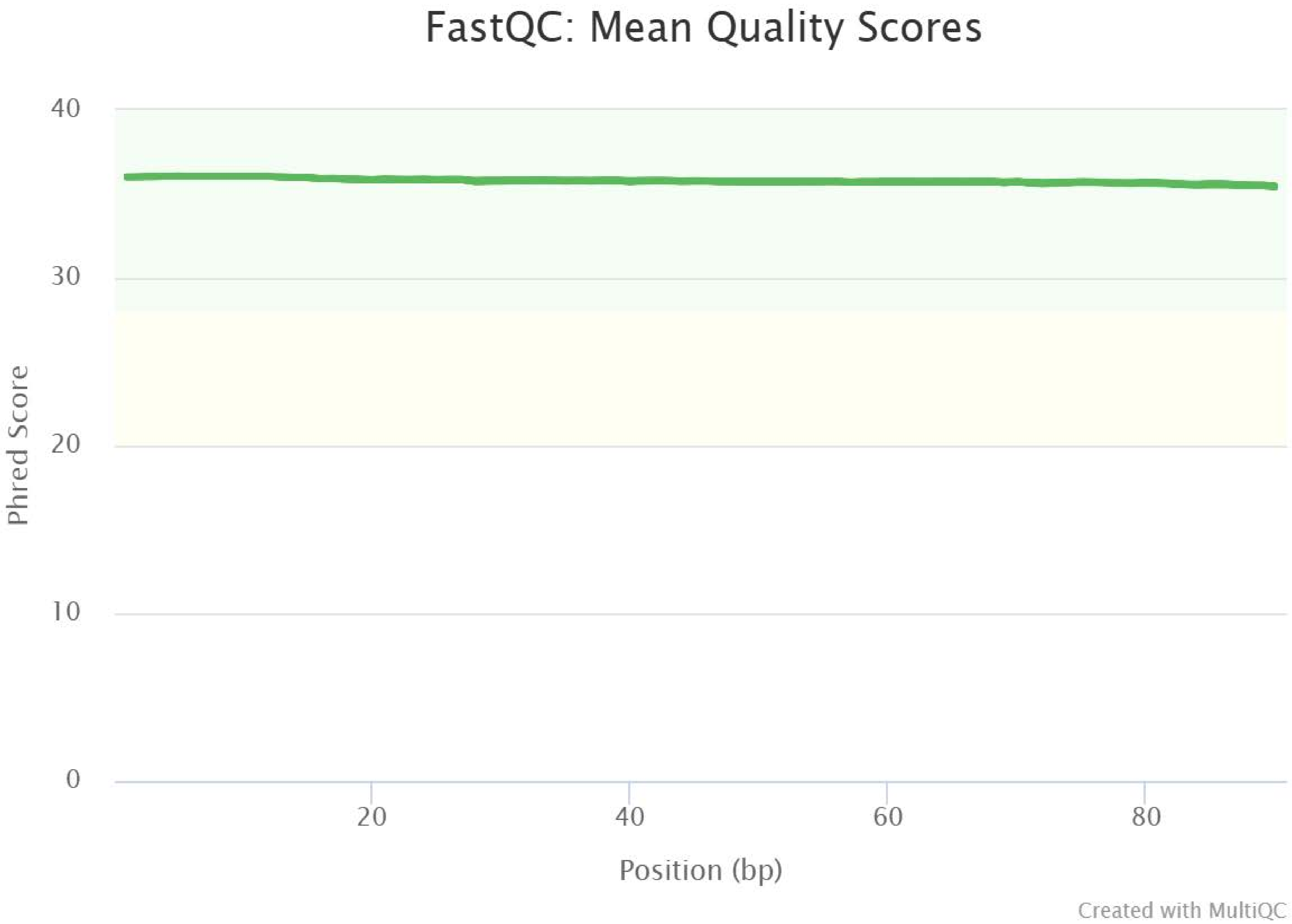

FastQC: Mean Quality Scores
40
30
20
10
0
Phred Score
20
40
60
80
Position (bp)
Created with MultiQC

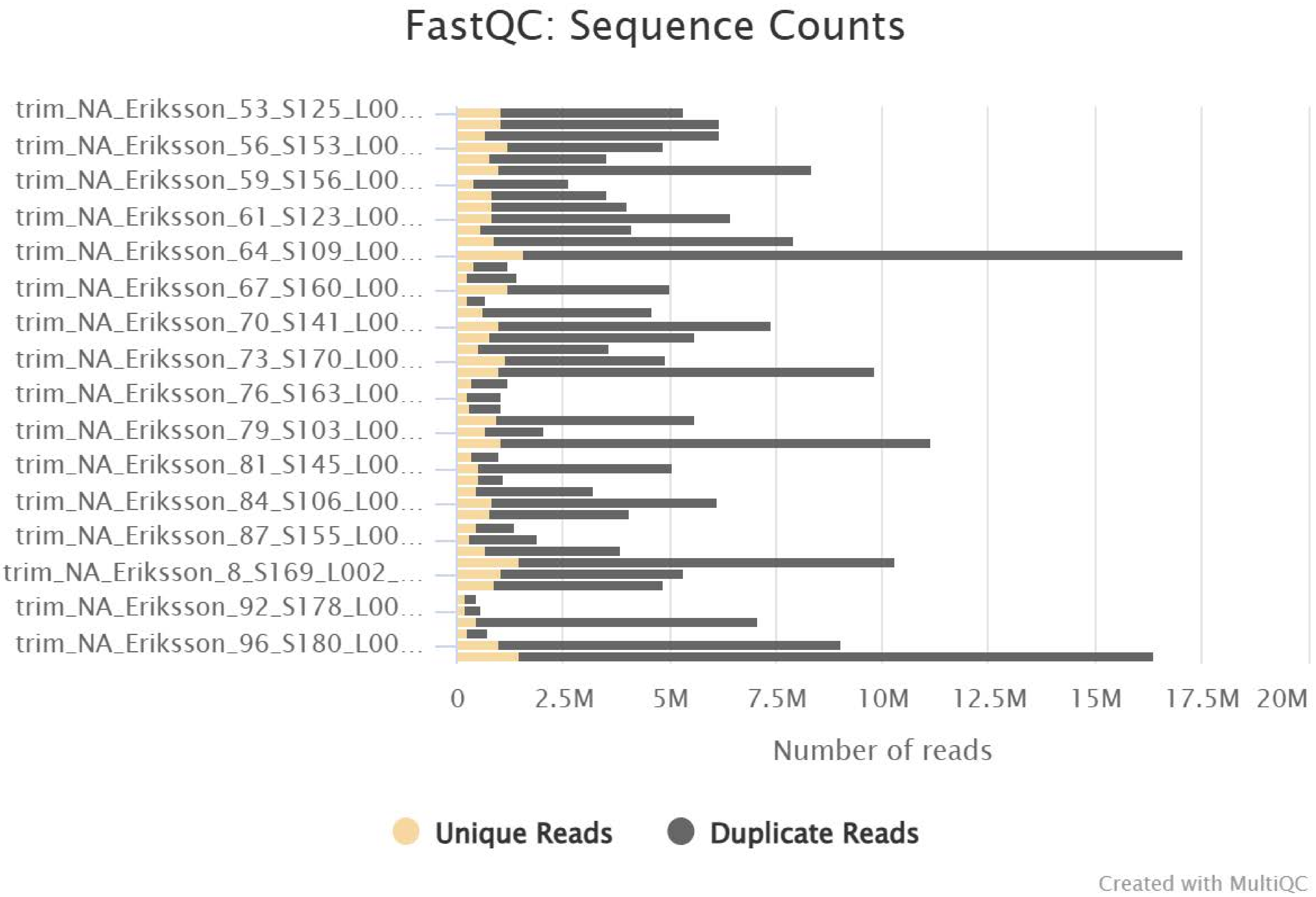
FastQC: Sequence Counts
trim_NA_Eriksson_53_S125_L00...
trim_NA_Eriksson_56_S153_L00...
trim_NA_Eriksson_59_S156_L00...
trim_NA_Eriksson_61_S123_L00...
trim_NA_Eriksson_64_S109_L00...
trim_NA_Eriksson_67_S160_L00...
trim_NA_Eriksson_70_S141_L00...
trim_NA_Eriksson_73_S170_L00...
trim_NA_Eriksson_76_S163_L00...
trim_NA_Eriksson_79_S103_L00...
trim_NA_Eriksson_81_S145_L00...
trim_NA_Eriksson_84_S106_L00...
trim_NA_Eriksson_87_S155_L00...
trim_NA_Eriksson_8_S169_L002_...
trim_NA_Eriksson_92_S178_L00...
trim_NA_Eriksson_96_S180_L00...
0
2.5M
5M
7.5M
10M
12.5M
15M
17.5M
20M
Number of reads
Unique Reads
Duplicate Reads
Created with MultiQC
