# Supplementary material for: Psilocybin elicits a conserved glucocorticoid-responsive gene signature across five 5-HT2A receptor-rich brain regions in rat
Source: Acta Neuropsychiatr. 2026 Apr 10;38:e37. doi: 10.1017/neu.2026.10075 (PMC13202413; doi:10.1017/neu.2026.10075)
Supplement: Veysi et al. supplementary material 8 — Veysi et al. supplementary material [file S0924270826100751sup008.pdf]

Supplement VIII

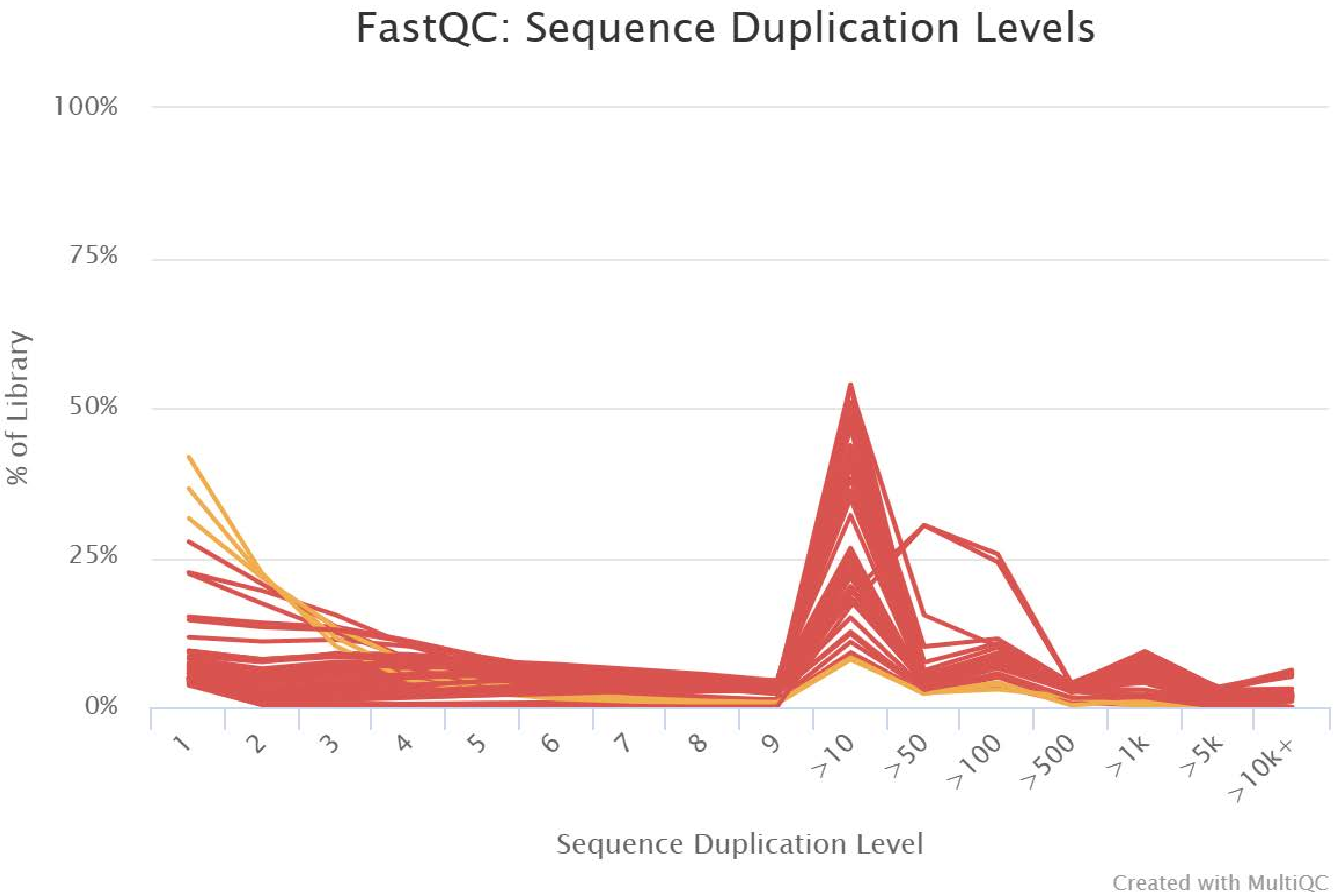

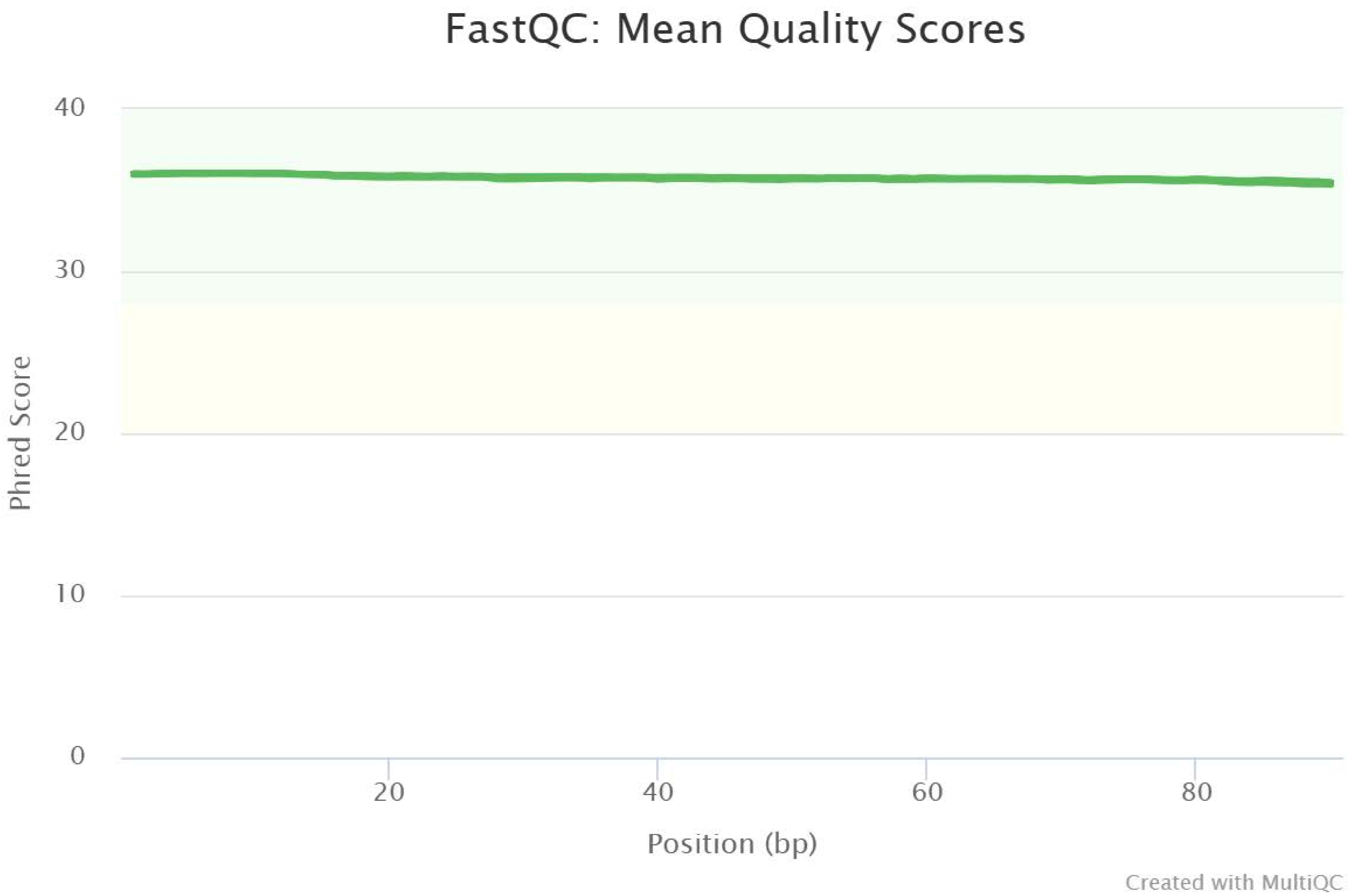
FastQC: Mean Quality Scores
Phred Score
40
30
20
10
0
20
40
60
80
Position (bp)
Created with MultiQC

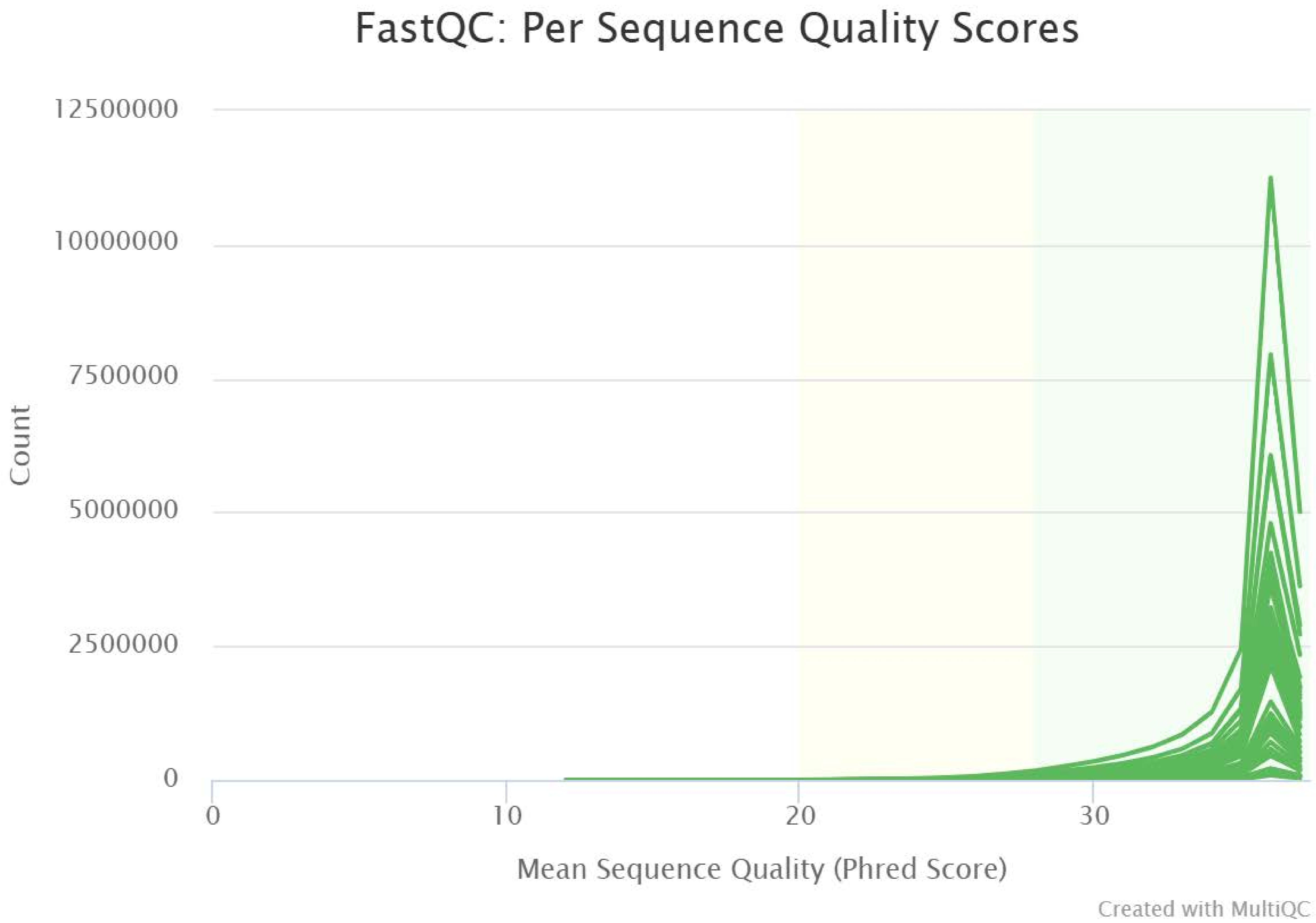
FastQC: Per Sequence Quality Scores
12500000
10000000
7500000
5000000
2500000
0
Count
0
10
20
30
Mean Sequence Quality (Phred Score)
Created with MultiQC

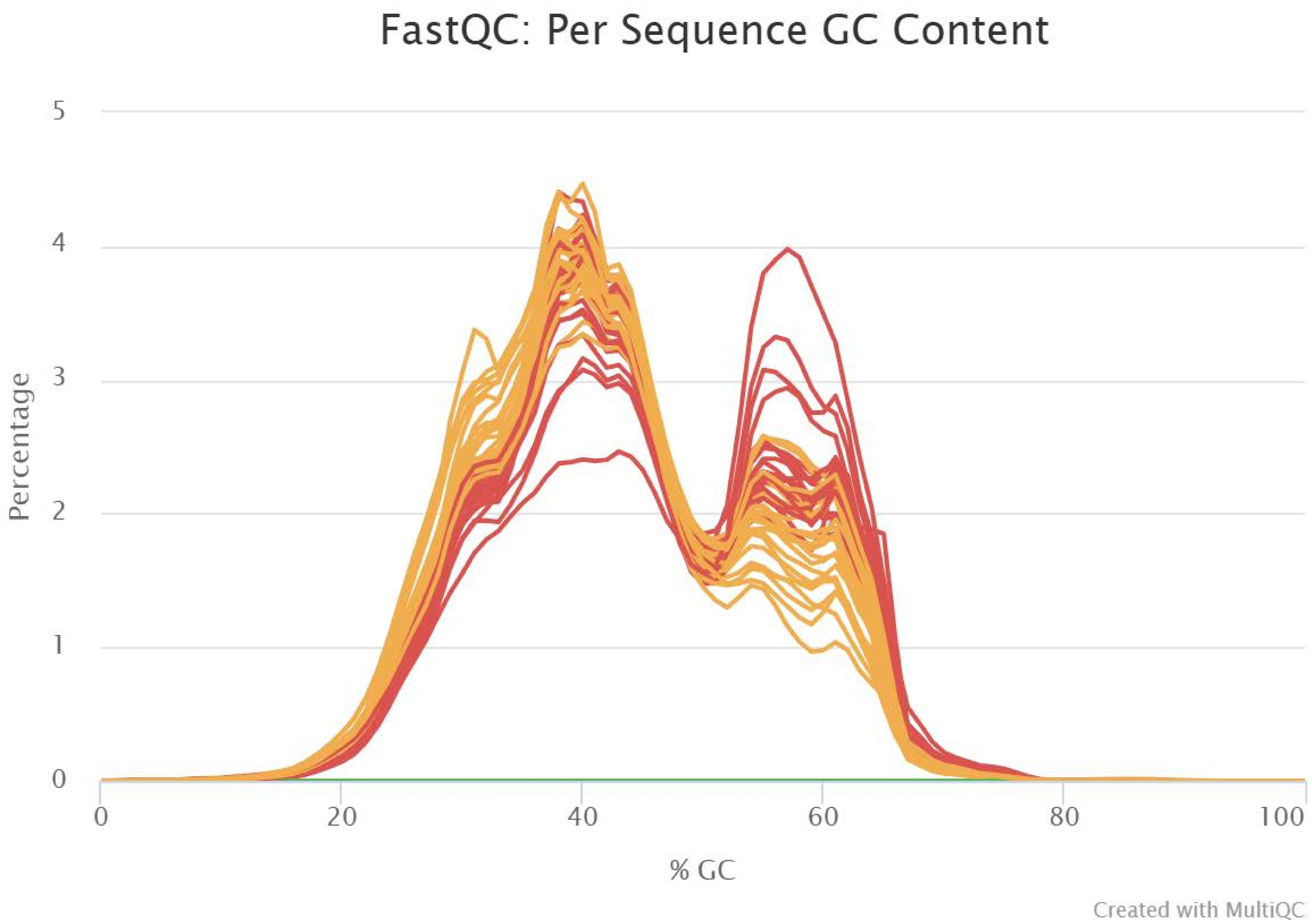
FastQC: Per Sequence GC Content
5
4
3
2
1
0
Percentage
0
20
40
60
80
100
% GC
Created with MultiQC

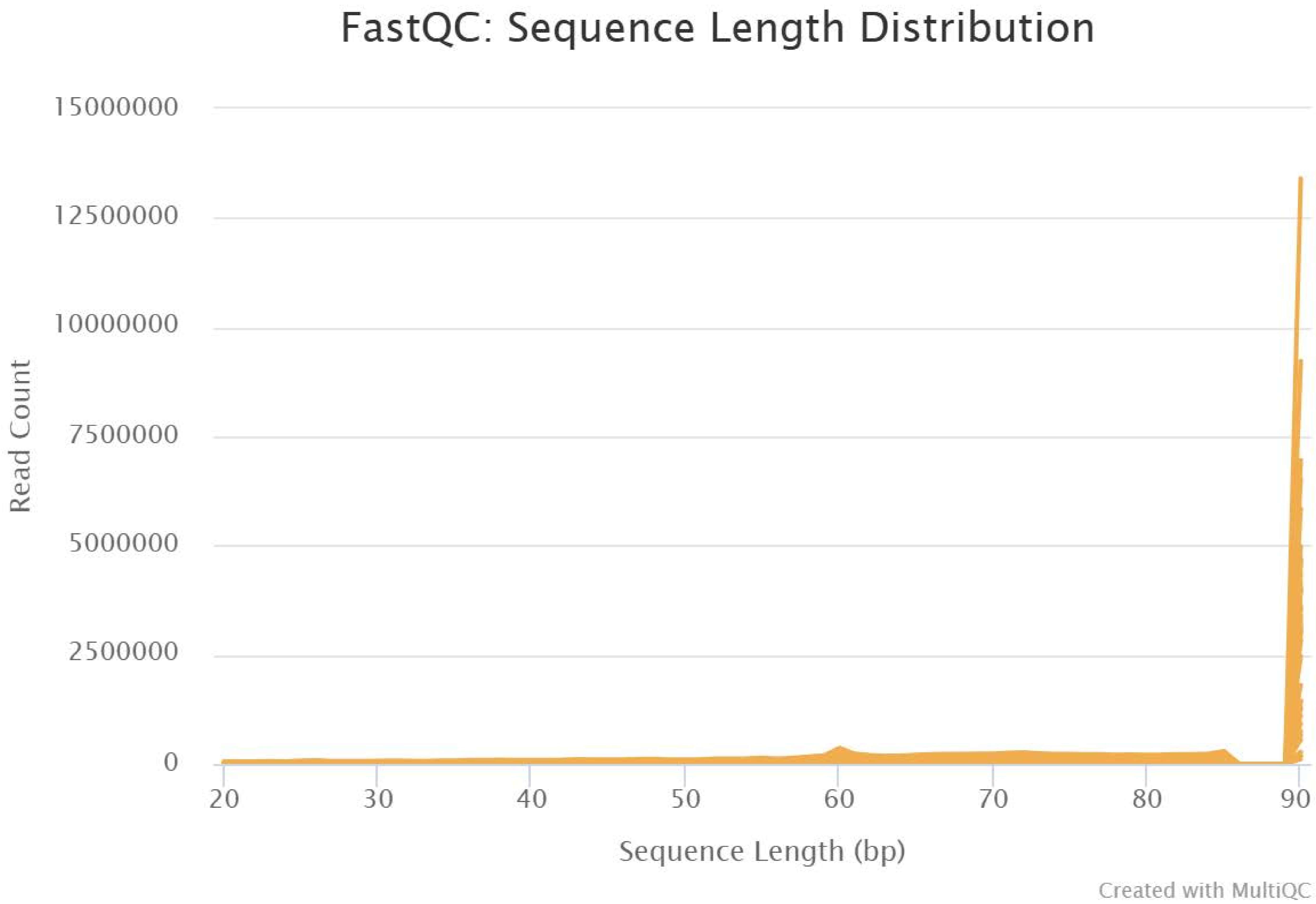
FastQC: Sequence Length Distribution
15000000
12500000
10000000
7500000
5000000
2500000
0
Read Count
20
30
40
50
60
70
80
90
Sequence Length (bp)
Created with MultiQC

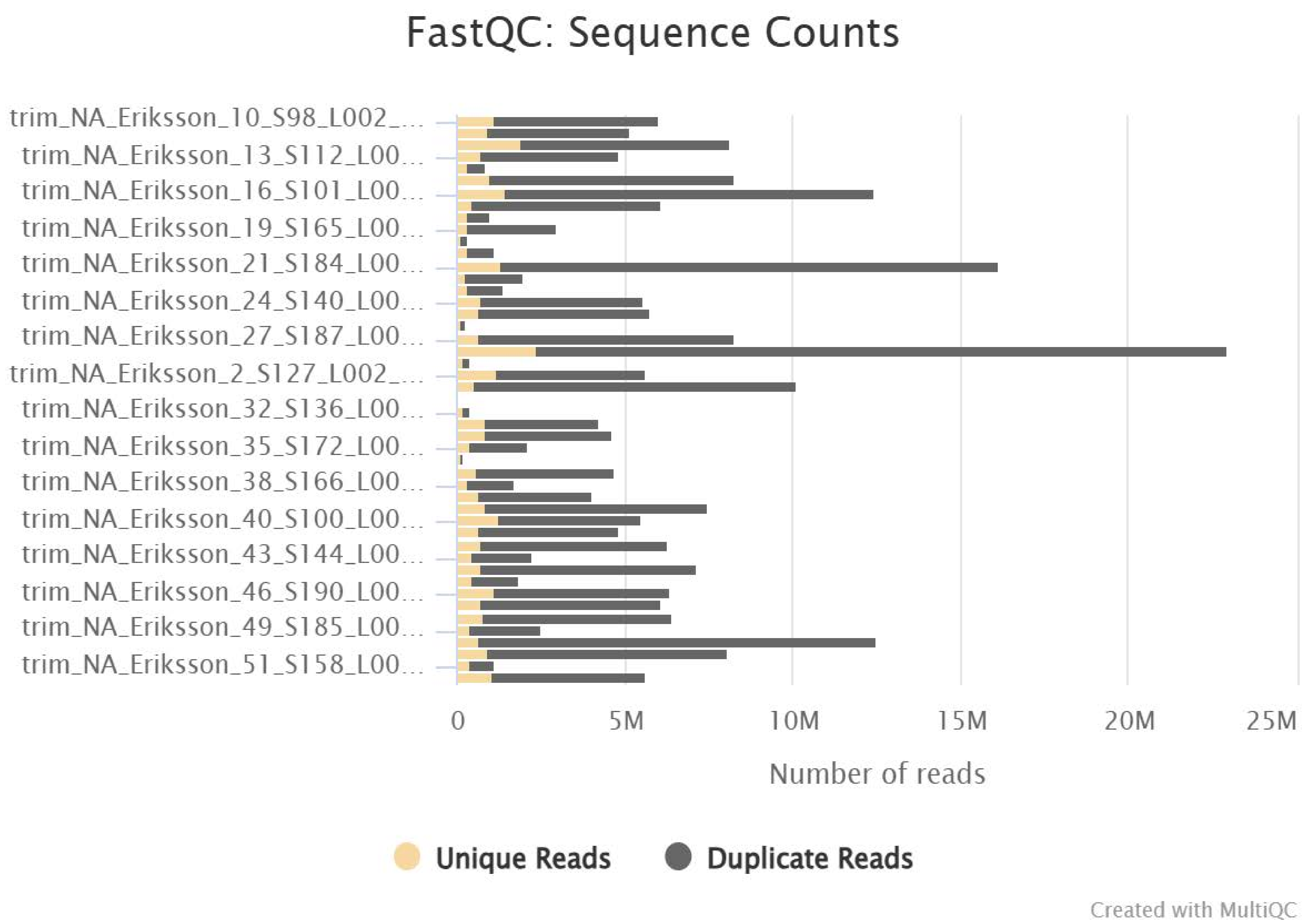
FastQC: Sequence Counts
trim_NA_Eriksson_10_S98_L002_...
trim_NA_Eriksson_13_S112_L00...
trim_NA_Eriksson_16_S101_L00...
trim_NA_Eriksson_19_S165_L00...
trim_NA_Eriksson_21_S184_L00...
trim_NA_Eriksson_24_S140_L00...
trim_NA_Eriksson_27_S187_L00...
trim_NA_Eriksson_2_S127_L002_...
trim_NA_Eriksson_32_S136_L00...
trim_NA_Eriksson_35_S172_L00...
trim_NA_Eriksson_38_S166_L00...
trim_NA_Eriksson_40_S100_L00...
trim_NA_Eriksson_43_S144_L00...
trim_NA_Eriksson_46_S190_L00...
trim_NA_Eriksson_49_S185_L00...
trim_NA_Eriksson_51_S158_L00...
0
5M
10M
15M
20M
25M
Number of reads
Unique Reads
Duplicate Reads
Created with MultiQC

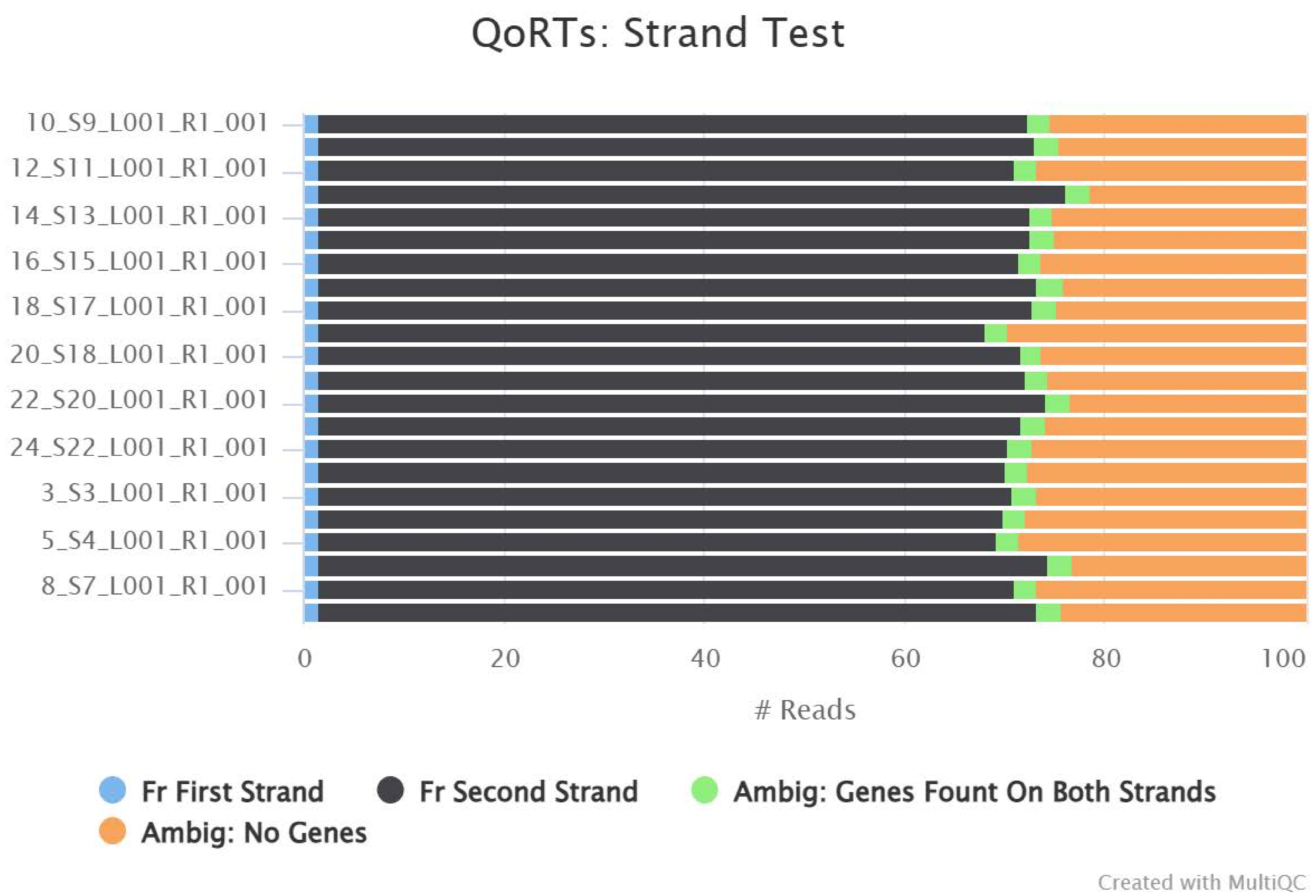

QoRTs: Strand Test
10_S9_L001_R1_001
12_S11_L001_R1_001
14_S13_L001_R1_001
16_S15_L001_R1_001
18_S17_L001_R1_001
20_S18_L001_R1_001
22_S20_L001_R1_001
24_S22_L001_R1_001
3_S3_L001_R1_001
5_S4_L001_R1_001
8_S7_L001_R1_001
0
20
40
60
80
100
Reads
Fr First Strand
Fr Second Strand
Ambig: Genes Fount On Both Strands
Ambig: No Genes
Created with MultiQC

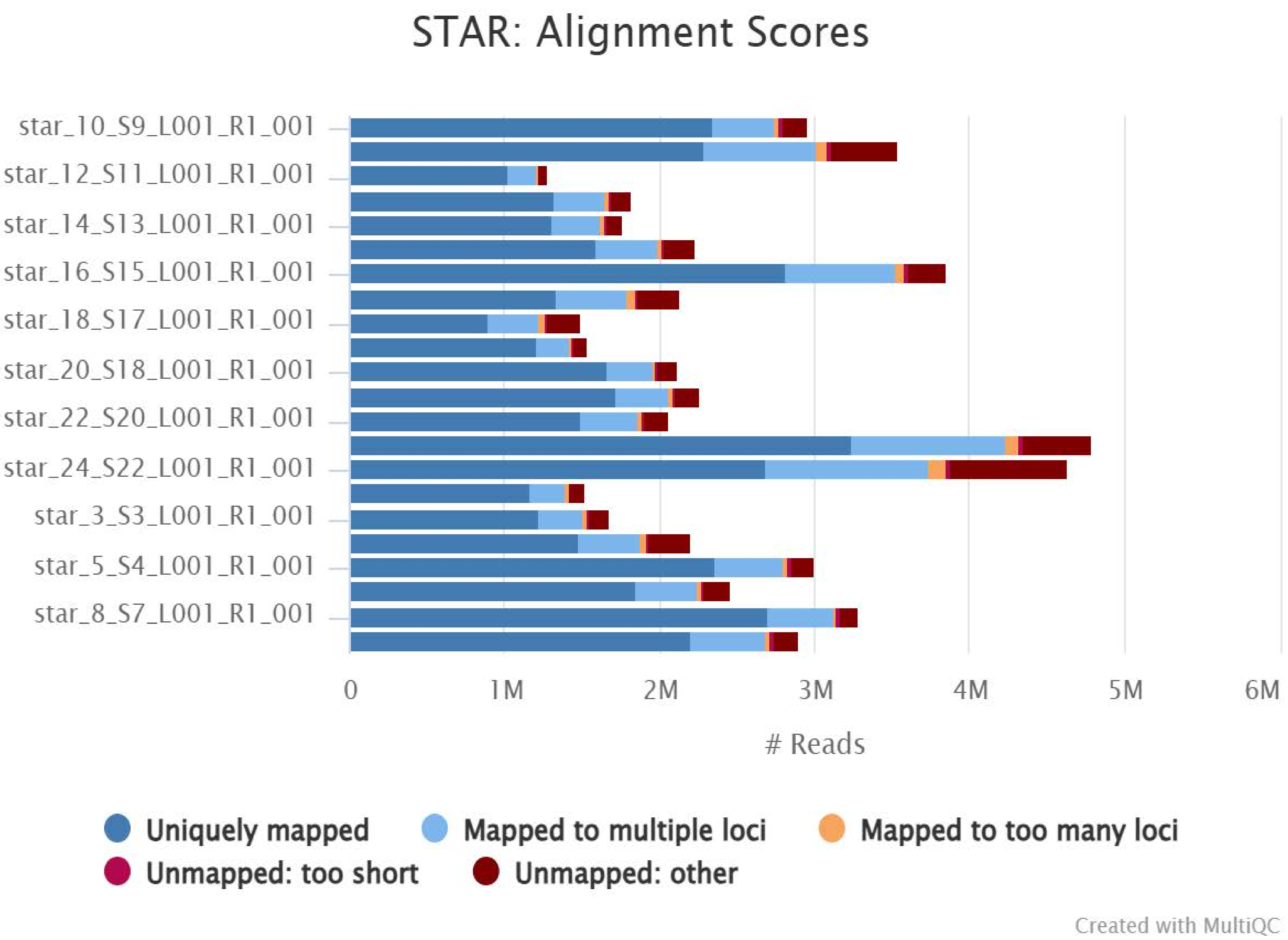
STAR: Alignment Scores
star_10_S9_L001_R1_001
star_12_S11_L001_R1_001
star_14_S13_L001_R1_001
star_16_S15_L001_R1_001
star_18_S17_L001_R1_001
star_20_S18_L001_R1_001
star_22_S20_L001_R1_001
star_24_S22_L001_R1_001
star_3_S3_L001_R1_001
star_5_S4_L001_R1_001
star_8_S7_L001_R1_001
0
1M
2M
3M
4M
5M
6M
Reads
Uniquely mapped
Mapped to multiple loci
Mapped to too many loci
Unmapped: too short
Unmapped: other
Created with MultiQC

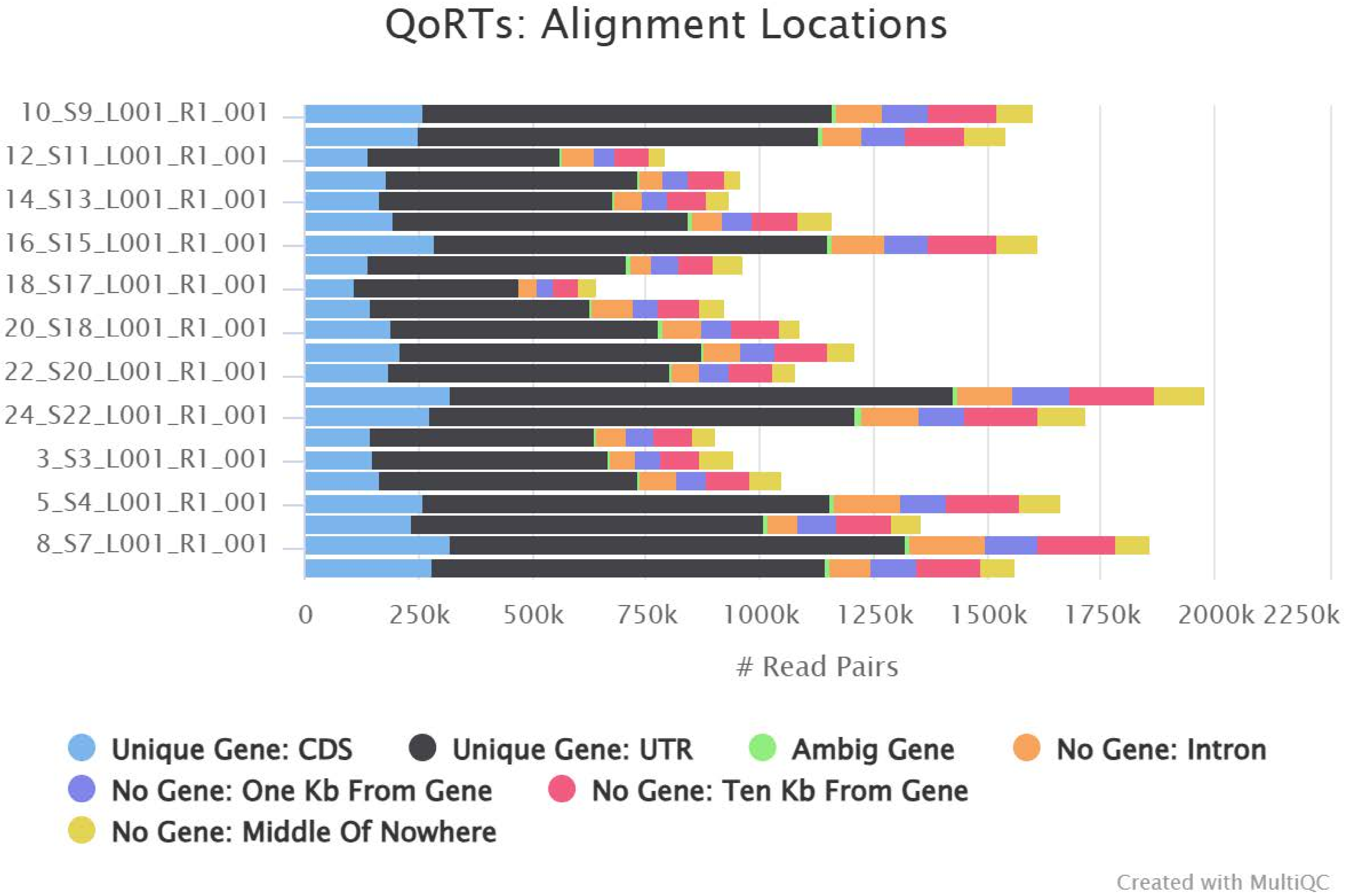

QoRTs: Alignment Locations
10_S9_L001_R1_001
12_S11_L001_R1_001
14_S13_L001_R1_001
16_S15_L001_R1_001
18_S17_L001_R1_001
20_S18_L001_R1_001
22_S20_L001_R1_001
24_S22_L001_R1_001
3_S3_L001_R1_001
5_S4_L001_R1_001
8_S7_L001_R1_001
0
250k
500k
750k
1000k
1250k
1500k
1750k
2000k
2250k
Read Pairs
Unique Gene: CDS
Unique Gene: UTR
Ambig Gene
No Gene: Intron
No Gene: One Kb From Gene
No Gene: Ten Kb From Gene
No Gene: Middle Of Nowhere
Created with MultiQC

FastQC: Status Checks

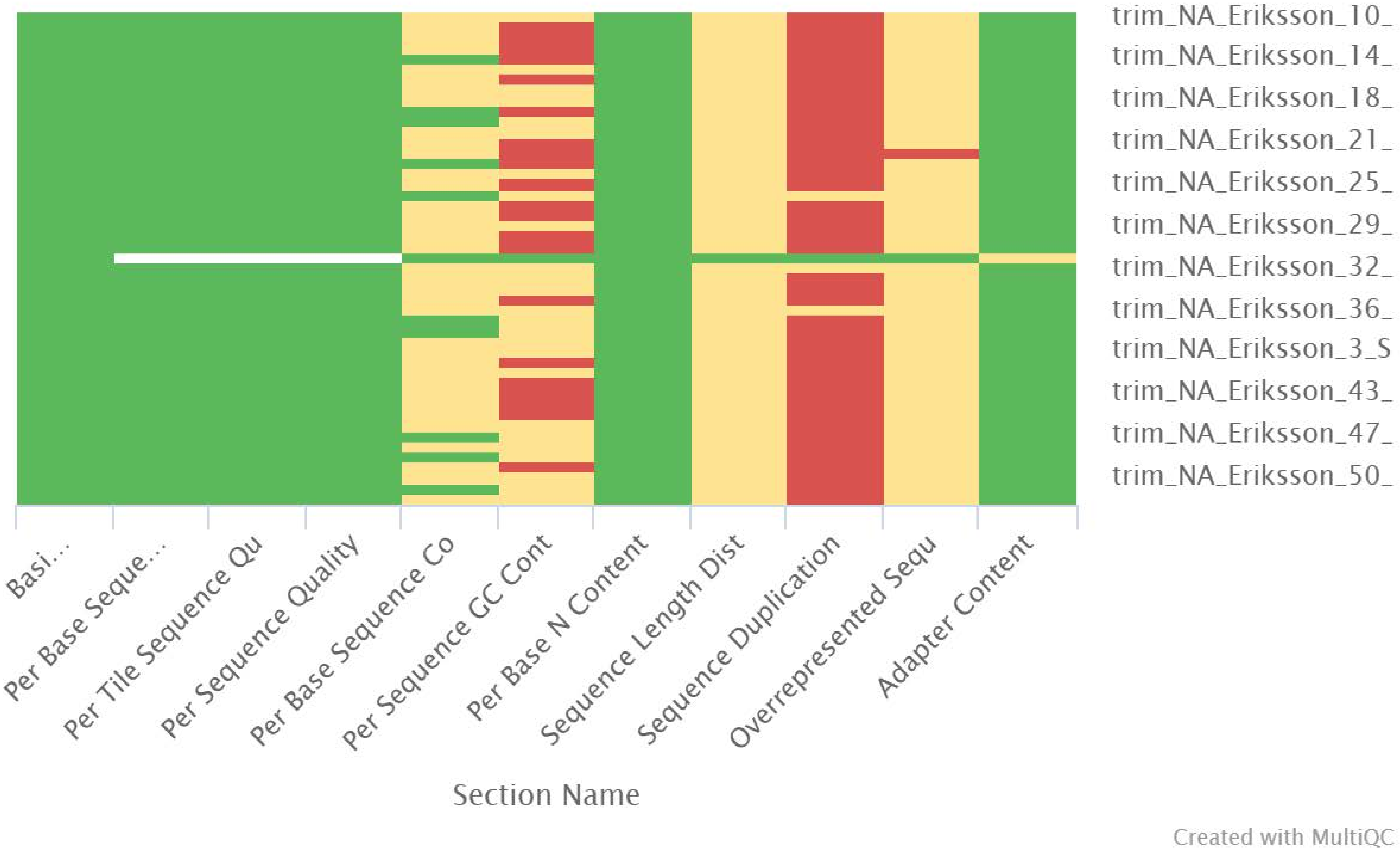

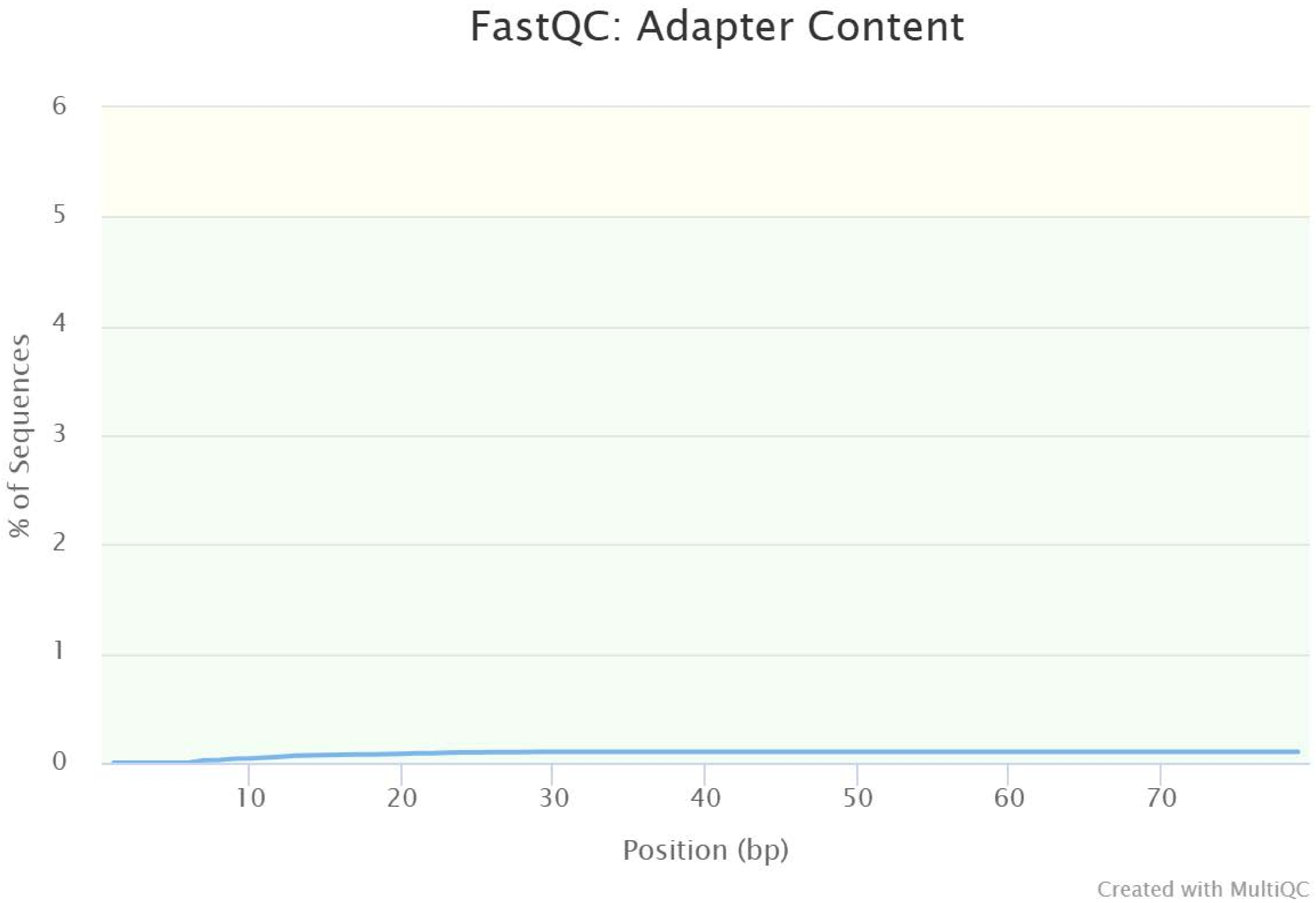
FastQC: Adapter Content
6
5
4
3
2
1
0
% of Sequences
10
20
30
40
50
60
70
Position (bp)
Created with MultiQC

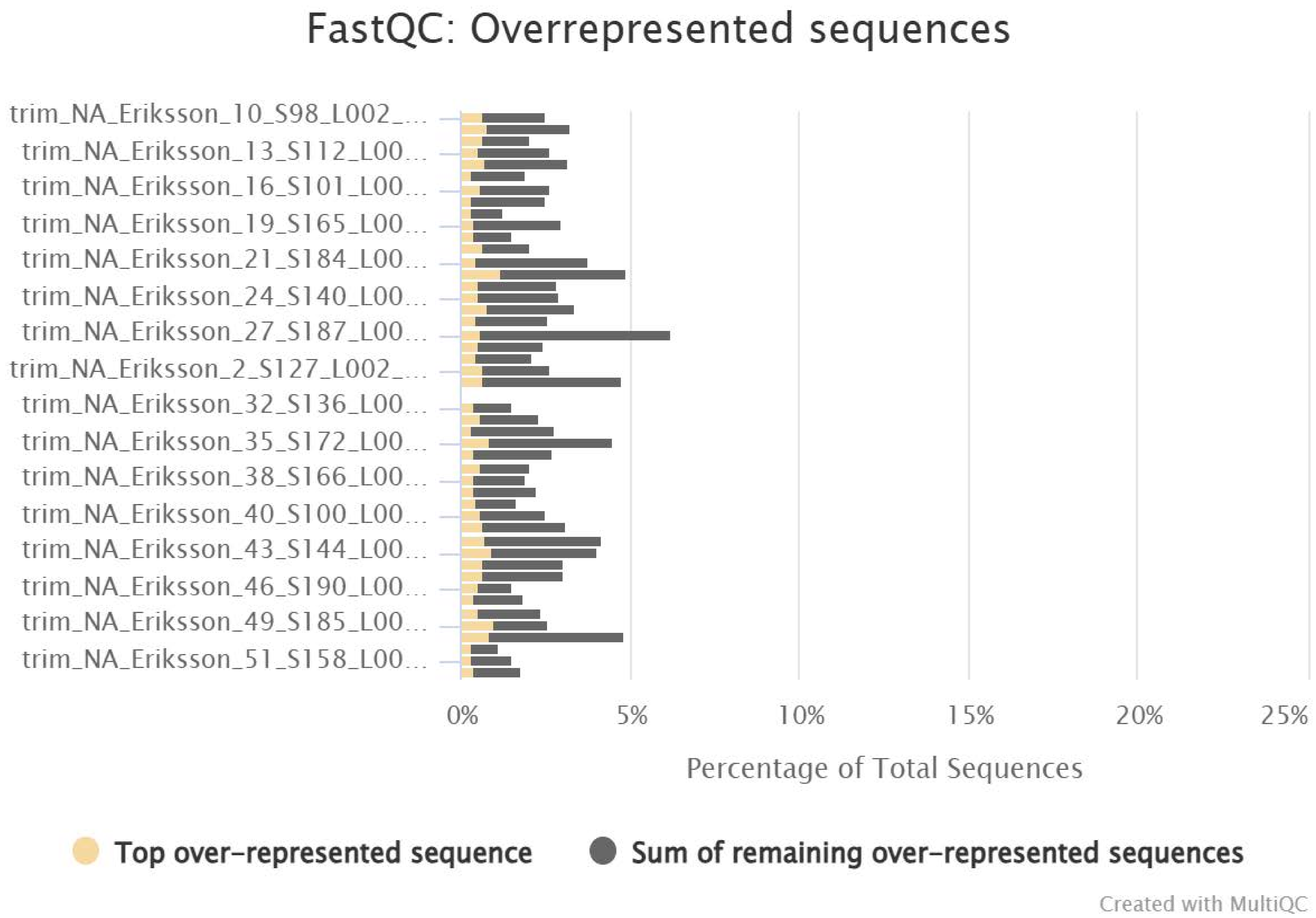
FastQC: Overrepresented sequences
trim_NA_Eriksson_10_S98_L002_...
trim_NA_Eriksson_13_S112_L00...
trim_NA_Eriksson_16_S101_L00...
trim_NA_Eriksson_19_S165_L00...
trim_NA_Eriksson_21_S184_L00...
trim_NA_Eriksson_24_S140_L00...
trim_NA_Eriksson_27_S187_L00...
trim_NA_Eriksson_2_S127_L002_...
trim_NA_Eriksson_32_S136_L00...
trim_NA_Eriksson_35_S172_L00...
trim_NA_Eriksson_38_S166_L00...
trim_NA_Eriksson_40_S100_L00...
trim_NA_Eriksson_43_S144_L00...
trim_NA_Eriksson_46_S190_L00...
trim_NA_Eriksson_49_S185_L00...
trim_NA_Eriksson_51_S158_L00...
0%
5%
10%
15%
20%
25%
Percentage of Total Sequences
Top over-represented sequence
Sum of remaining over-represented sequences
Created with MultiQC

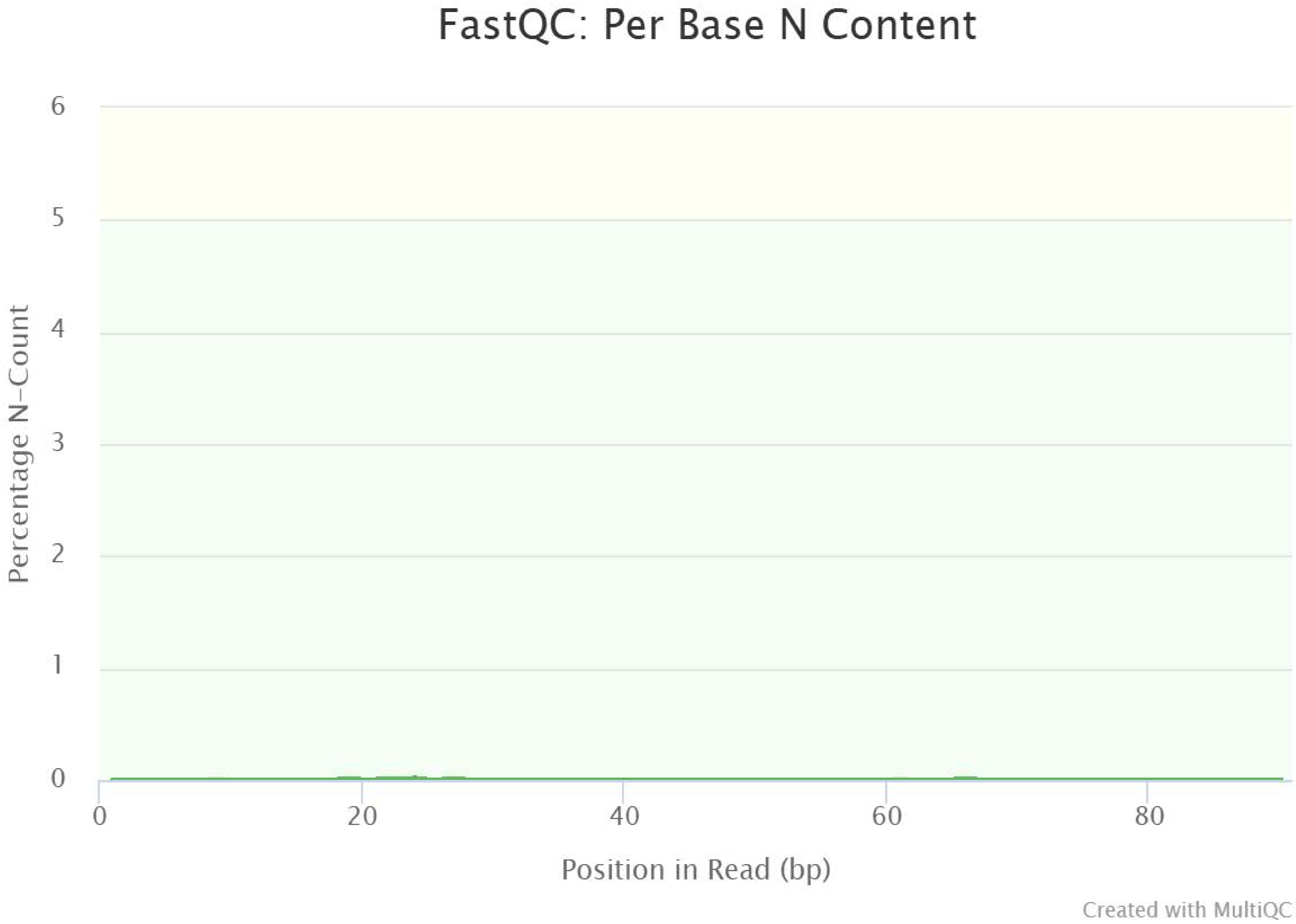
FastQC: Per Base N Content
Percentage N-Count
6
5
4
3
2
1
0
0
20
40
60
80
Position in Read (bp)
Created with MultiQC
