# Supplementary material for: Psilocybin elicits a conserved glucocorticoid-responsive gene signature across five 5-HT2A receptor-rich brain regions in rat
Source: Acta Neuropsychiatr. 2026 Apr 10;38:e37. doi: 10.1017/neu.2026.10075 (PMC13202413; doi:10.1017/neu.2026.10075)
Supplement: Veysi et al. supplementary material 9 — Veysi et al. supplementary material [file S0924270826100751sup009.pdf]

Supplement IX

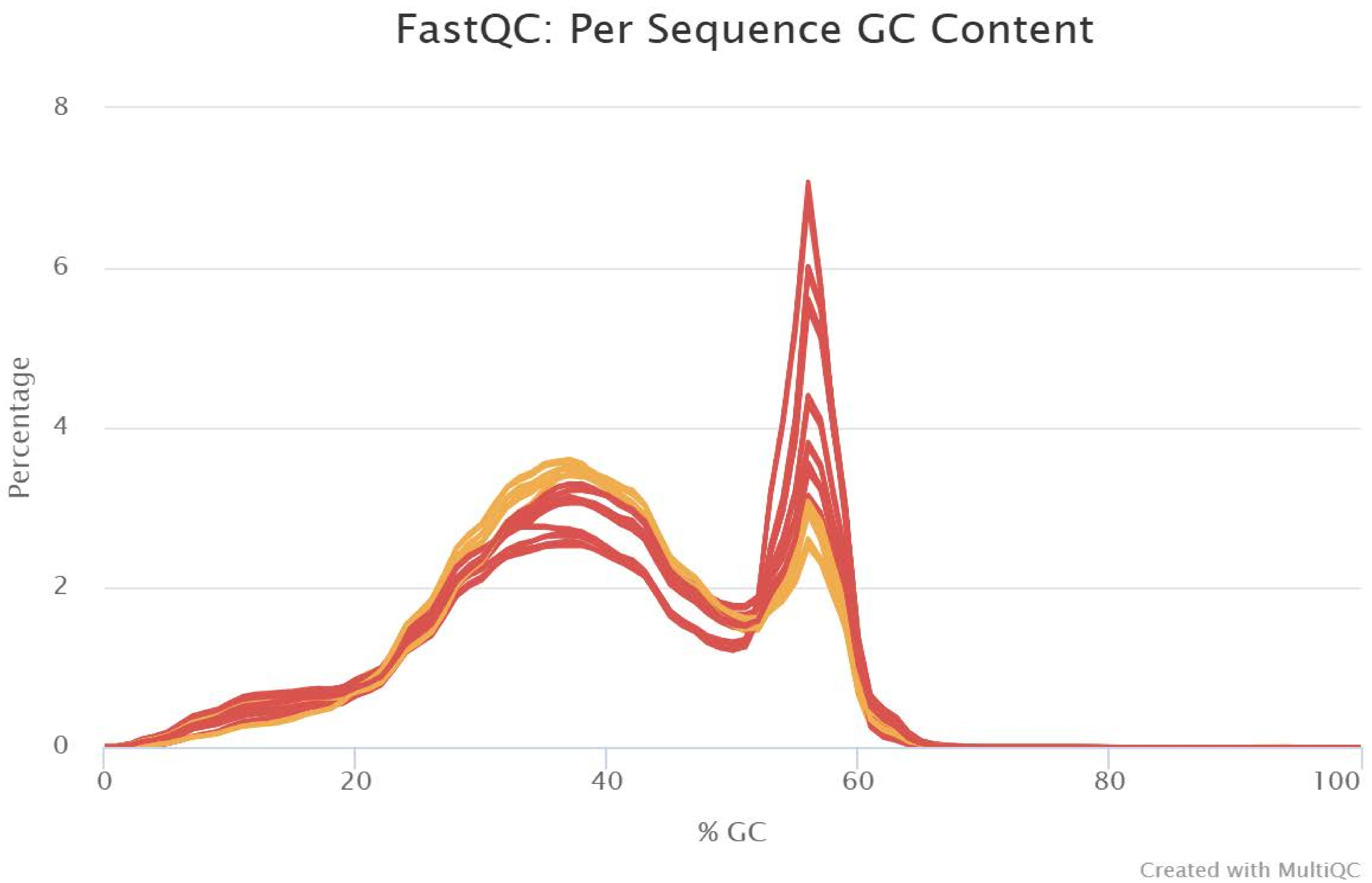

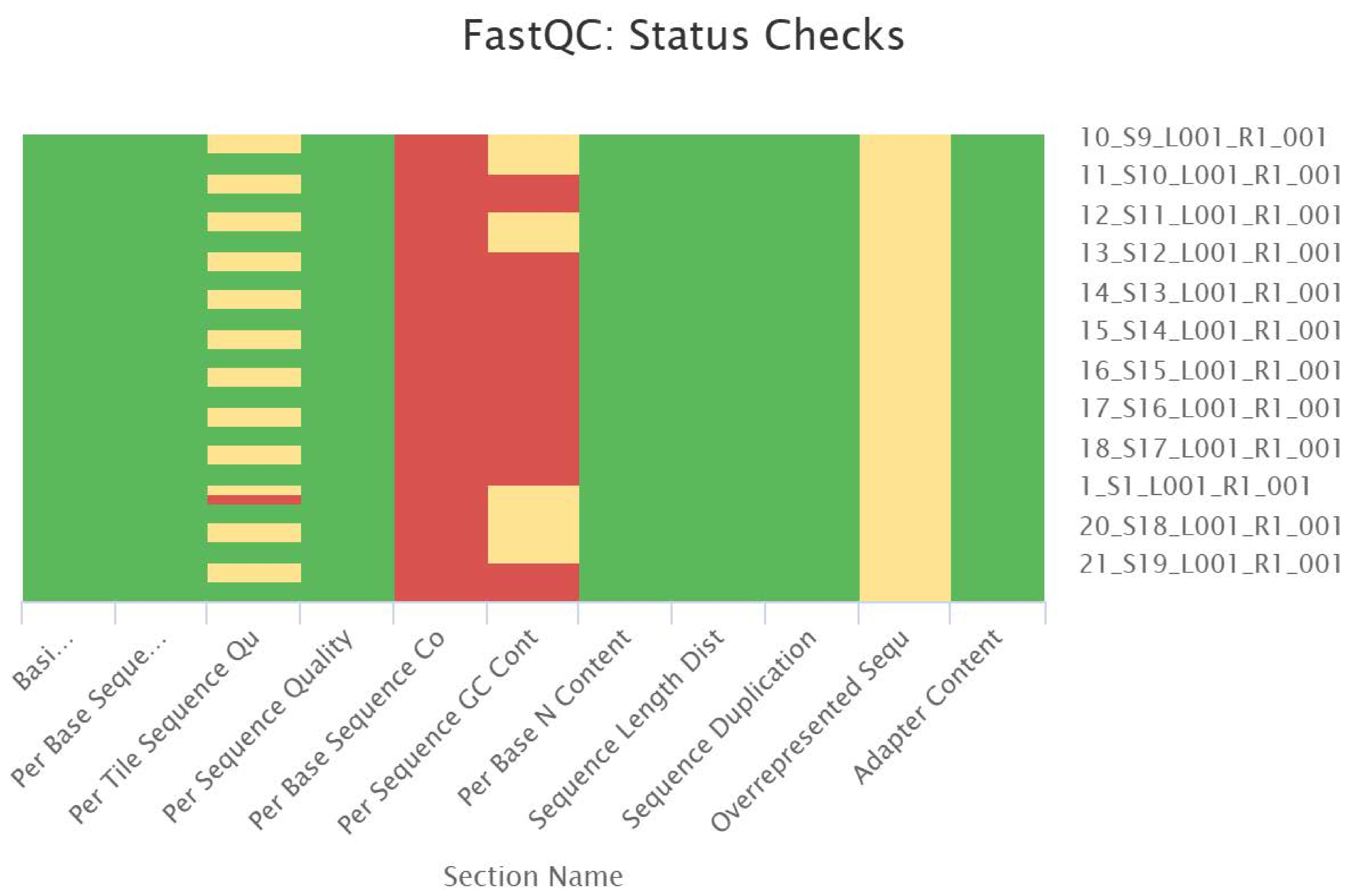

Created with MultiQC

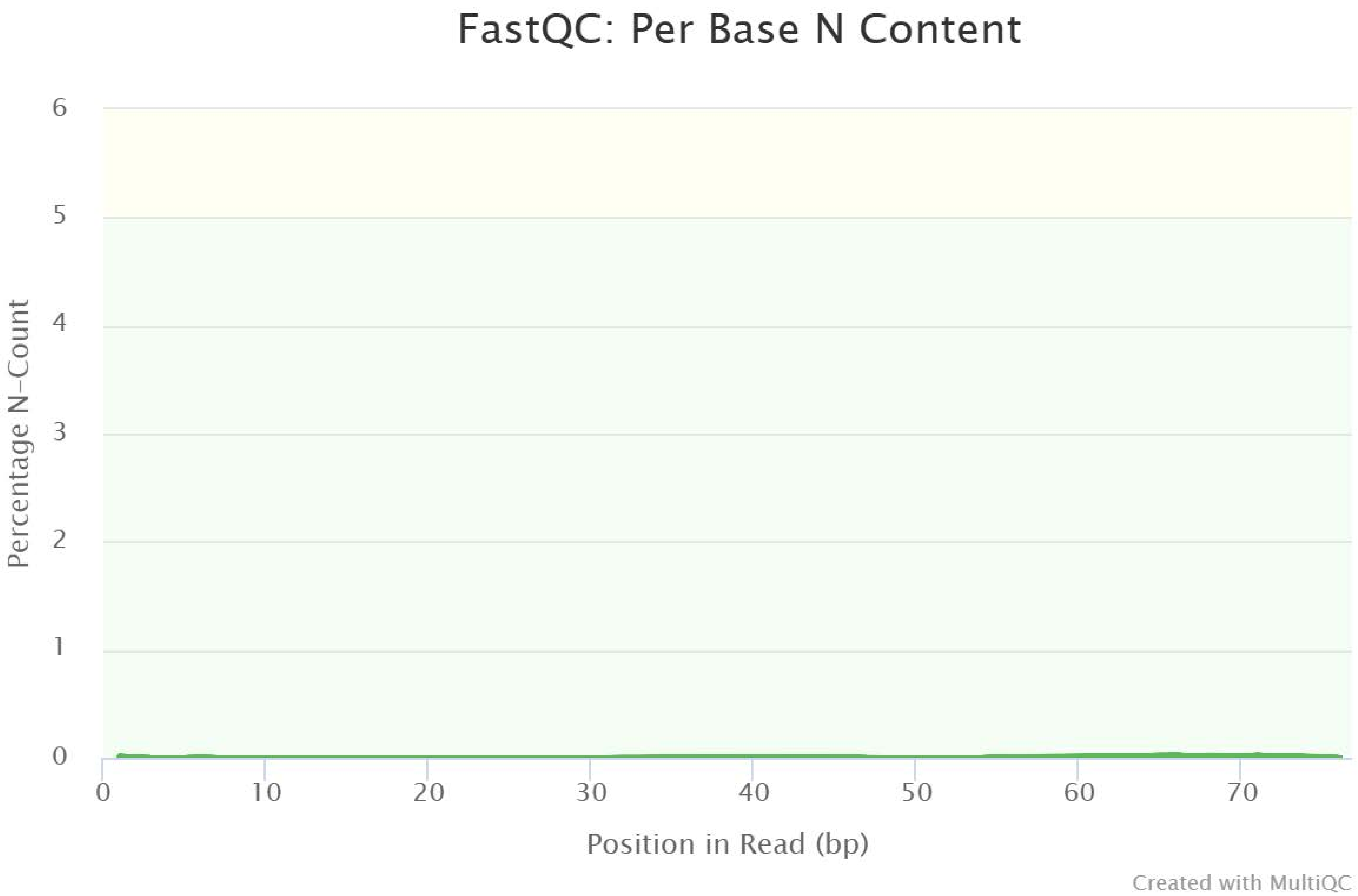
FastQC: Per Base N Content
Percentage N-Count
0
1
2
3
4
5
6
0
10
20
30
40
50
60
70
Position in Read (bp)
Created with MultiQC

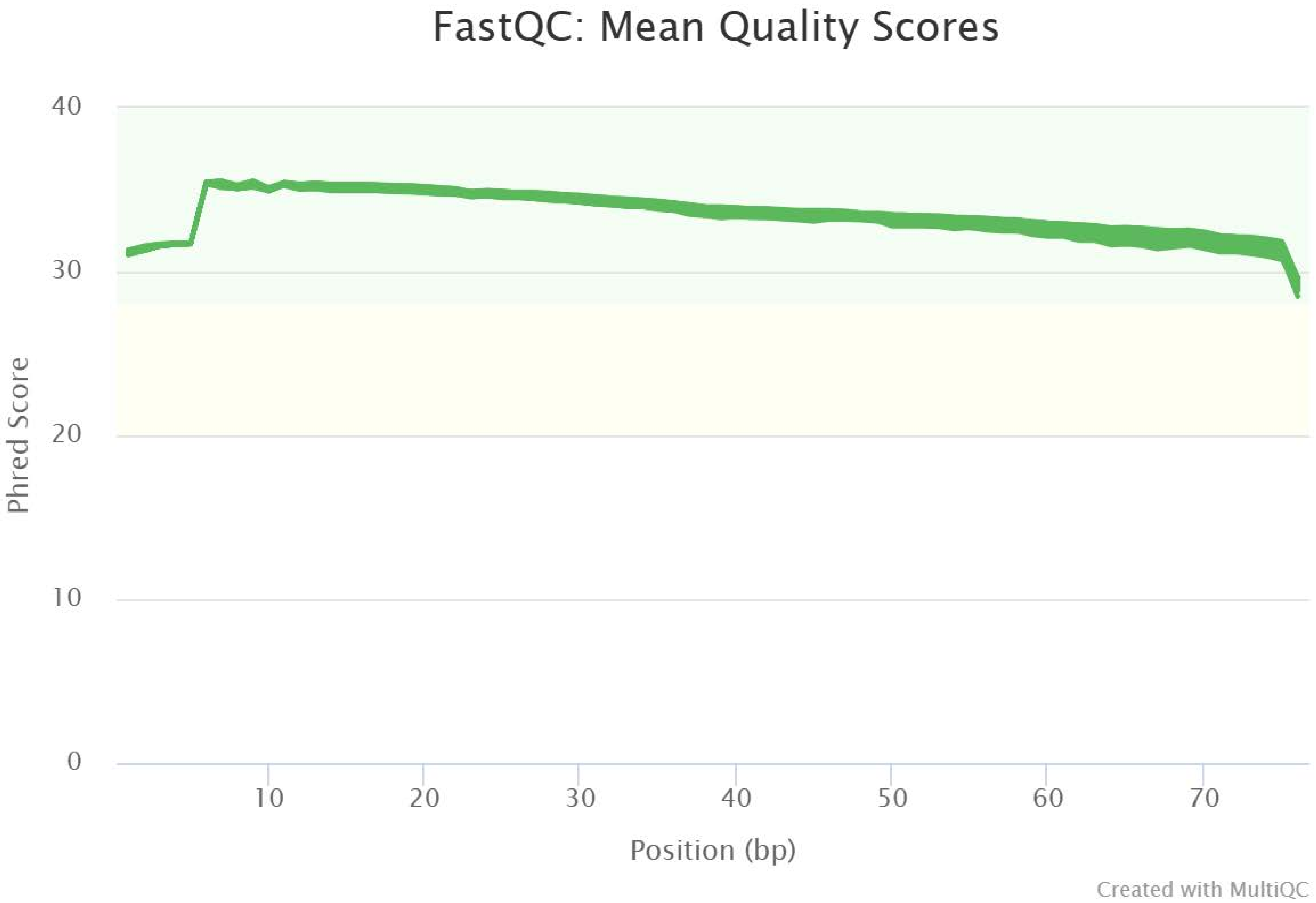
FastQC: Mean Quality Scores
Phred Score
40
30
20
10
0
10
20
30
40
50
60
70
Position (bp)
Created with MultiQC

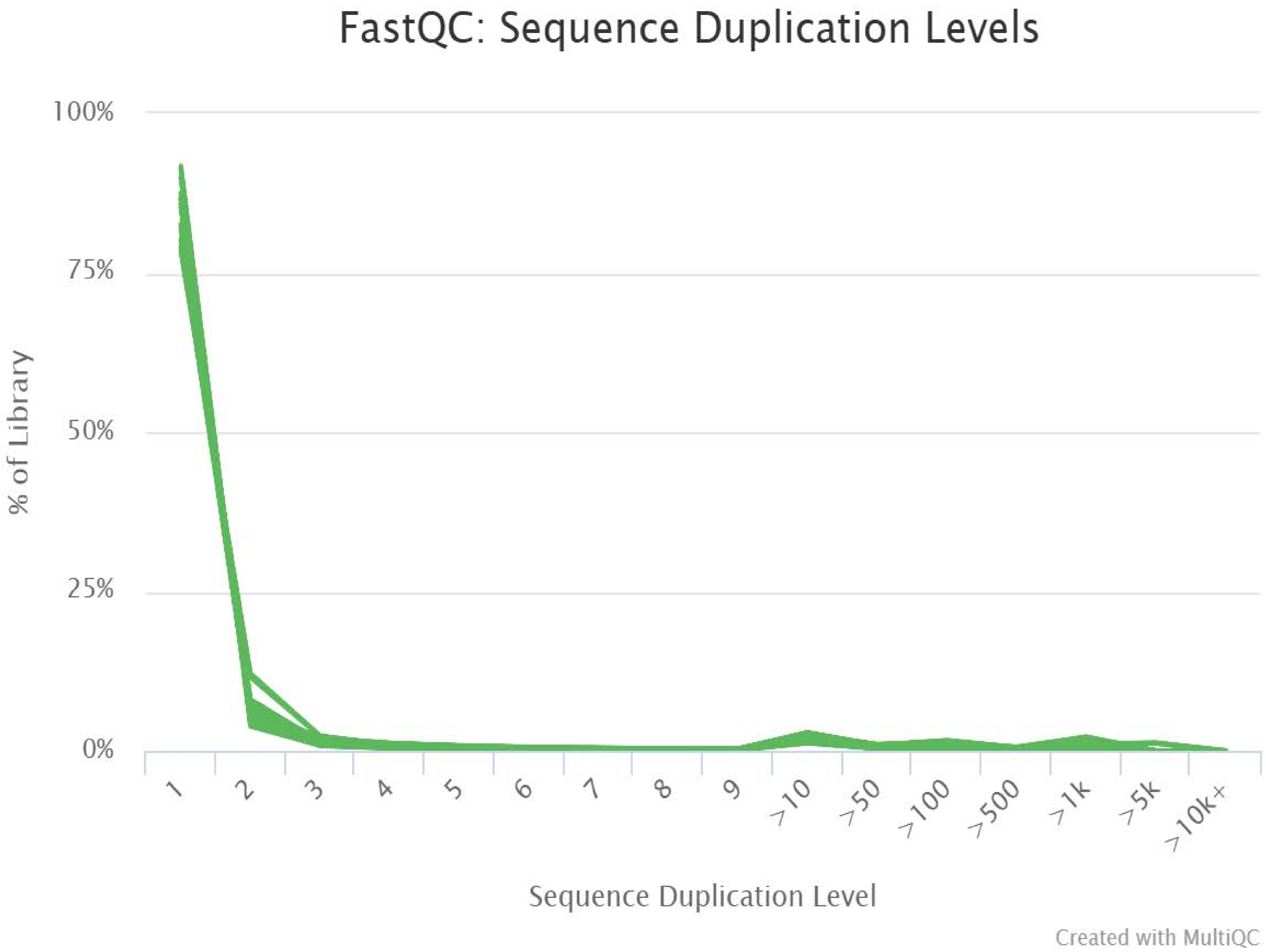
FastQC: Sequence Duplication Levels
100%
75%
50%
25%
0%
% of Library
1
2
3
4
5
6
7
8
9
>10
>50
>100
>500
>1k
>5k
>10k+
Sequence Duplication Level
Created with MultiQC

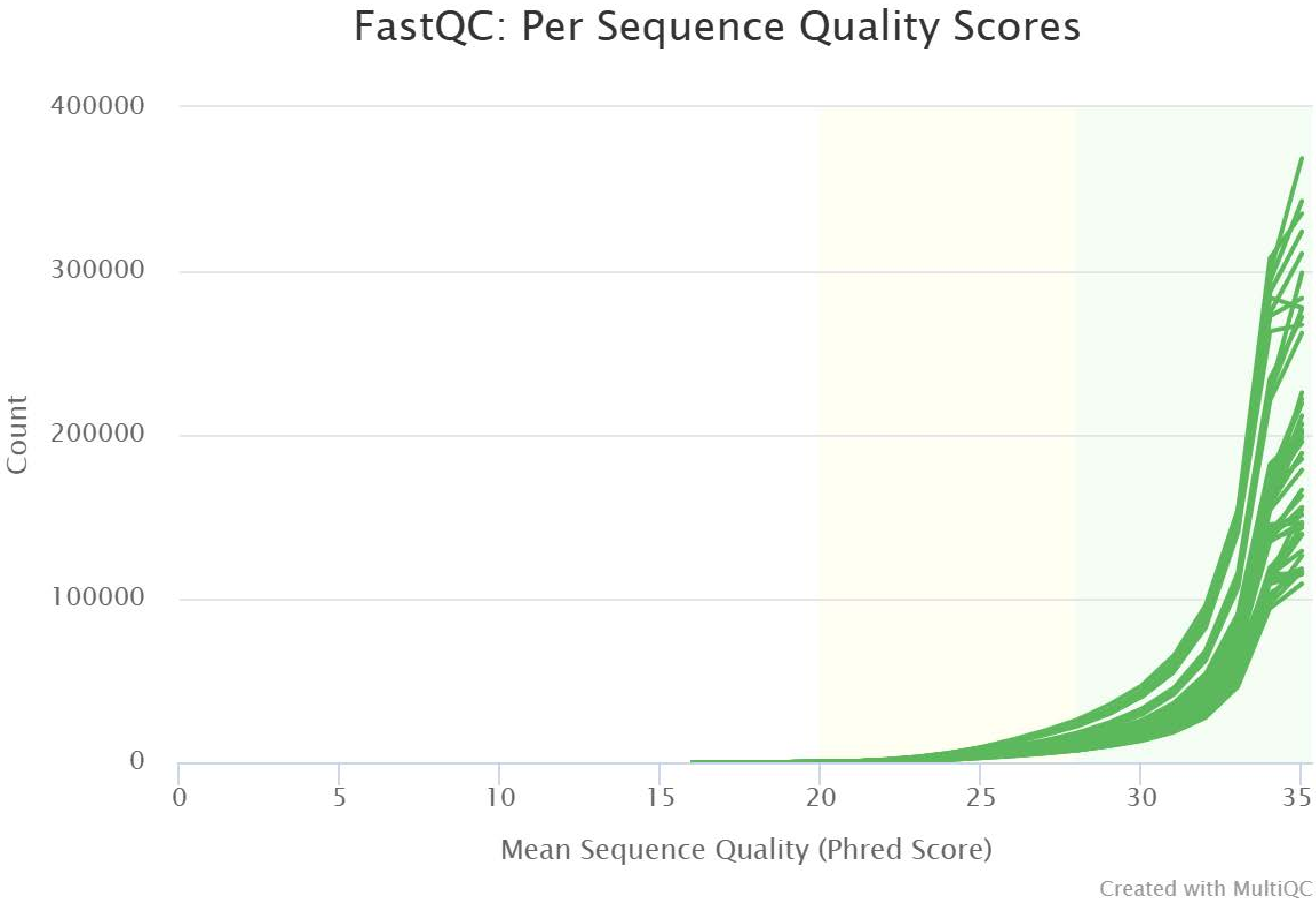
FastQC: Per Sequence Quality Scores
400000
300000
200000
100000
0
Count
0
5
10
15
20
25
30
35
Mean Sequence Quality (Phred Score)
Created with MultiQC

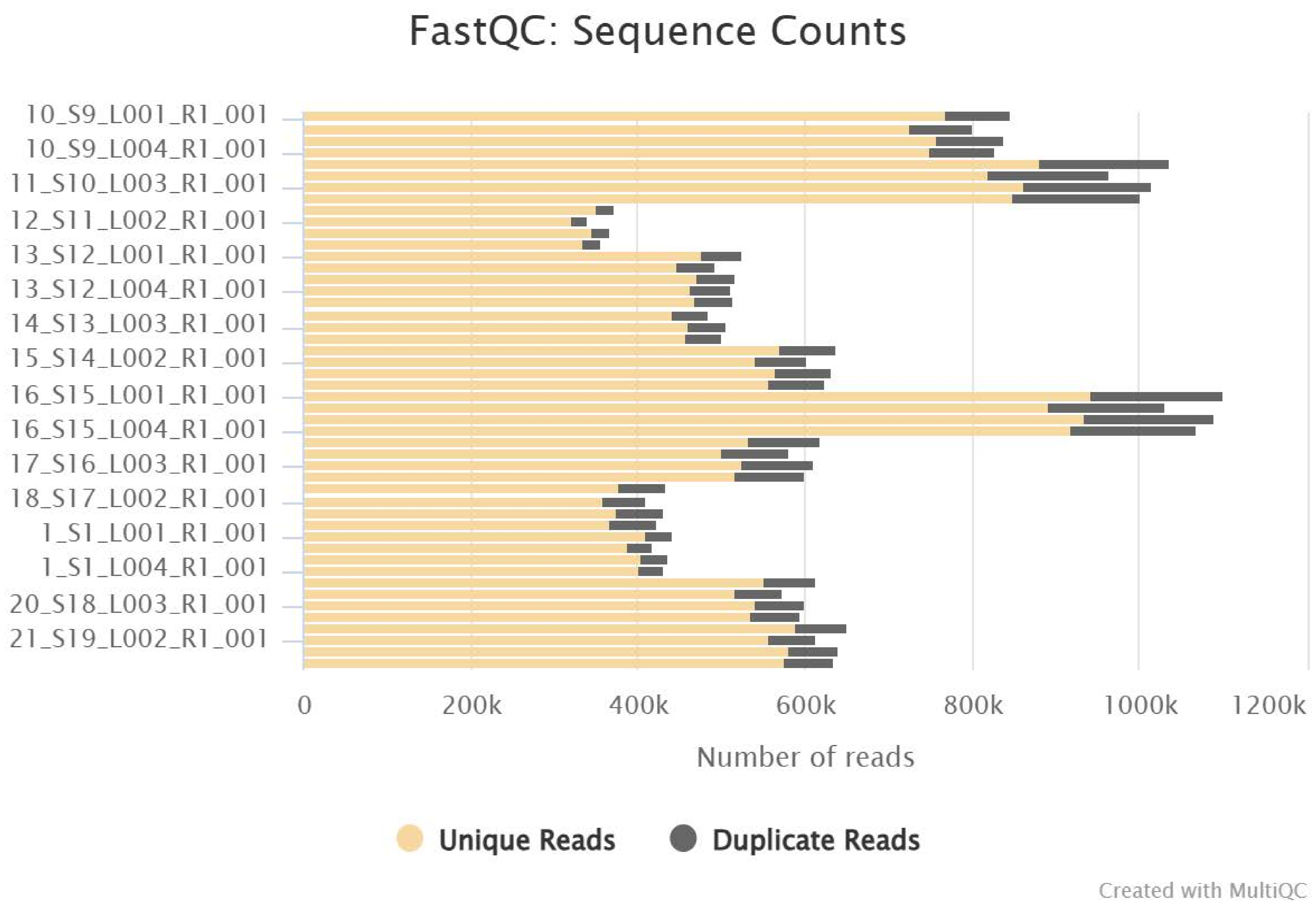
FastQC: Sequence Counts
10_S9_L001_R1_001
10_S9_L004_R1_001
11_S10_L003_R1_001
12_S11_L002_R1_001
13_S12_L001_R1_001
13_S12_L004_R1_001
14_S13_L003_R1_001
15_S14_L002_R1_001
16_S15_L001_R1_001
16_S15_L004_R1_001
17_S16_L003_R1_001
18_S17_L002_R1_001
1_S1_L001_R1_001
1_S1_L004_R1_001
20_S18_L003_R1_001
21_S19_L002_R1_001
0
200k
400k
600k
800k
1000k
1200k
Number of reads
Unique Reads
Duplicate Reads
Created with MultiQC
