# Supplementary material for: Psilocybin elicits a conserved glucocorticoid-responsive gene signature across five 5-HT2A receptor-rich brain regions in rat
Source: Acta Neuropsychiatr. 2026 Apr 10;38:e37. doi: 10.1017/neu.2026.10075 (PMC13202413; doi:10.1017/neu.2026.10075)
Supplement: Veysi et al. supplementary material 10 — Veysi et al. supplementary material [file S0924270826100751sup010.pdf]

Supplement X

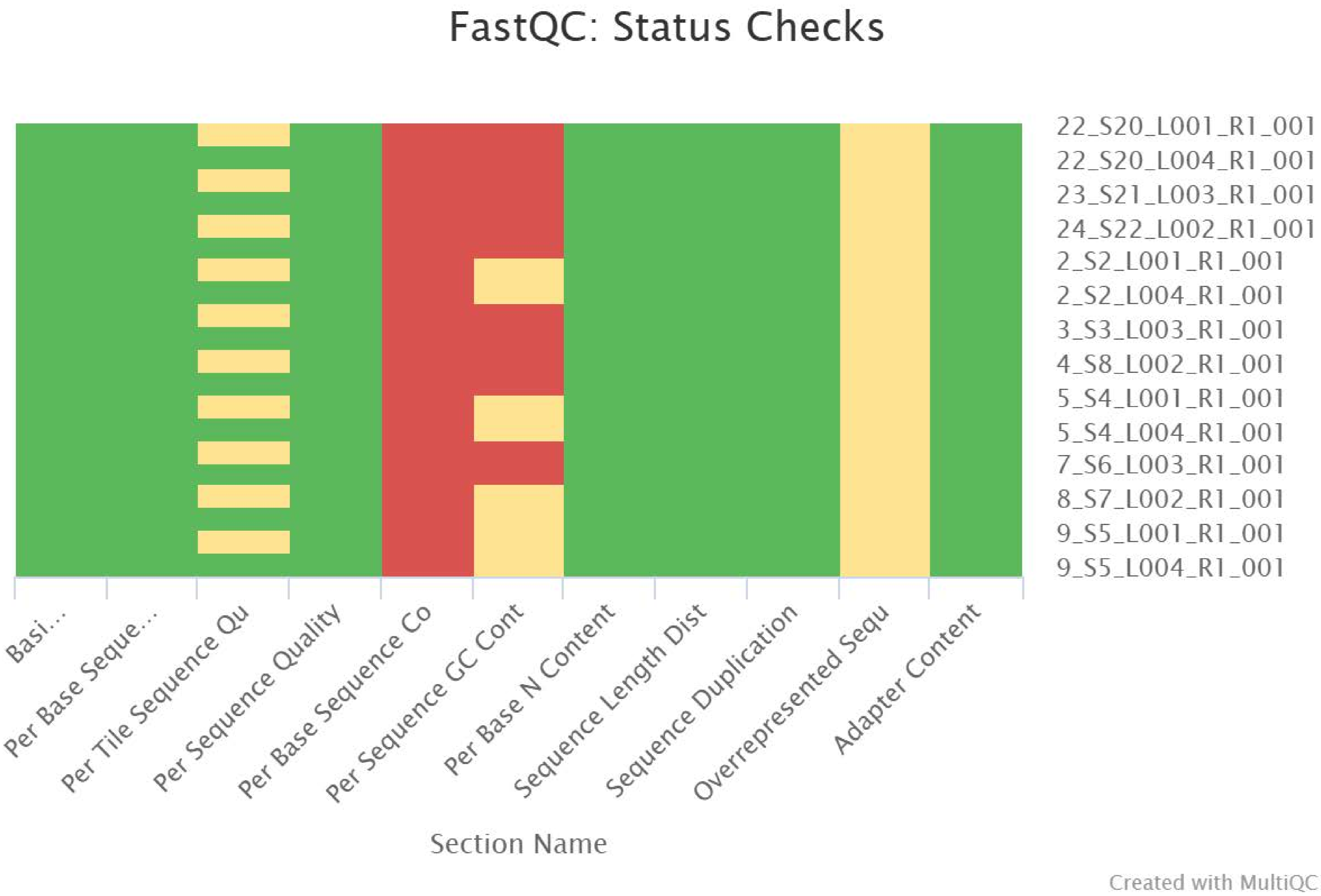

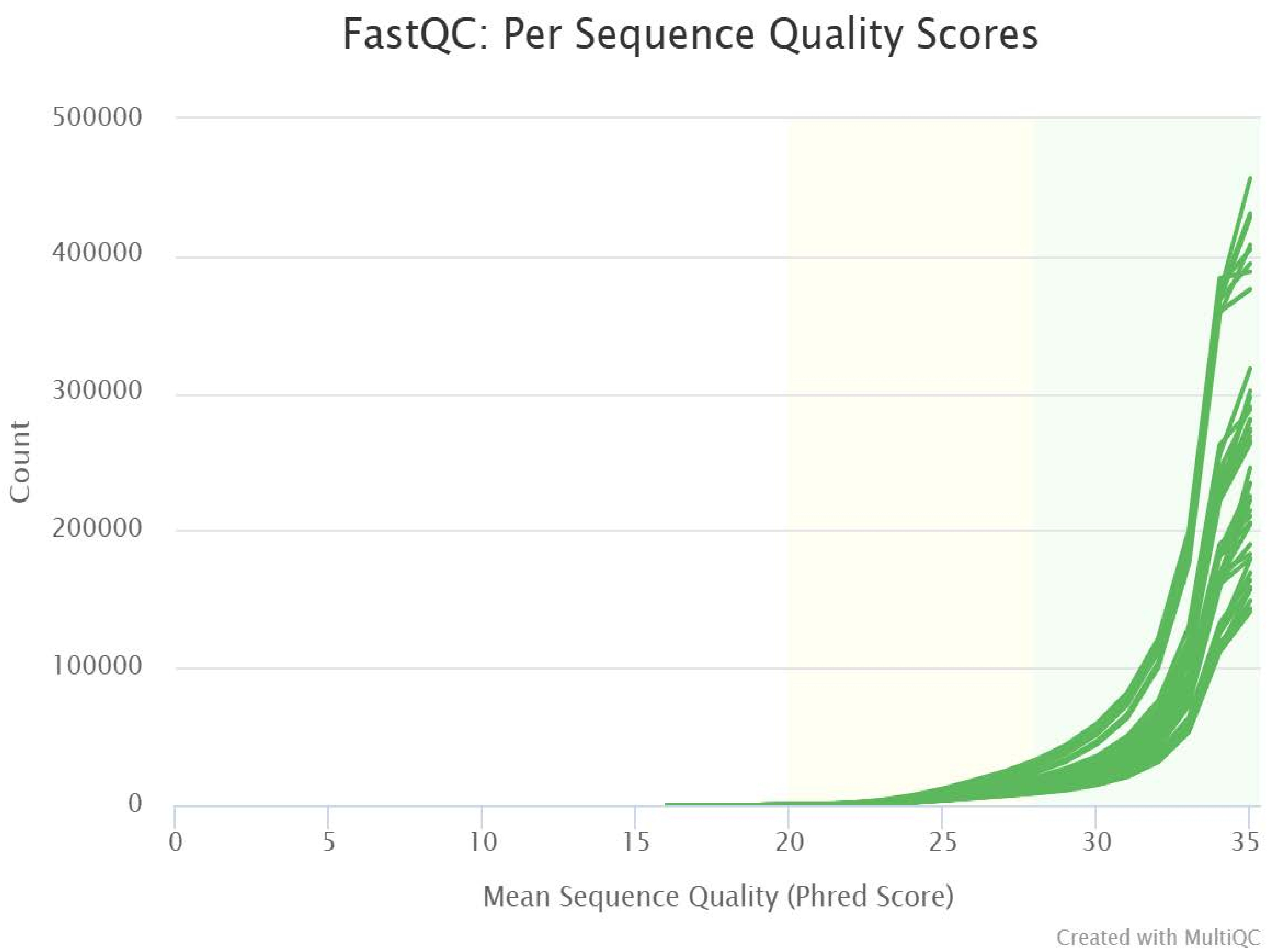

FastQC: Per Sequence Quality Scores
500000
400000
300000
200000
100000
0
Count
0
5
10
15
20
25
30
35
Mean Sequence Quality (Phred Score)
Created with MultiQC

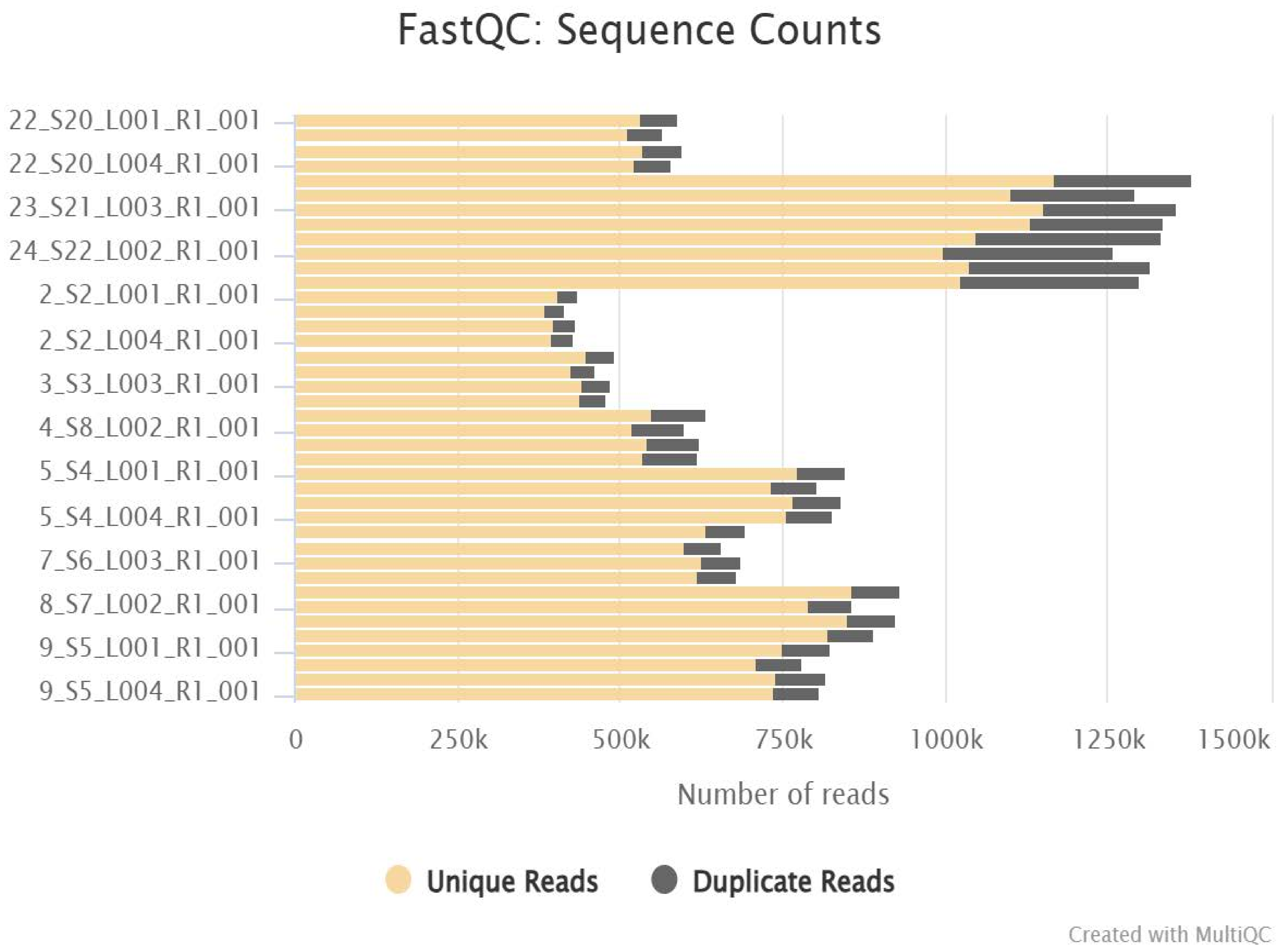

FastQC: Sequence Counts
22_S20_L001_R1_001
22_S20_L004_R1_001
23_S21_L003_R1_001
24_S22_L002_R1_001
2_S2_L001_R1_001
2_S2_L004_R1_001
3_S3_L003_R1_001
4_S8_L002_R1_001
5_S4_L001_R1_001
5_S4_L004_R1_001
7_S6_L003_R1_001
8_S7_L002_R1_001
9_S5_L001_R1_001
9_S5_L004_R1_001
0
250k
500k
750k
1000k
1250k
1500k
Number of reads
Unique Reads
Duplicate Reads
Created with MultiQC

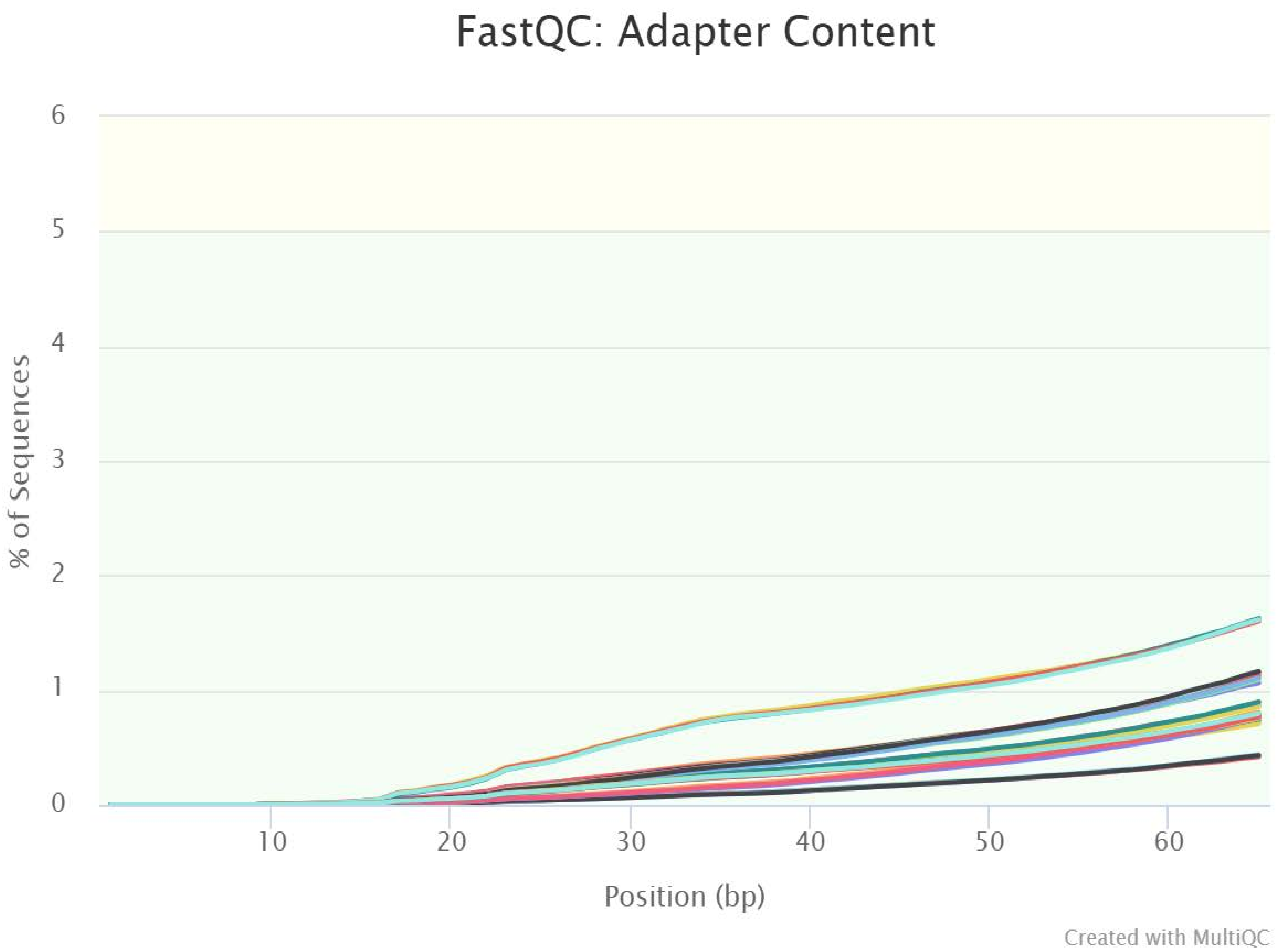
FastQC: Adapter Content
% of Sequences
0
1
2
3
4
5
6
10
20
30
40
50
60
Position (bp)
Created with MultiQC

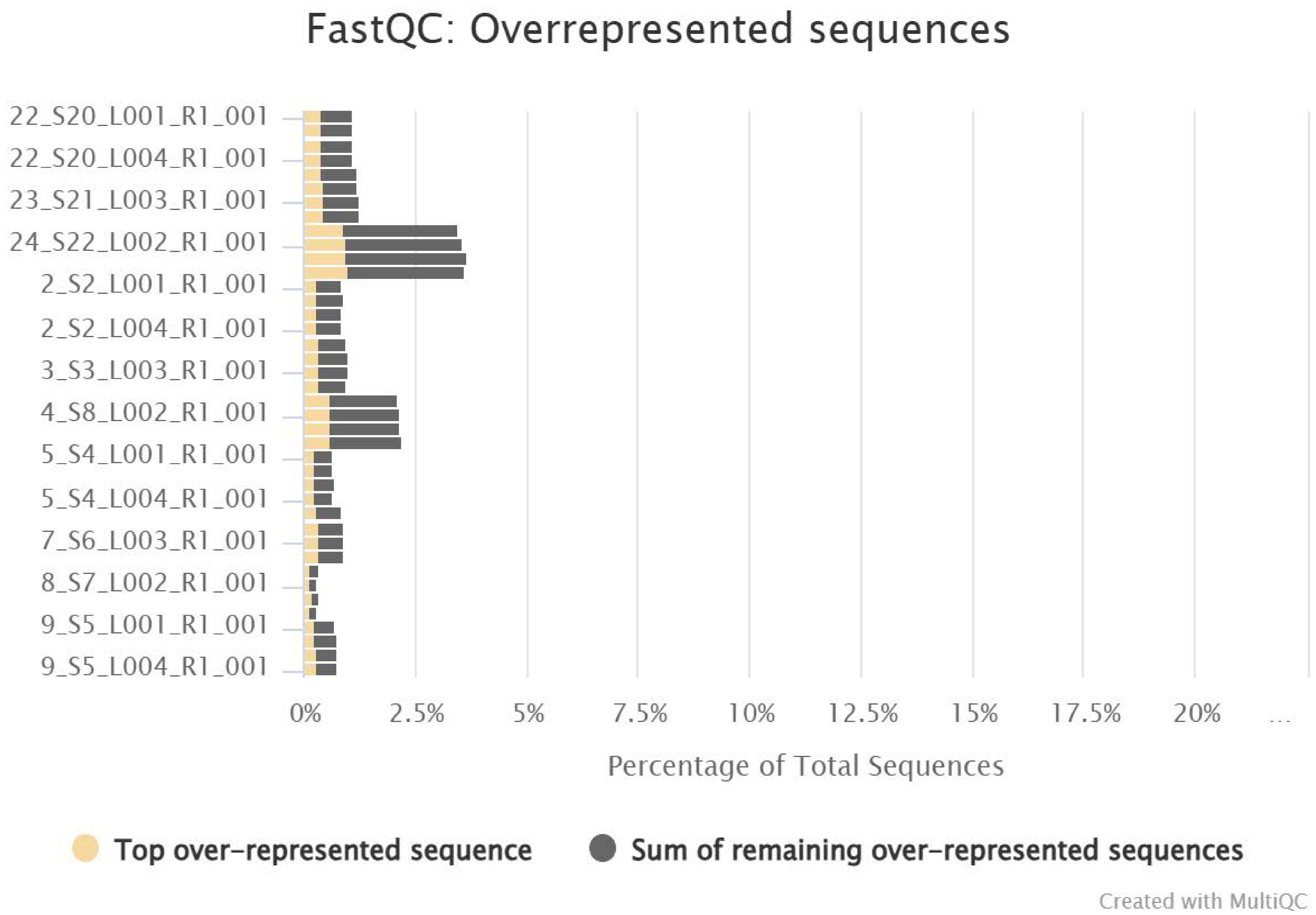
FastQC: Overrepresented sequences
22_S20_L001_R1_001
22_S20_L004_R1_001
23_S21_L003_R1_001
24_S22_L002_R1_001
2_S2_L001_R1_001
2_S2_L004_R1_001
3_S3_L003_R1_001
4_S8_L002_R1_001
5_S4_L001_R1_001
5_S4_L004_R1_001
7_S6_L003_R1_001
8_S7_L002_R1_001
9_S5_L001_R1_001
9_S5_L004_R1_001
0%
2.5%
5%
7.5%
10%
12.5%
15%
17.5%
20%
...
Percentage of Total Sequences
Top over-represented sequence
Sum of remaining over-represented sequences
Created with MultiQC

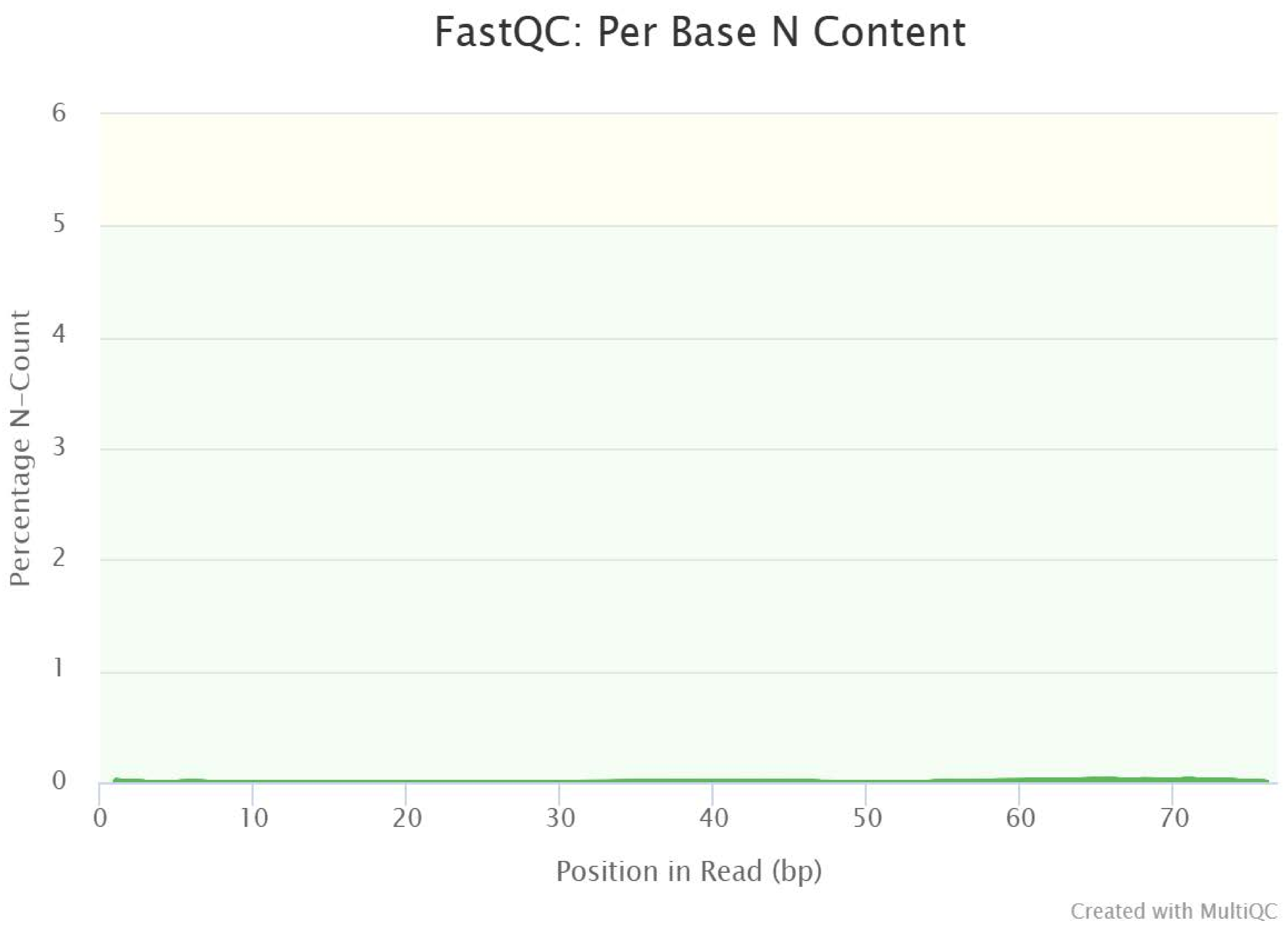
FastQC: Per Base N Content
Percentage N-Count
0
1
2
3
4
5
6
Position in Read (bp)
0
10
20
30
40
50
60
70
Created with MultiQC

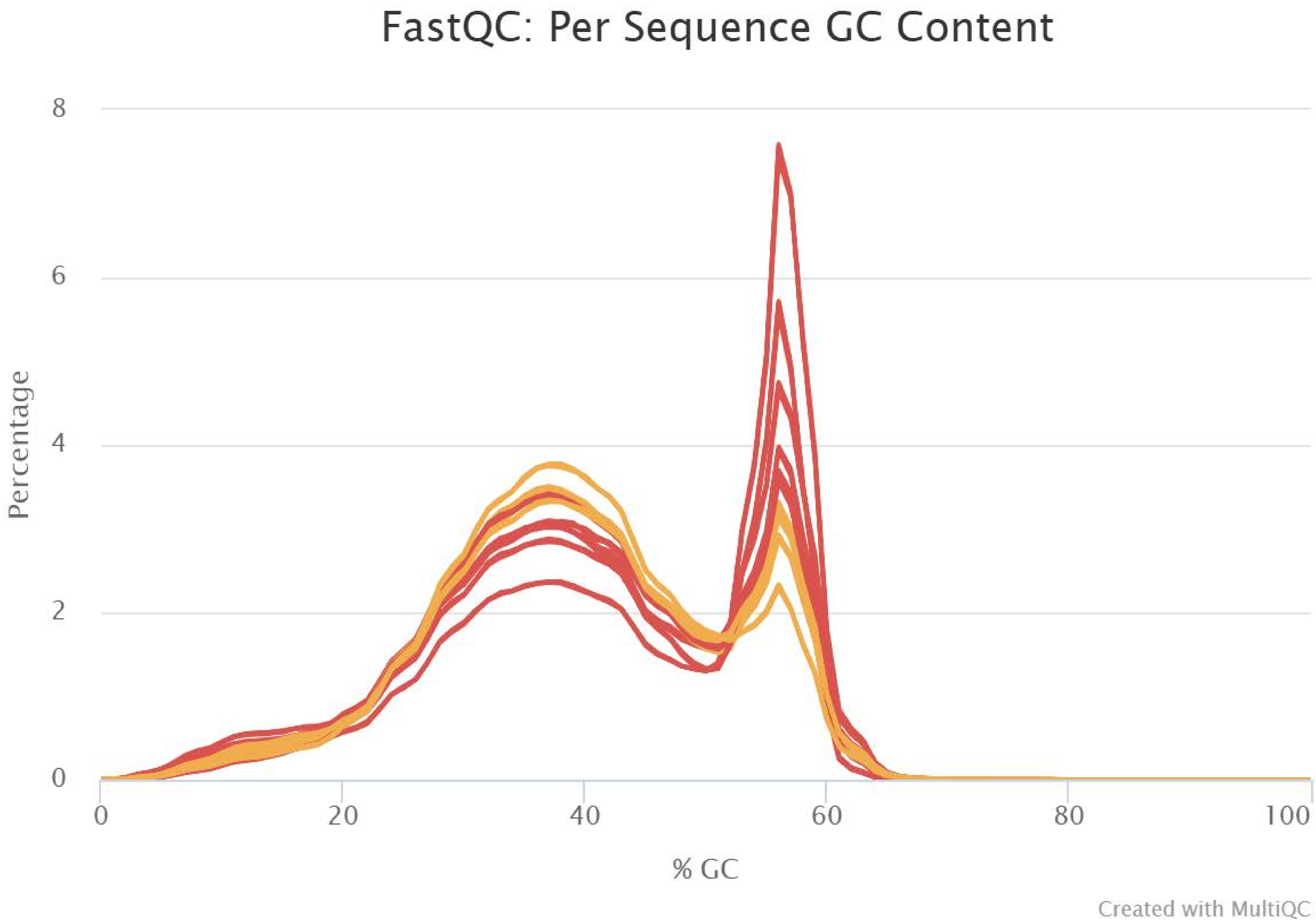
FastQC: Per Sequence GC Content
Percentage
8
6
4
2
0
0
20
40
60
80
100
% GC
Created with MultiQC

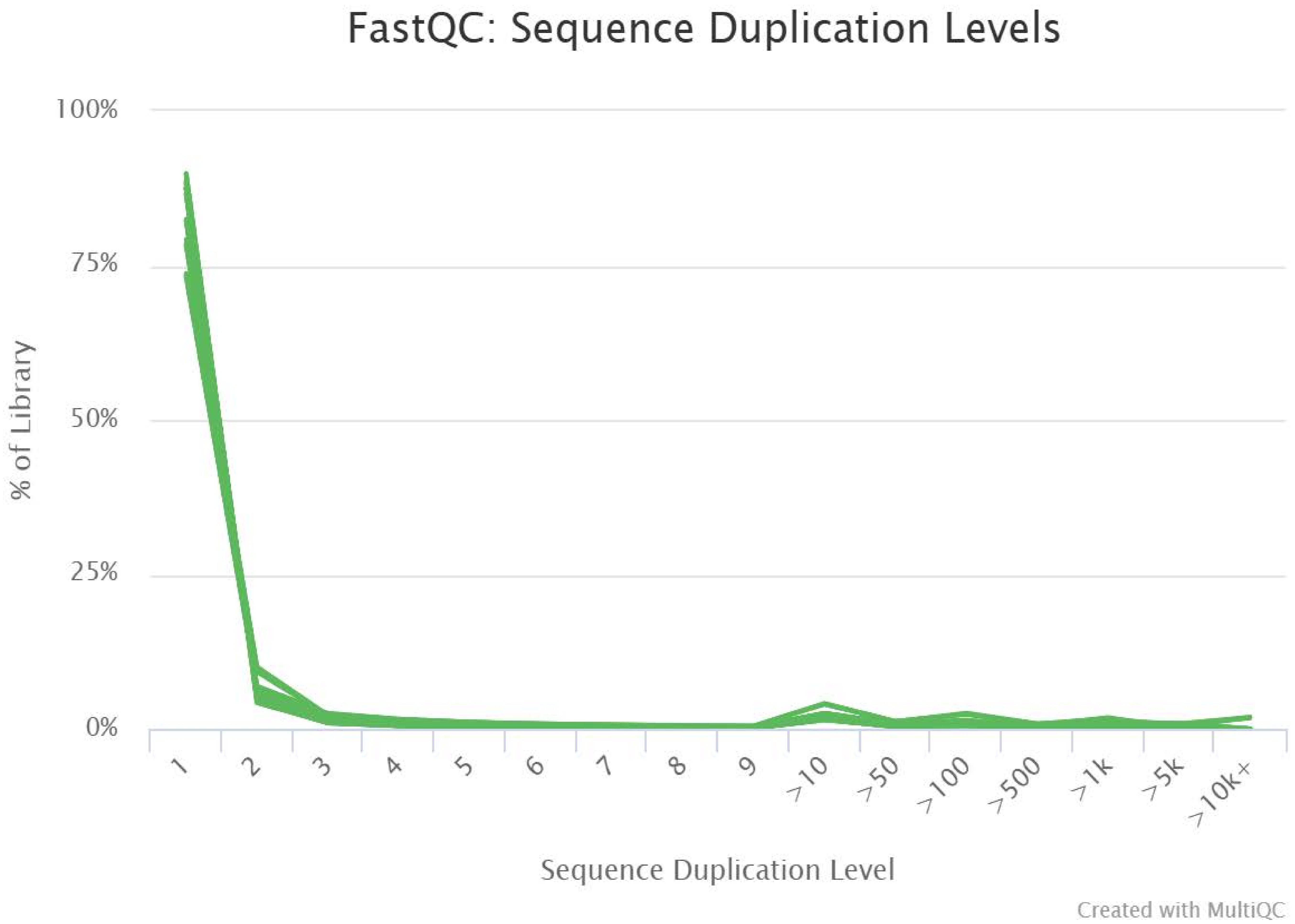
FastQC: Sequence Duplication Levels
100%
75%
50%
25%
0%
% of Library
1
2
3
4
5
6
7
8
9
>10
>50
>100
>500
>1k
>5k
>10k+
Sequence Duplication Level
Created with MultiQC

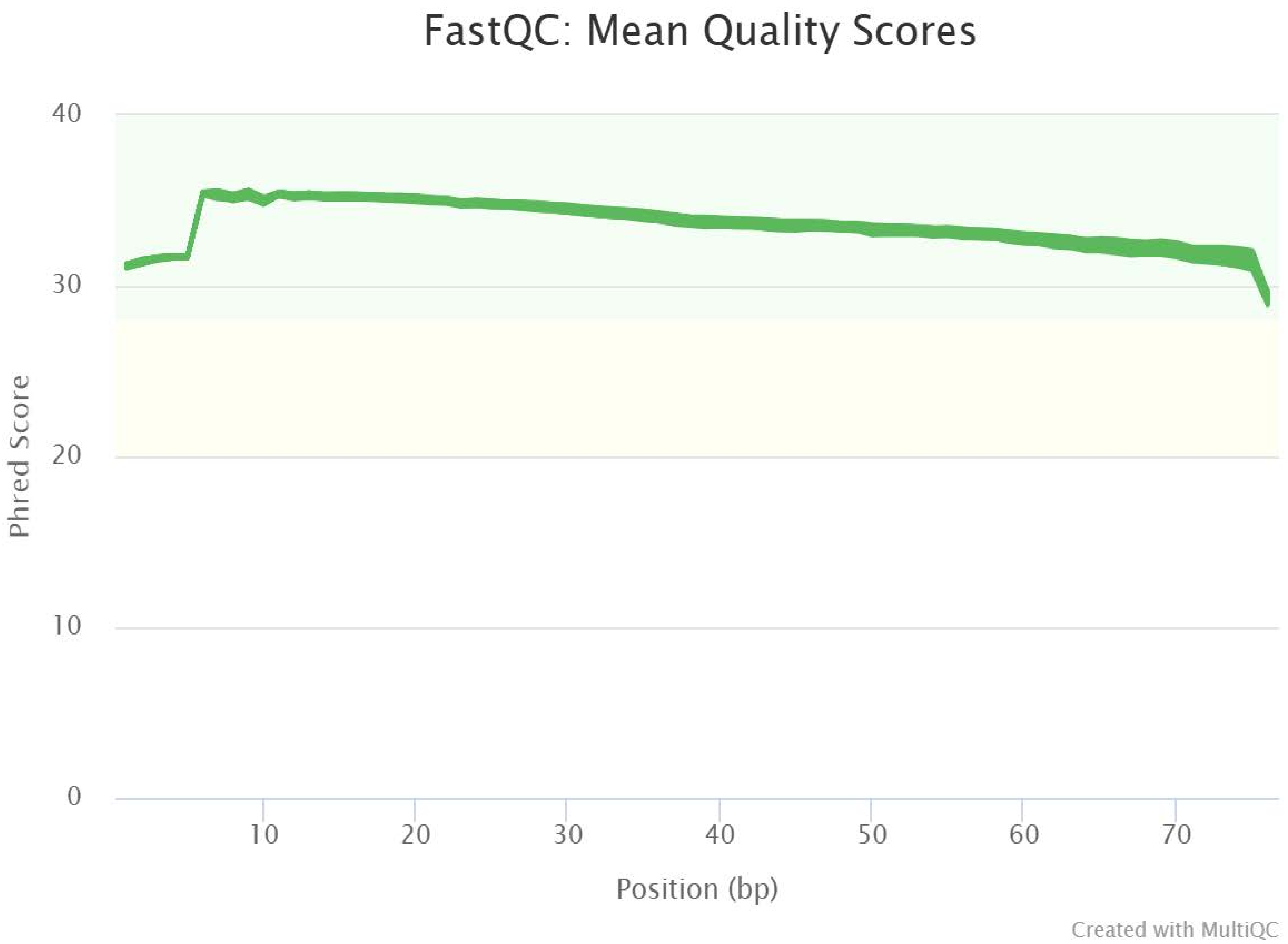
FastQC: Mean Quality Scores
40
30
20
10
0
Phred Score
10
20
30
40
50
60
70
Position (bp)
Created with MultiQC
