# Supplementary material for: Psilocybin elicits a conserved glucocorticoid-responsive gene signature across five 5-HT2A receptor-rich brain regions in rat
Source: Acta Neuropsychiatr. 2026 Apr 10;38:e37. doi: 10.1017/neu.2026.10075 (PMC13202413; doi:10.1017/neu.2026.10075)
Supplement: Veysi et al. supplementary material 11 — Veysi et al. supplementary material [file S0924270826100751sup011.pdf]

Supplement XI

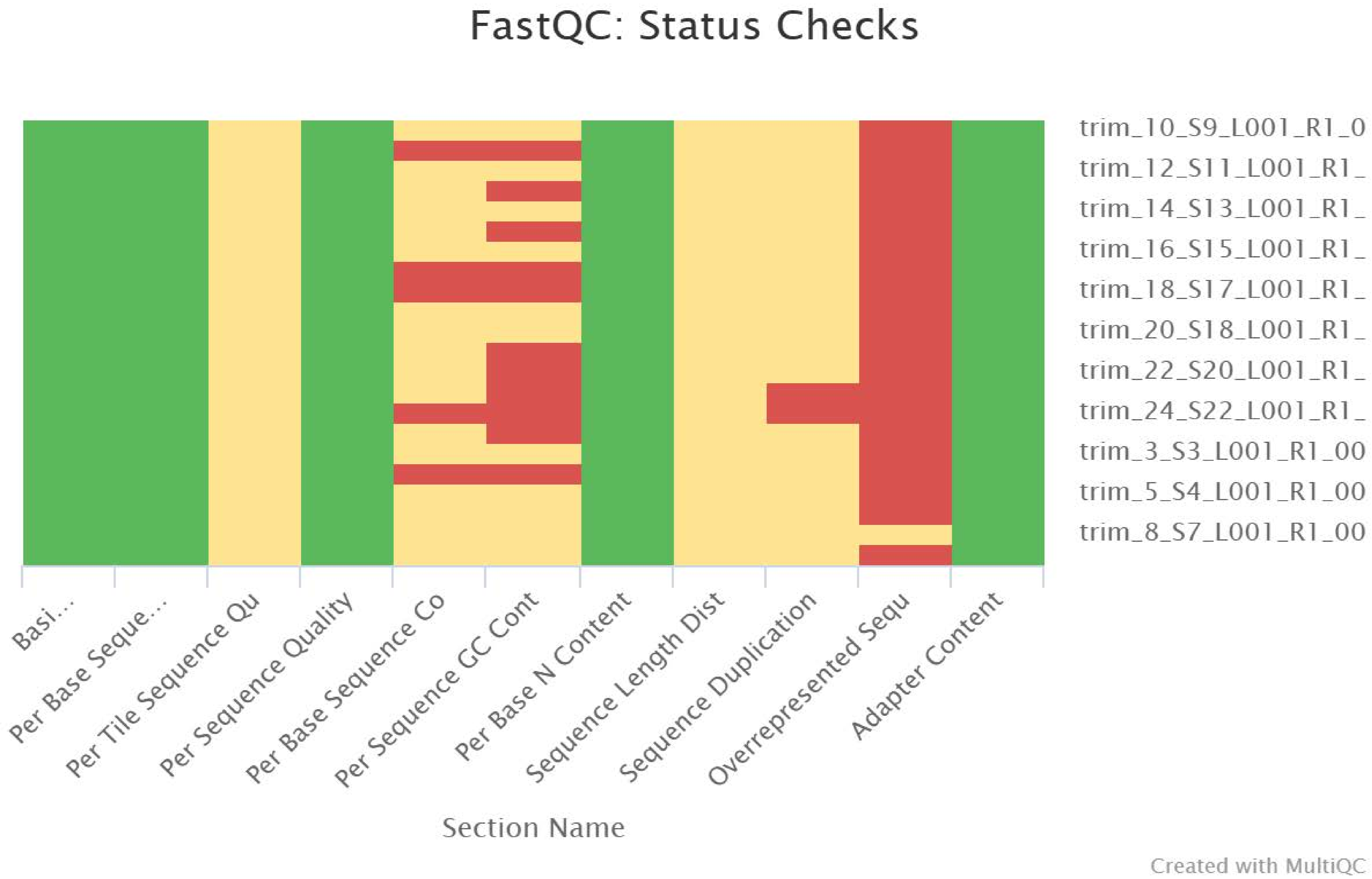

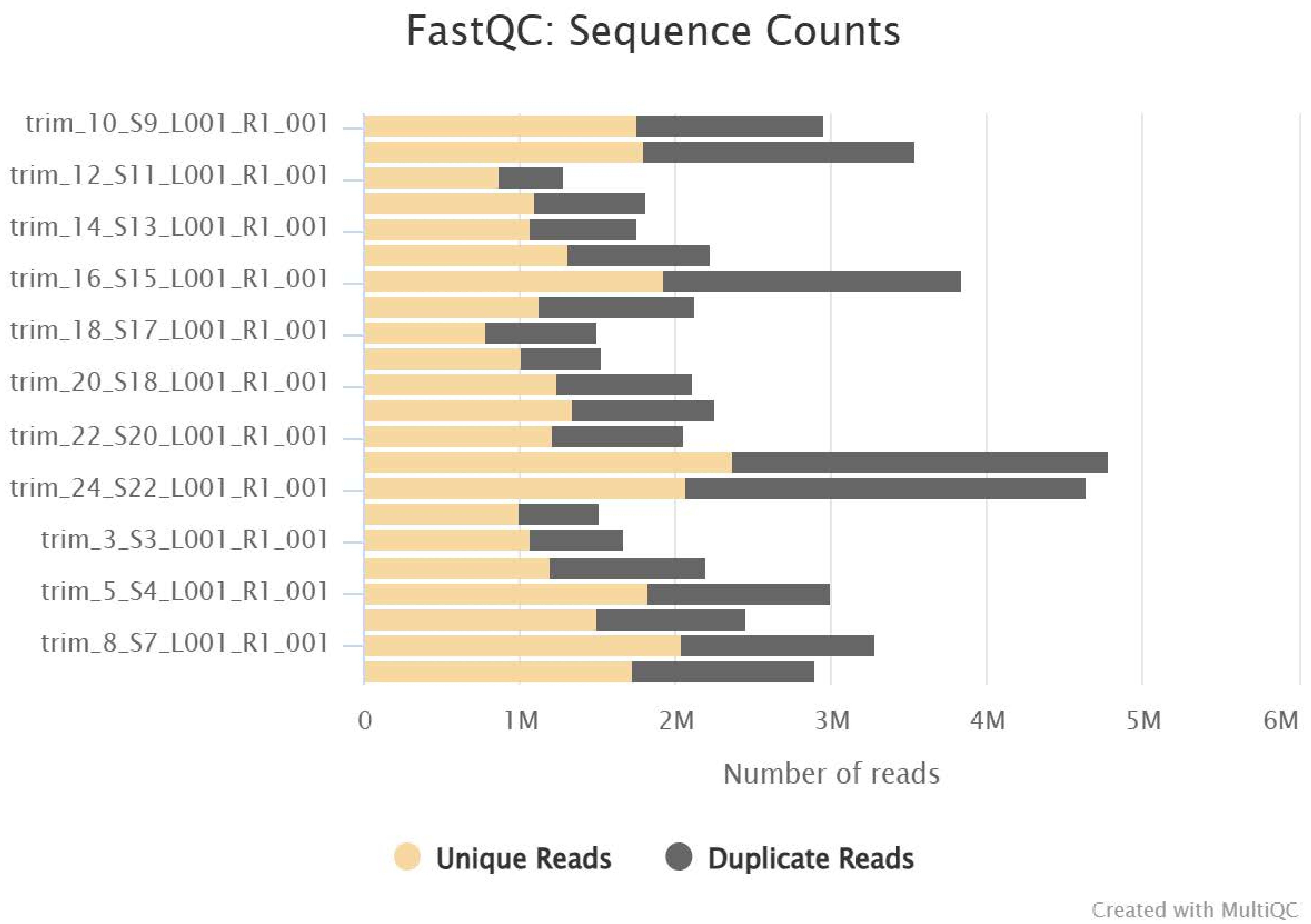
FastQC: Sequence Counts
trim_10_S9_L001_R1_001
trim_12_S11_L001_R1_001
trim_14_S13_L001_R1_001
trim_16_S15_L001_R1_001
trim_18_S17_L001_R1_001
trim_20_S18_L001_R1_001
trim_22_S20_L001_R1_001
trim_24_S22_L001_R1_001
trim_3_S3_L001_R1_001
trim_5_S4_L001_R1_001
trim_8_S7_L001_R1_001
0
1M
2M
3M
4M
5M
6M
Number of reads
Unique Reads
Duplicate Reads
Created with MultiQC

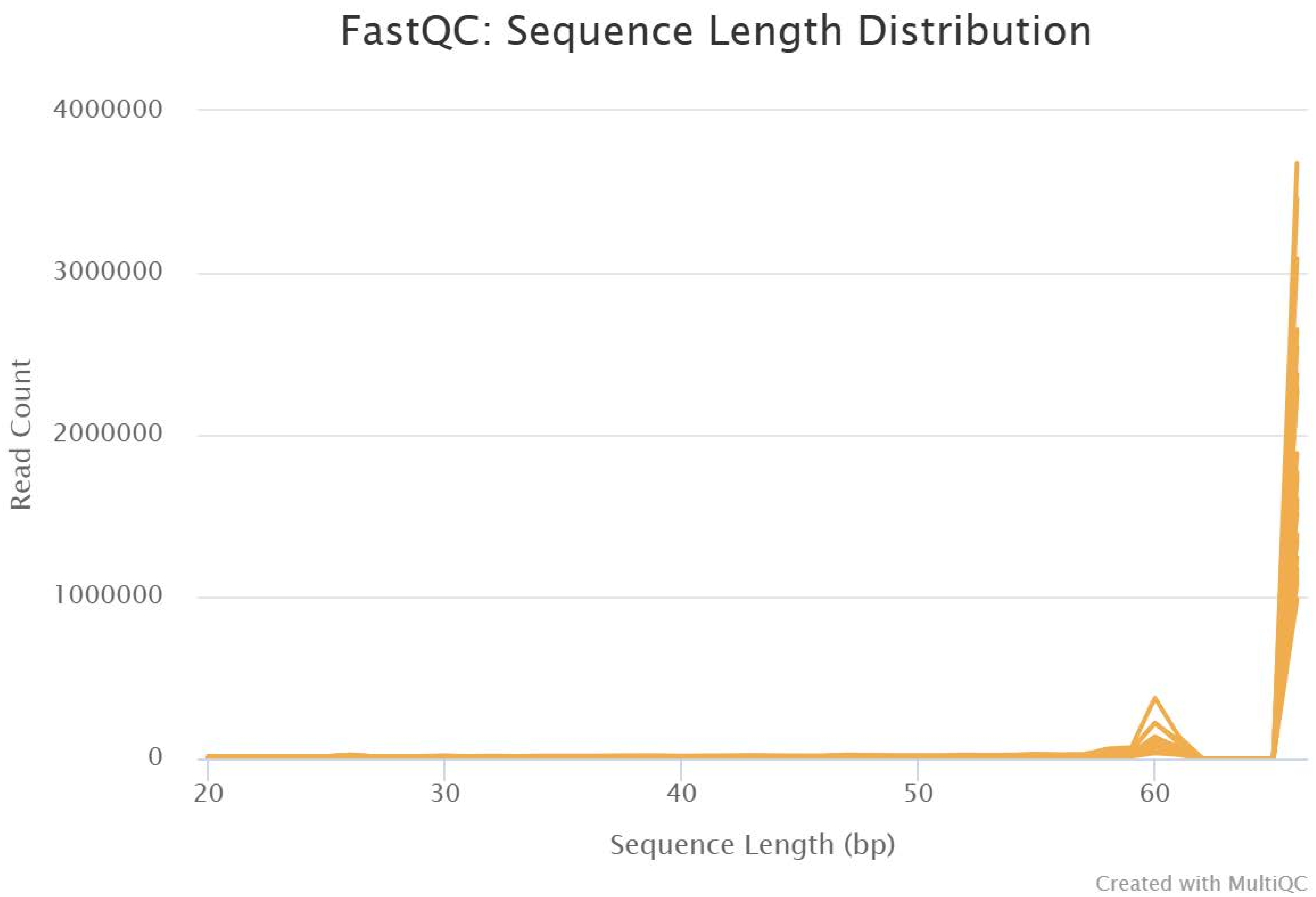

FastQC: Sequence Length Distribution
4000000
3000000
2000000
1000000
0
Read Count
20
30
40
50
60
Sequence Length (bp)
Created with MultiQC

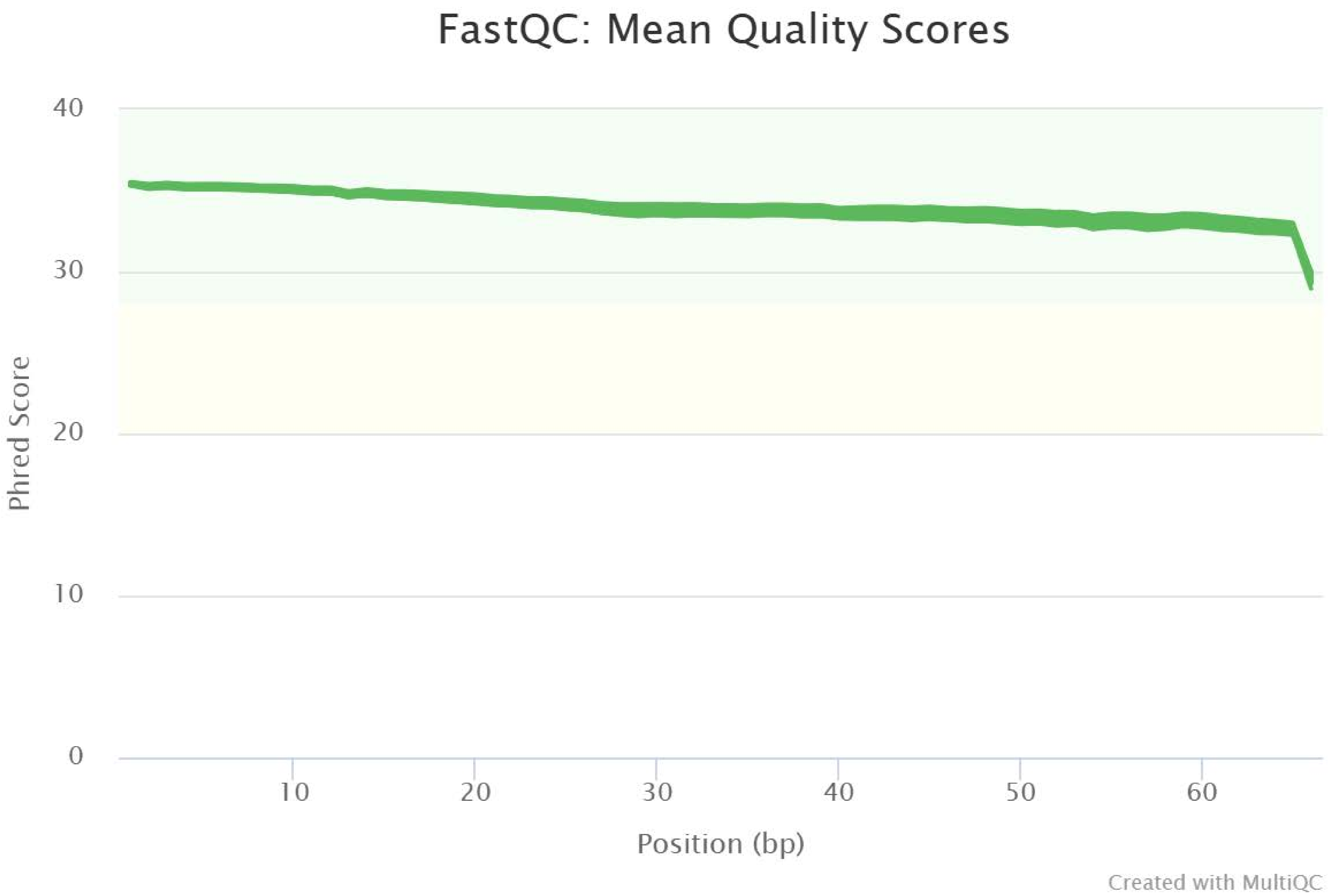
FastQC: Mean Quality Scores
40
30
20
10
0
Phred Score
10
20
30
40
50
60
Position (bp)
Created with MultiQC

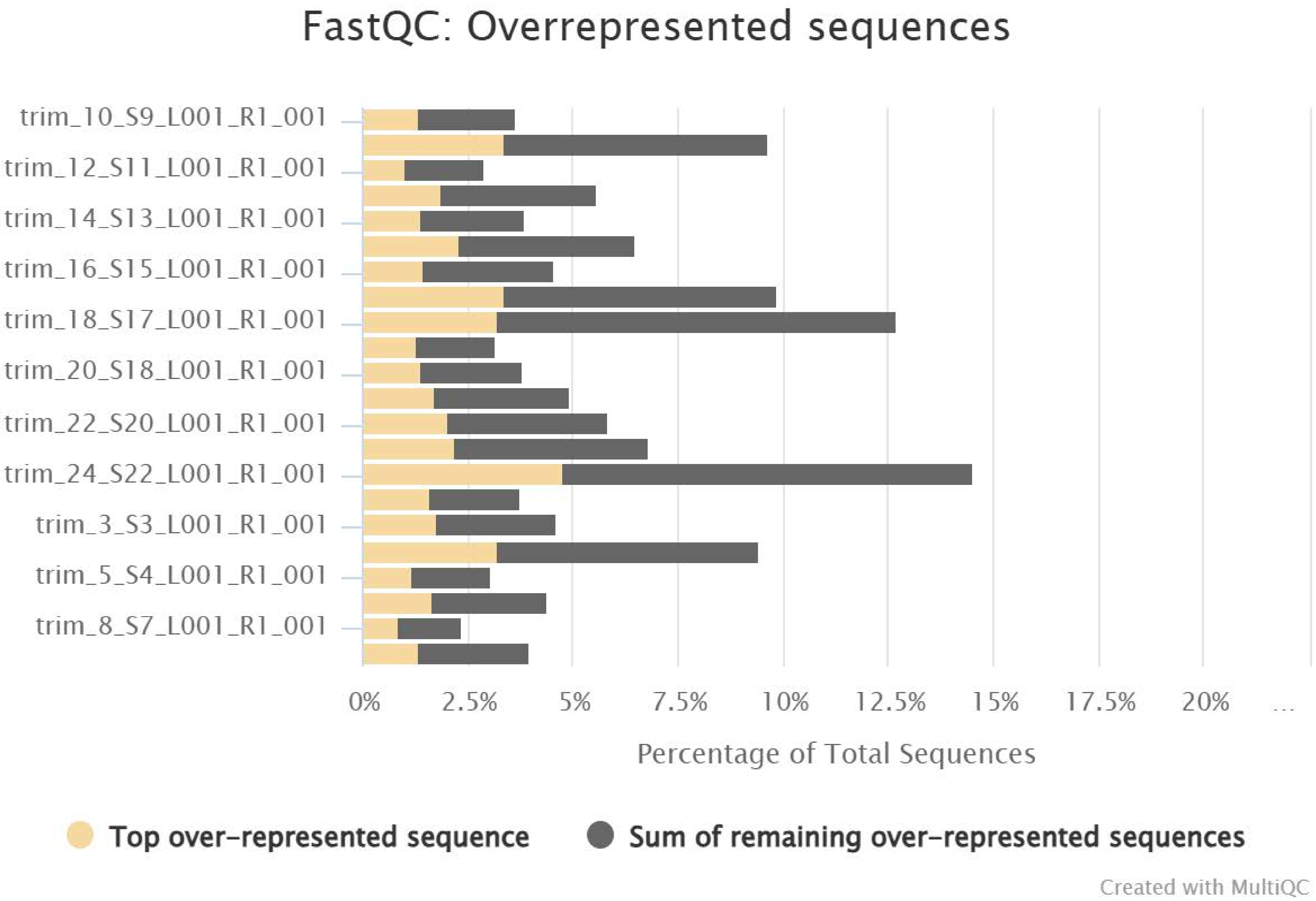

FastQC: Overrepresented sequences
trim_10_S9_L001_R1_001
trim_12_S11_L001_R1_001
trim_14_S13_L001_R1_001
trim_16_S15_L001_R1_001
trim_18_S17_L001_R1_001
trim_20_S18_L001_R1_001
trim_22_S20_L001_R1_001
trim_24_S22_L001_R1_001
trim_3_S3_L001_R1_001
trim_5_S4_L001_R1_001
trim_8_S7_L001_R1_001
0%
2.5%
5%
7.5%
10%
12.5%
15%
17.5%
20%
...
Percentage of Total Sequences
Top over-represented sequence
Sum of remaining over-represented sequences
Created with MultiQC

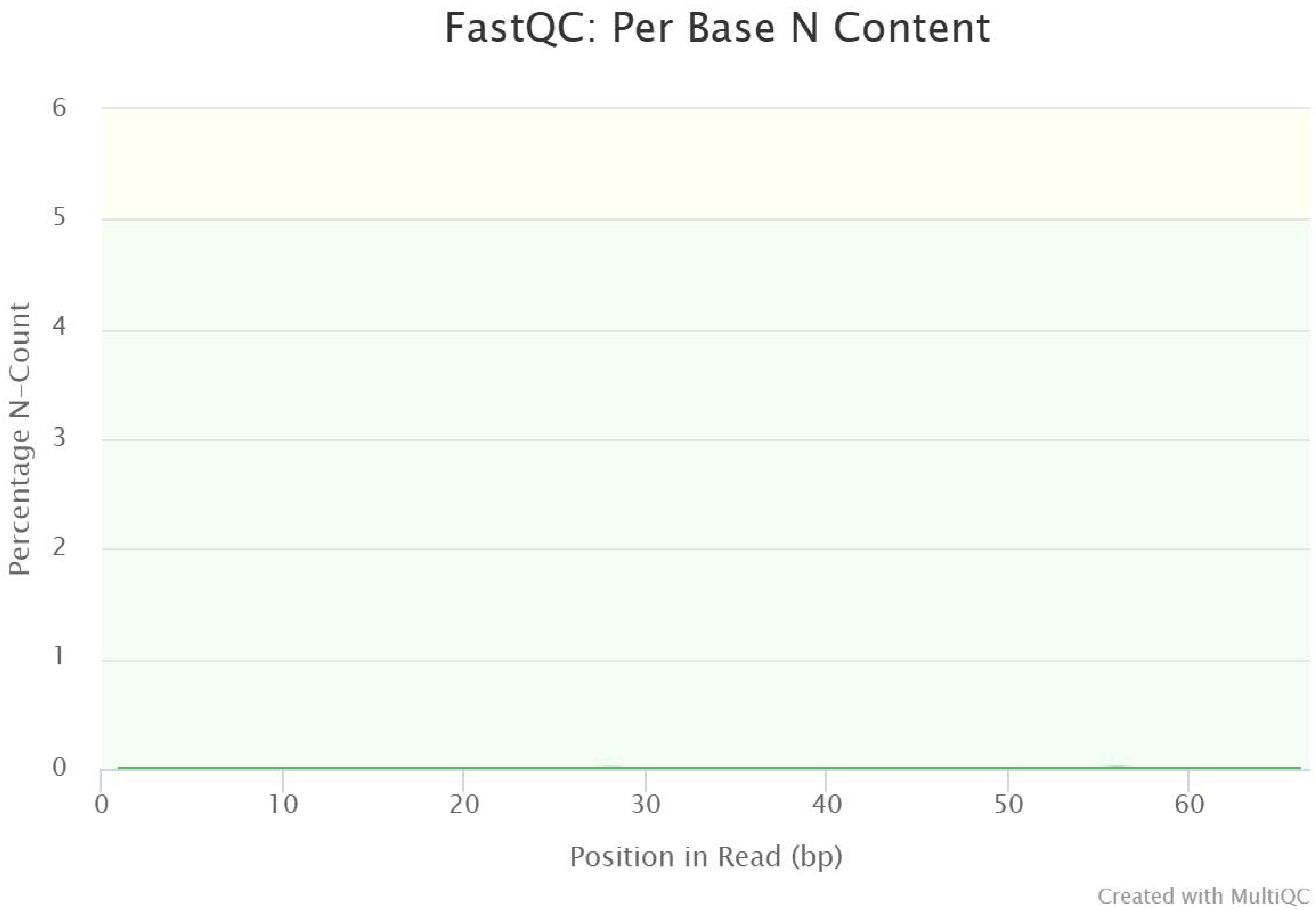
FastQC: Per Base N Content
6
5
4
3
2
1
0
Percentage N-Count
0
10
20
30
40
50
60
Position in Read (bp)
Created with MultiQC

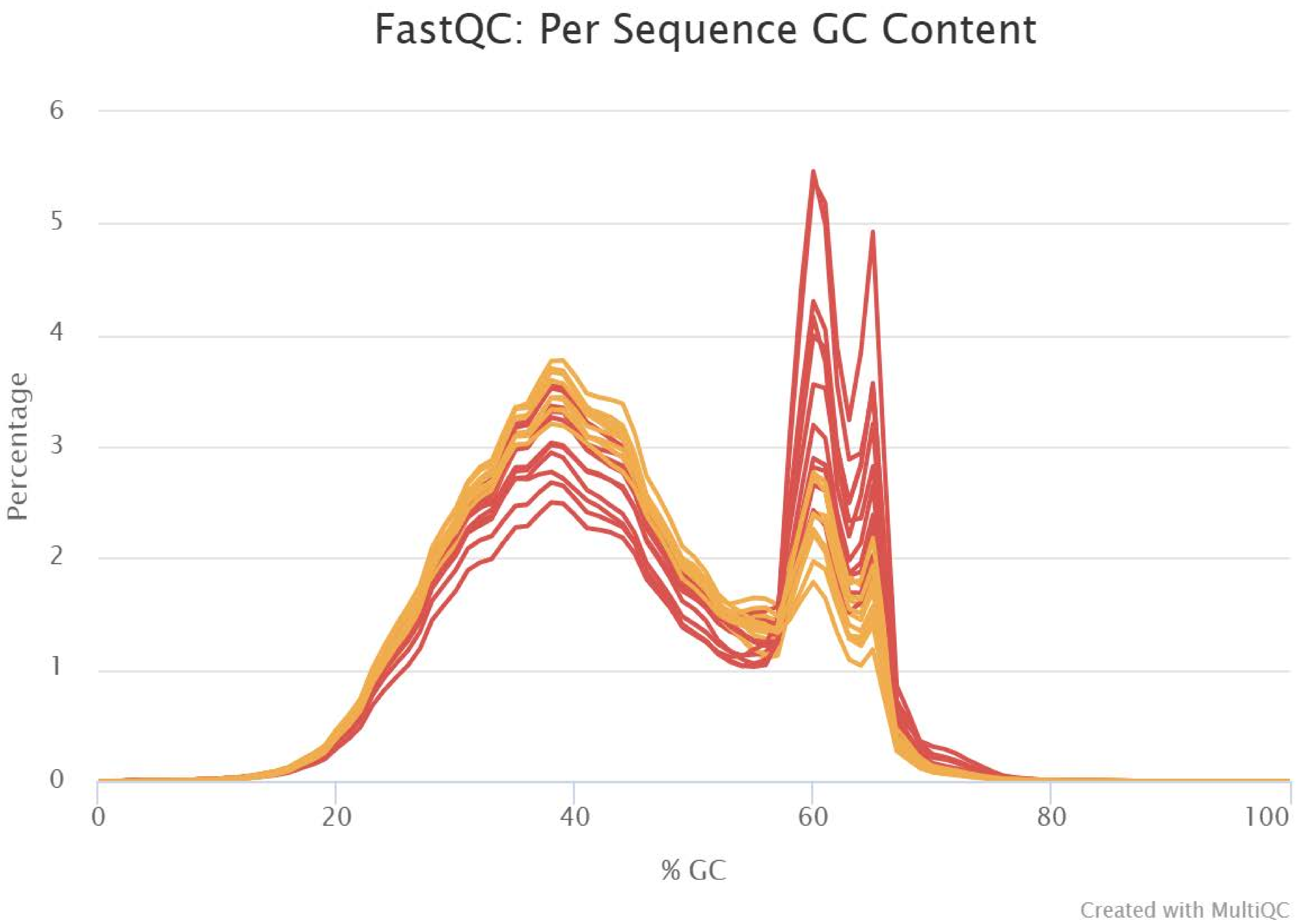
FastQC: Per Sequence GC Content
Percentage
0
1
2
3
4
5
6
0
20
40
60
80
100
% GC
Created with MultiQC

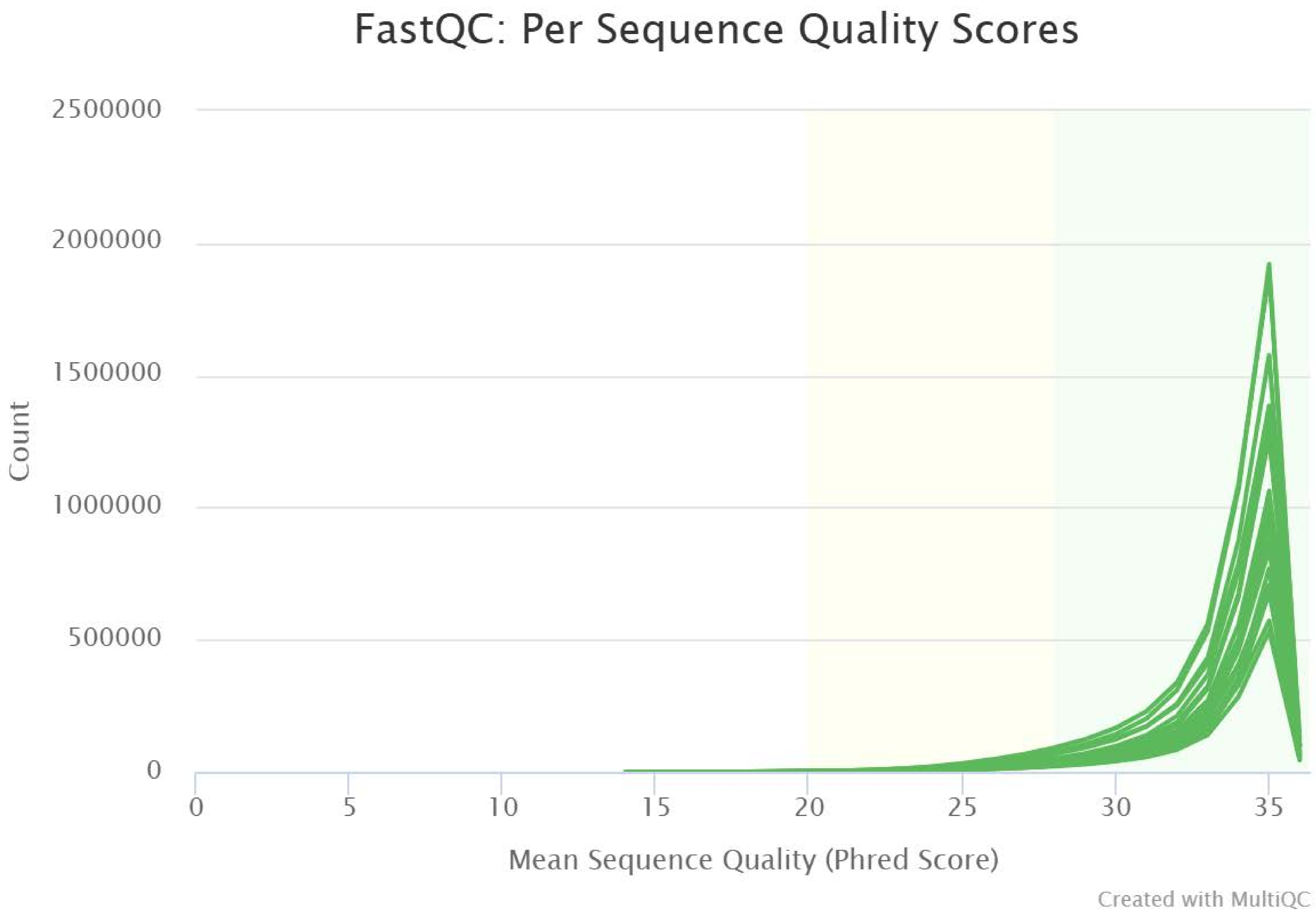
FastQC: Per Sequence Quality Scores
2500000
2000000
1500000
1000000
500000
0
Count
0
5
10
15
20
25
30
35
Mean Sequence Quality (Phred Score)
Created with MultiQC

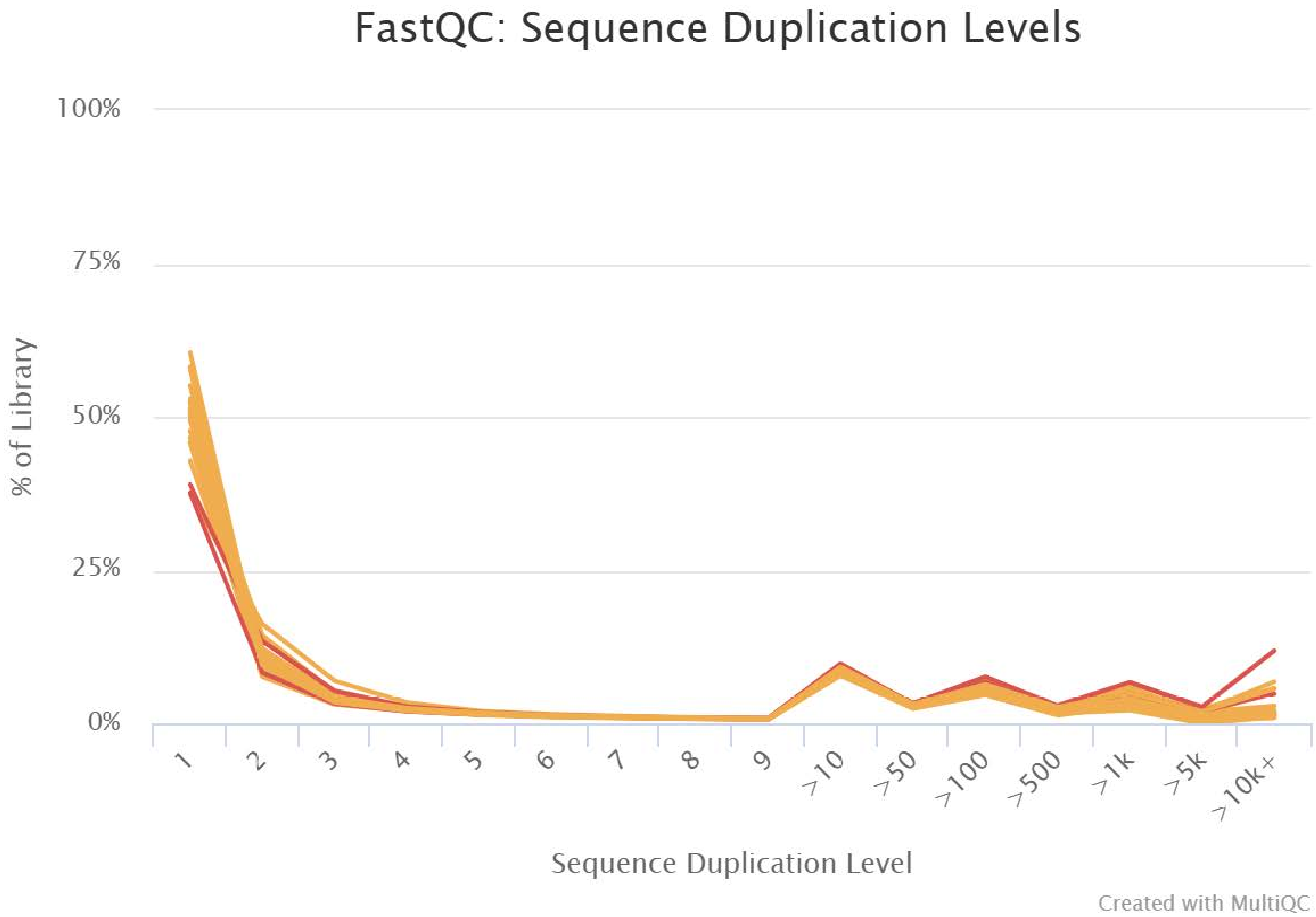

FastQC: Sequence Duplication Levels
100%
75%
50%
25%
0%
% of Library
1
2
3
4
5
6
7
8
9
>10
>50
>100
>500
>1k
>5k
>10k+
Sequence Duplication Level
Created with MultiQC
