# Supplementary material for: Psilocybin elicits a conserved glucocorticoid-responsive gene signature across five 5-HT2A receptor-rich brain regions in rat
Source: Acta Neuropsychiatr. 2026 Apr 10;38:e37. doi: 10.1017/neu.2026.10075 (PMC13202413; doi:10.1017/neu.2026.10075)
Supplement: Veysi et al. supplementary material 12 — Veysi et al. supplementary material [file S0924270826100751sup012.pdf]

Supplement XII

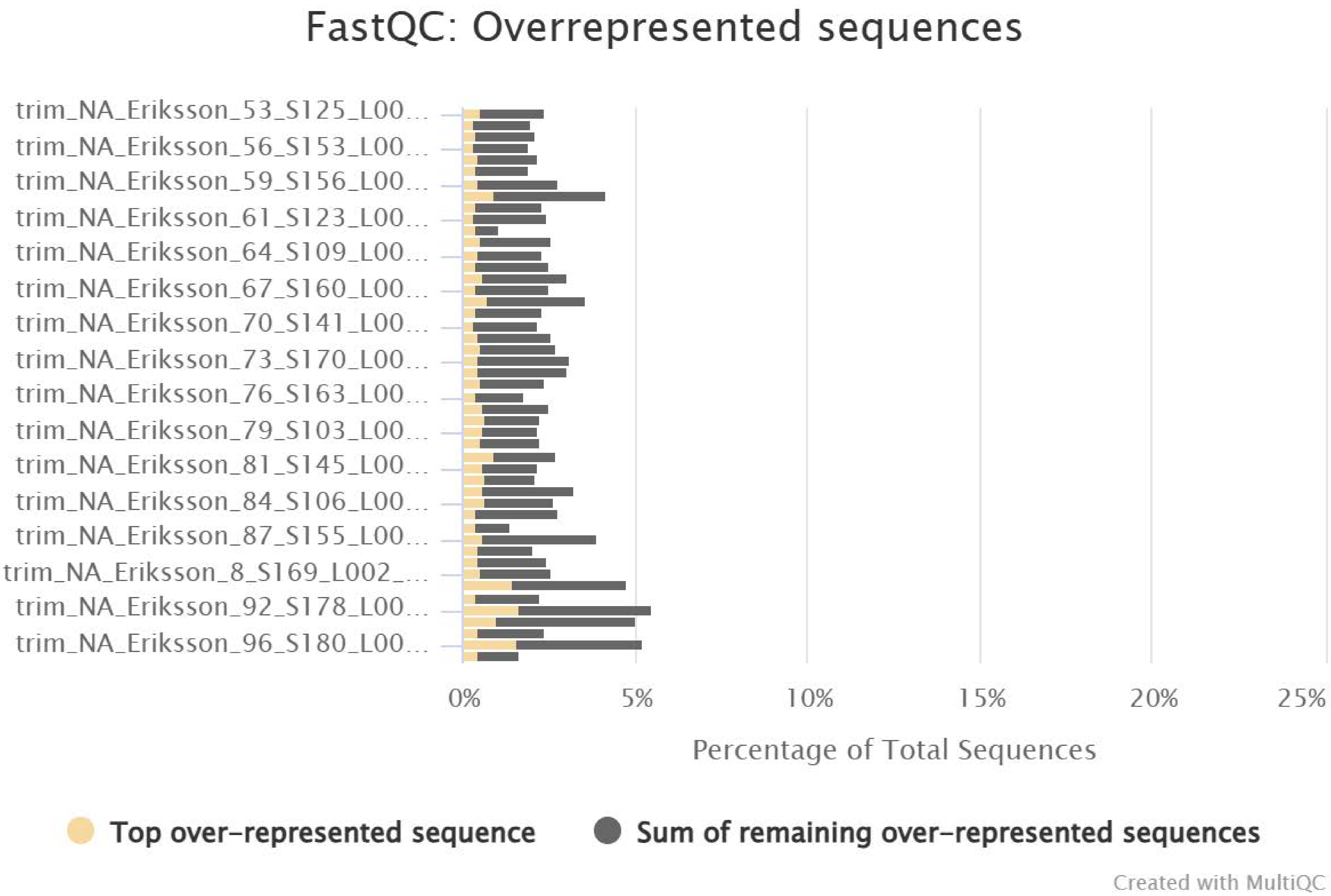

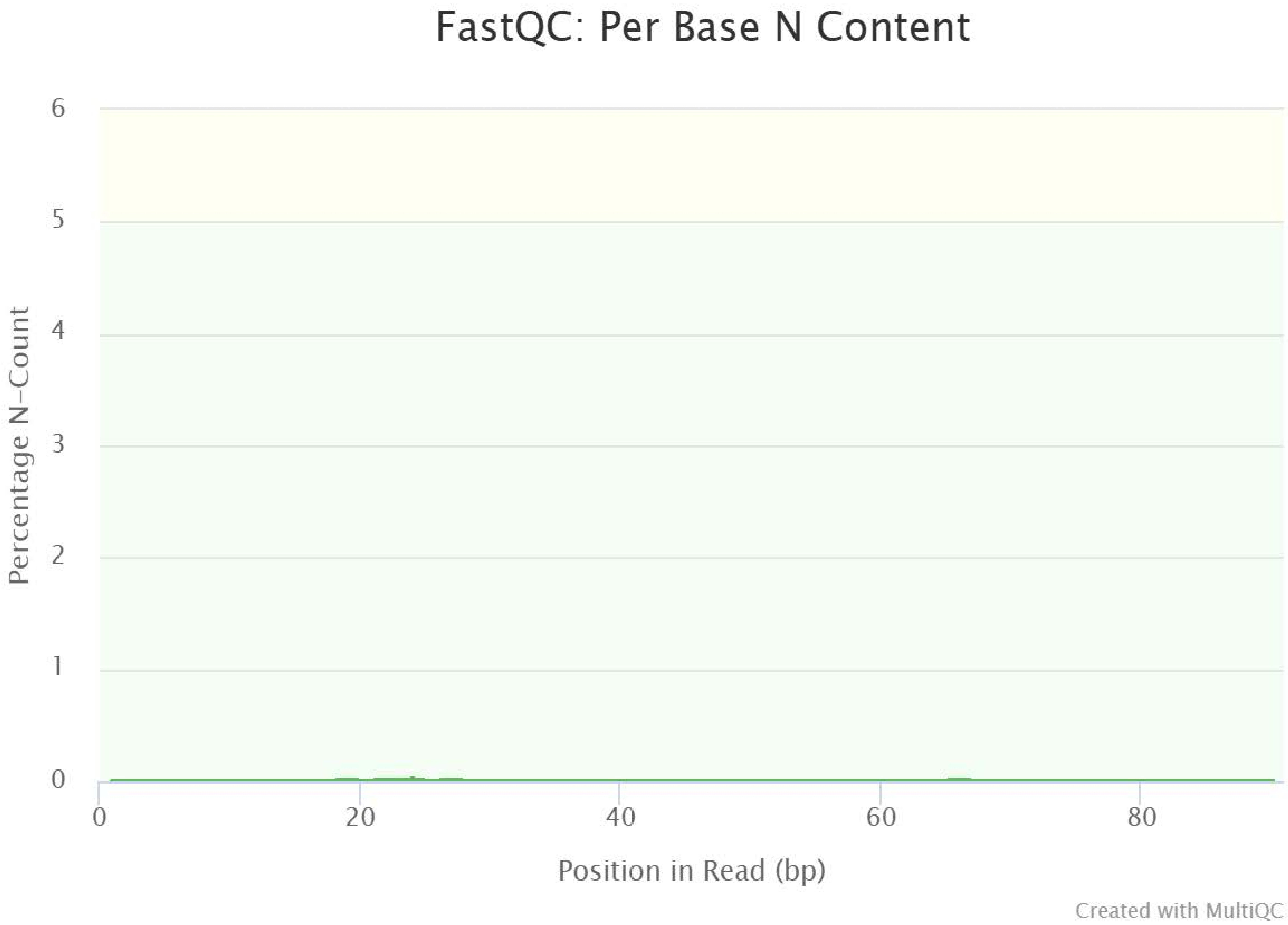
FastQC: Per Base N Content
Percentage N-Count
6
5
4
3
2
1
0
0
20
40
60
80
Position in Read (bp)
Created with MultiQC
